# Elephant bones for the Middle Pleistocene toolmaker

**DOI:** 10.1371/journal.pone.0256090

**Published:** 2021-08-26

**Authors:** Paola Villa, Giovanni Boschian, Luca Pollarolo, Daniela Saccà, Fabrizio Marra, Sebastien Nomade, Alison Pereira

**Affiliations:** 1 University of Colorado Museum of Natural History, Boulder, Colorado, United States of America; 2 Istituto Italiano di Paleontologia Umana, Rome, Italy; 3 Dipartimento di Biologia, Università di Pisa, Pisa, Italy; 4 School of Geography, Archaeology and Environmental Studies, University of the Witwatersrand, Johannesburg, South Africa; 5 Laboratoire Archéologie et Peuplement de l’Afrique, University of Geneva, Geneva, Switzerland; 6 Dipartimento di Civiltà e Forme del Sapere, University of Pisa, Pisa, Italy; 7 Istituto Nazionale di Geofisica e Vulcanologia, Rome, Italy; 8 Laboratoire des Sciences du Climat et de l’Environnement, Université Paris-Saclay, Gif-sur-Yvette, France; 9 Université Paris-Saclay, CNRS Laboratoire GEOPS, Orsay, France; 10 Departement Hommes et Environnements, UMR 7194 HNHP, Muséum national d’Histoire naturelle, Paris, France; Universita degli Studi di Ferrara, ITALY

## Abstract

The use of bone as raw material for implements is documented since the Early Pleistocene. Throughout the Early and Middle Pleistocene bone tool shaping was done by percussion flaking, the same technique used for knapping stone artifacts, although bone shaping was rare compared to stone tool flaking. Until recently the generally accepted idea was that early bone technology was essentially immediate and expedient, based on single-stage operations, using available bone fragments of large to medium size animals. Only Upper Paleolithic bone tools would involve several stages of manufacture with clear evidence of primary flaking or breaking of bone to produce the kind of fragments required for different kinds of tools. Our technological and taphonomic analysis of the bone assemblage of Castel di Guido, a Middle Pleistocene site in Italy, now dated by ^40^Ar/^39^Ar to about 400 ka, shows that this general idea is inexact. In spite of the fact that the number of bone bifaces at the site had been largely overestimated in previous publications, the number of verified, human-made bone tools is 98. This is the highest number of flaked bone tools made by pre-modern hominids published so far. Moreover the Castel di Guido bone assemblage is characterized by systematic production of standardized blanks (elephant diaphysis fragments) and clear diversity of tool types. Bone smoothers and intermediate pieces prove that some features of Aurignacian technology have roots that go beyond the late Mousterian, back to the Middle Pleistocene. Clearly the Castel di Guido hominids had done the first step in the process of increasing complexity of bone technology. We discuss the reasons why this innovation was not developed. The analysis of the lithic industry is done for comparison with the bone industry.

## Introduction

The use of bone as raw material for implements in the Lower Paleolithic has been known since at least the 1970’s [1]. One bone handaxe was reported by M. Leakey from Olduvai Bed II [1: pl. 40] corresponding to an age of 1.65–1.3 million years (Ma) and another one is reported from the site of Konso in Ethiopia with an age of 1.4 Ma [2]. According to Mary Leakey there were 125 artificially modified bone and teeth mainly in Olduvai Bed II. The reanalysis by Backwell and d’Errico [3] reports a total of 36 bone tools almost all in Bed II with only one from DK, lower Bed 1 [3: Fig 28]. The tools were made mostly, but not exclusively, on long bone epiphyseal end or shafts of large or very large mammals. The tools are described as occasional and expedient.

**Fig 1:**
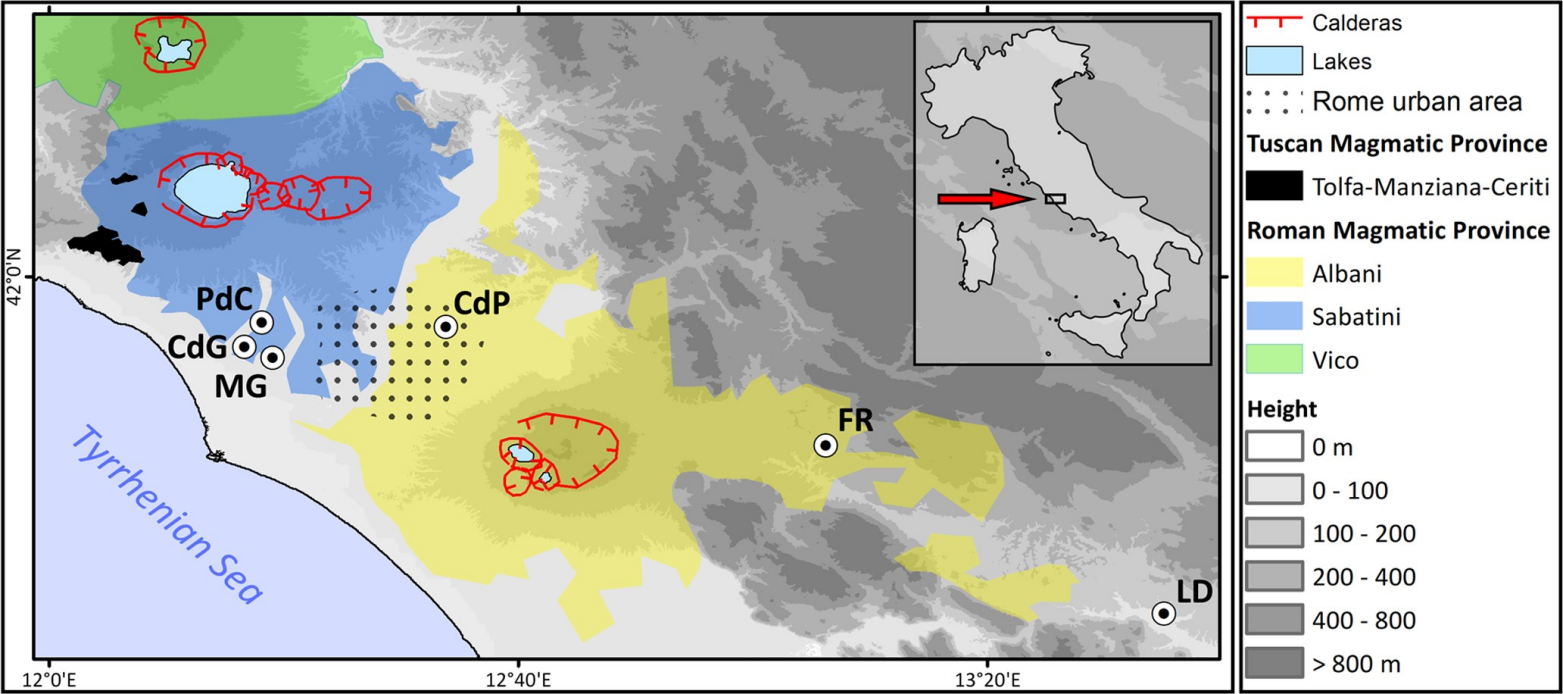
Location of sites mentioned in the text and in Table 1. CdG = Castel di Guido, MG = Malagrotta, PdC = Polledrara di Cecanibbio, CdP = Casal de’ Pazzi, FR = Fontana Ranuccio, LD = Lademagne. The Roman Magmatic Province [4, 5] is formed by large volcanic complexes, namely the Colli Albani, the Monti Sabatini, the Vico volcano and the Monti Volsini to the north (the last not shown on this map). Drawing by G. Boschian. Figure sources: Roman Magmatic Province outcrops simplified from Carta geologica informatizzata Regione Lazio 1: 25000, doi.org/10.13140/2.1.2667.8080 (available under Creative Commons Attribution 4.0 - CC BY 4.0) with updates from GB fieldwork. Topographic background from USGS Shuttle Radar Topography Mission (Courtesy of NASA/Jet Propulsion Laboratory Caltech; credit line requested by JPL policy).

Use of bone for tools has been reported for the Middle Pleistocene of Italy since the 1980’s. Besides Castel di Guido (CdG), the object of our study, tools made on elephant bone have been found at several Middle Pleistocene sites in Latium (west-central Italy; Table 1, Fig 1).

**Table 1:** Middle pleistocene latium sites with bone tools. Unretouched bone flakes are included.

Site and references	Age (ka BP)	No. of bone tools	Bone bifaces	Taxon
Fontana Ranuccio [6–8]	^40^Ar/^39^Ar 407 ± 4ka*	31 or more (includes at least three intermediate pieces and some pointed tools)	2 complete plus 1 partial biface and 2 bifacial fragments	Elephant but also equid, bovid and cervid bones were used.
Malagrotta [9] and S5, S10 Figs in S1 File	^40^Ar/^39^Ar ages range 378 ± 6 to 451 ± 2	10 (includes 2 intermediate pieces, 1 pointed tool, 1 scraper, 4 flakes and a tool fragment)	1	The biface is on elephant diaphysis, 1 wedge is on bovid size diaphysis. Taxon of other pieces undetermined
Lademagne upper layer [8, 10]	^40^Ar/^39^Ar between 388 ± 5 and 404 ± 5*	8 (includes 2 pointed tools)	0	2 pieces on elephant diaphyses, 1 on elephant rib. Other pieces undetermined
La Polledrara ** [11, 12]	^40^Ar/^39^Ar 324 ± 4*	8 (includes 3 intermediate pieces, 2 scrapers, 1 pointed uniface)	0	7 tools made on elephant diaphysis
Pontecorvo[6]	Acheulian, not dated	3 1 metapodial with removals on distal end, 1 scraper, 1 diaphysis with rounded edge	0	Equid metapodial, elephant diaphyses
Casal de’ Pazzi [13]	MIS 7	1	0	Elephant diaphysis fragment

*Age recalculated at 2ϭ analytical uncertainties according to the optimized calibration of the standard [14, 15]. See Geochronological and stratigraphic data in Supporting Information.

** The full name is Polledrara di Cecanibbio, Polledrara is used for brevity.

In the absence of complete publications the reported number of bone tools from Fontana Ranuccio, La Polledrara and Casal de’ Pazzi are estimates based on drawings or partial publications. Other sites in Latium have yielded bone tools that are not well documented or were in stratigraphic context but not from controlled excavations [6]. Some European and Western Asian occurrences have been reported but are either isolated pieces or series that have not undergone taphonomic analysis (S1 Table in S1 File).

A large number of long bones used as retouchers and metapodials used for hammering have been described from Schöningen 13 II-4, the Spear Horizon [16]. Schöningen 12 II-4, a locality 800–1000 m from the Spear Horizon and dated to MIS 9 as the Spear Horizon, has yielded some bone hammers, retouchers and anvils, but also some long bones with polished tips, described as smoothers [17]. These occurrences are considered in comparison with the Castel di Guido assemblage.

**Fig 2:**
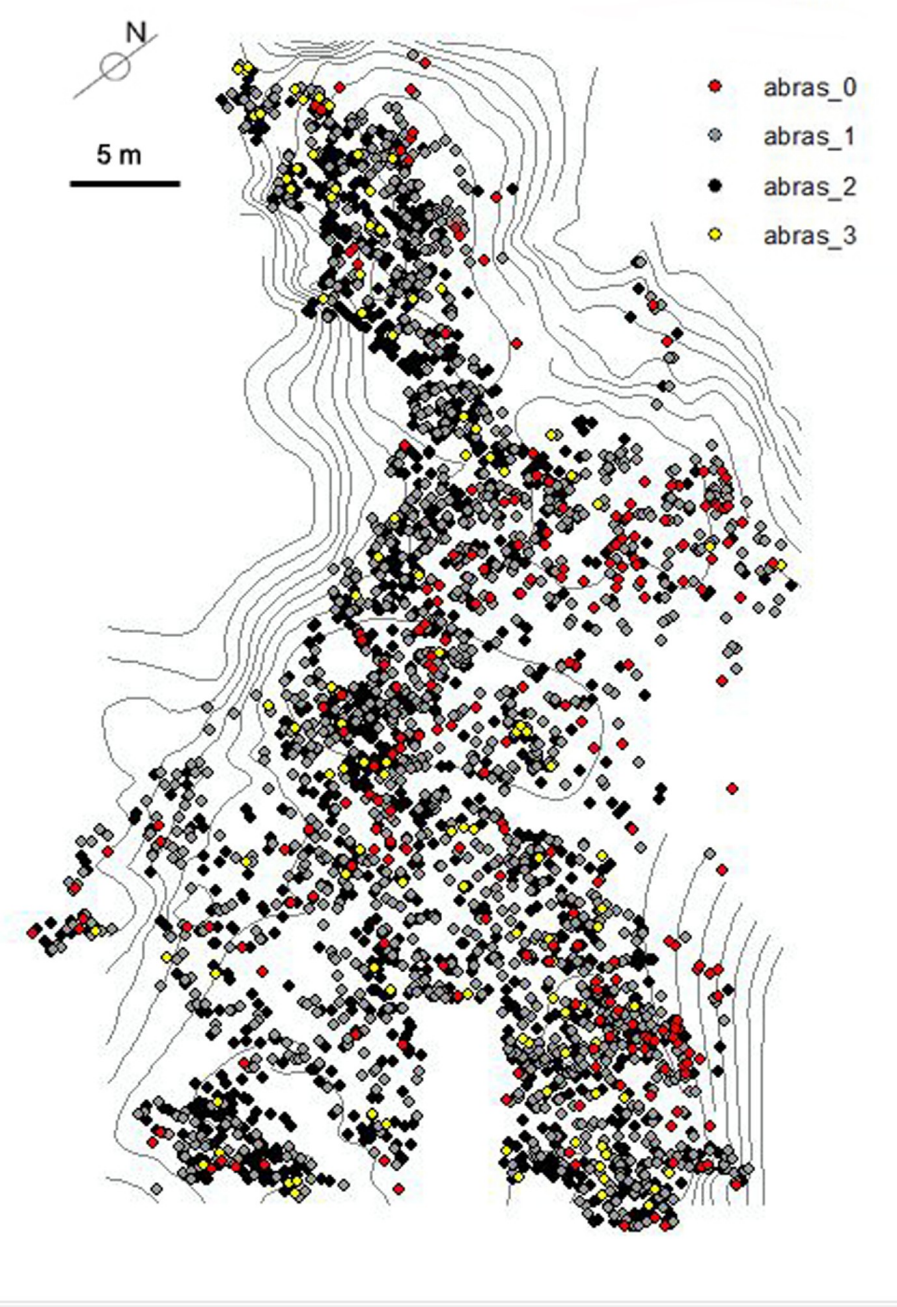
Plan of the castel di Guido excavation area with distribution of the faunal remains and contour map of the channel. Stages of abrasion are the same as those used in lithic analysis: 0 = fresh; 1 = slightly abraded; 2 = abraded; 3 = very abraded. The total N of specimens is 4037. Contour lines are at 20 cm. intervals. From [20: Fig 48].

**Fig 3:**
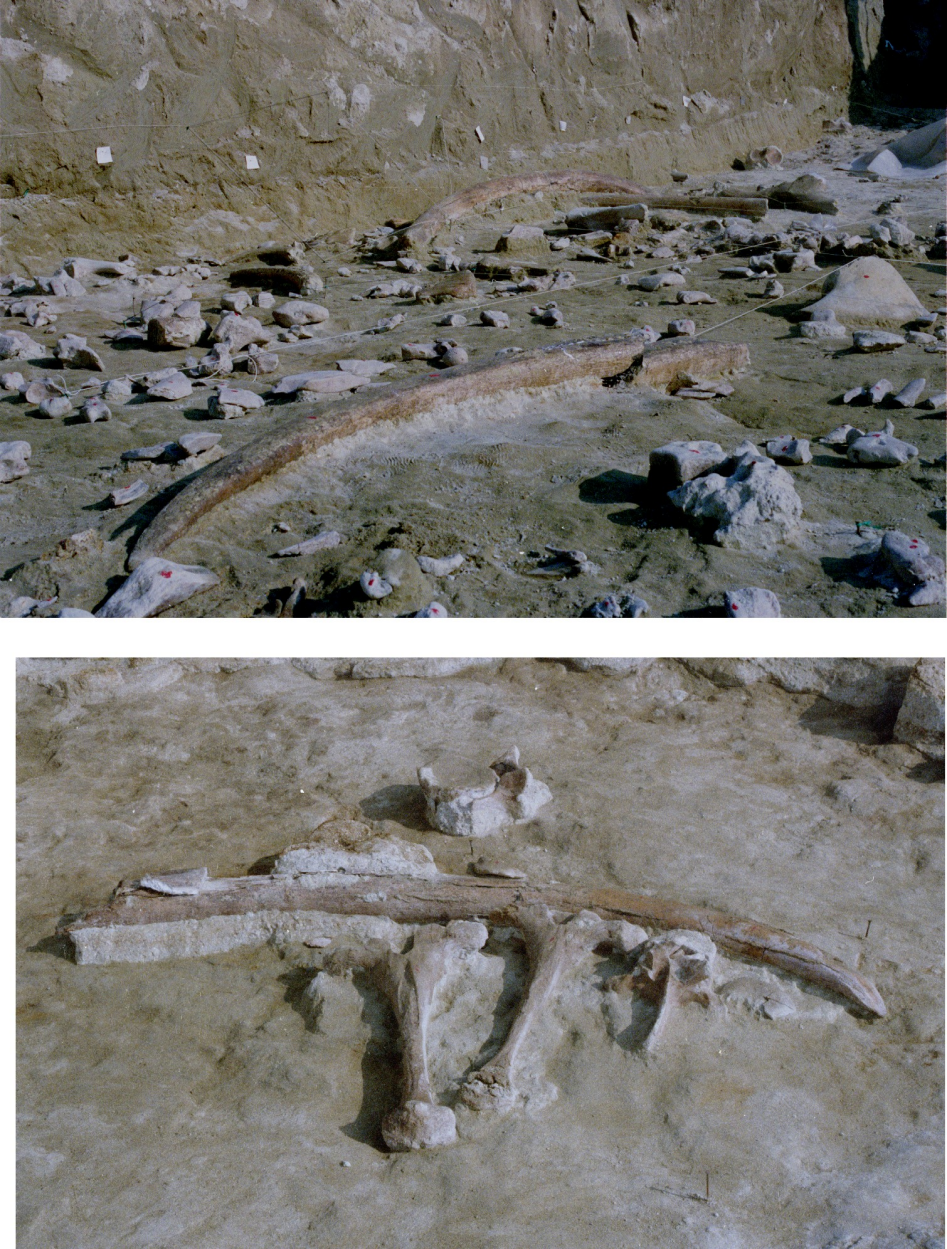
CdG excavation photo. Top: note the tusks and small objects at slightly different elevation. Bottom: two elephant neural spines and a vertebra of a smaller animal washed up against a tusk.

**Fig 4:**
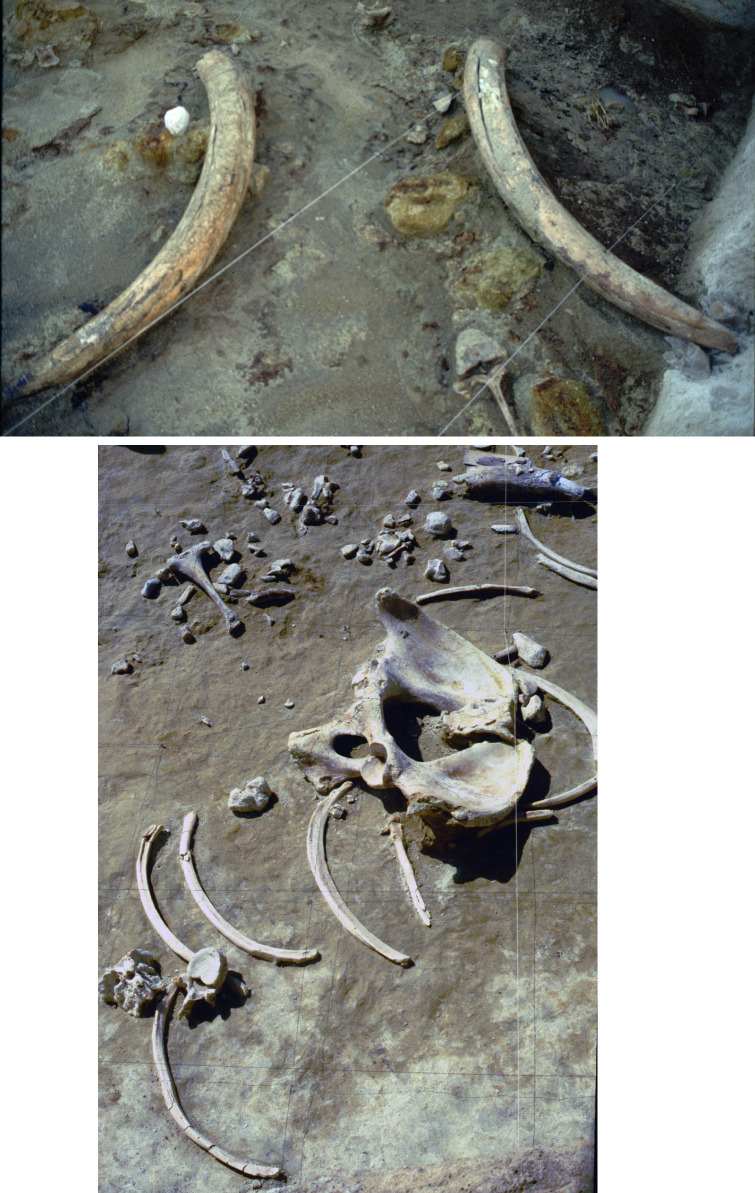
CdG excavation photo. Some elements were in anatomical association. Top: two tusks clearly belonging to the same animal. Bottom: elephant pelvis with the two hip bones in association.

**Fig 5:**
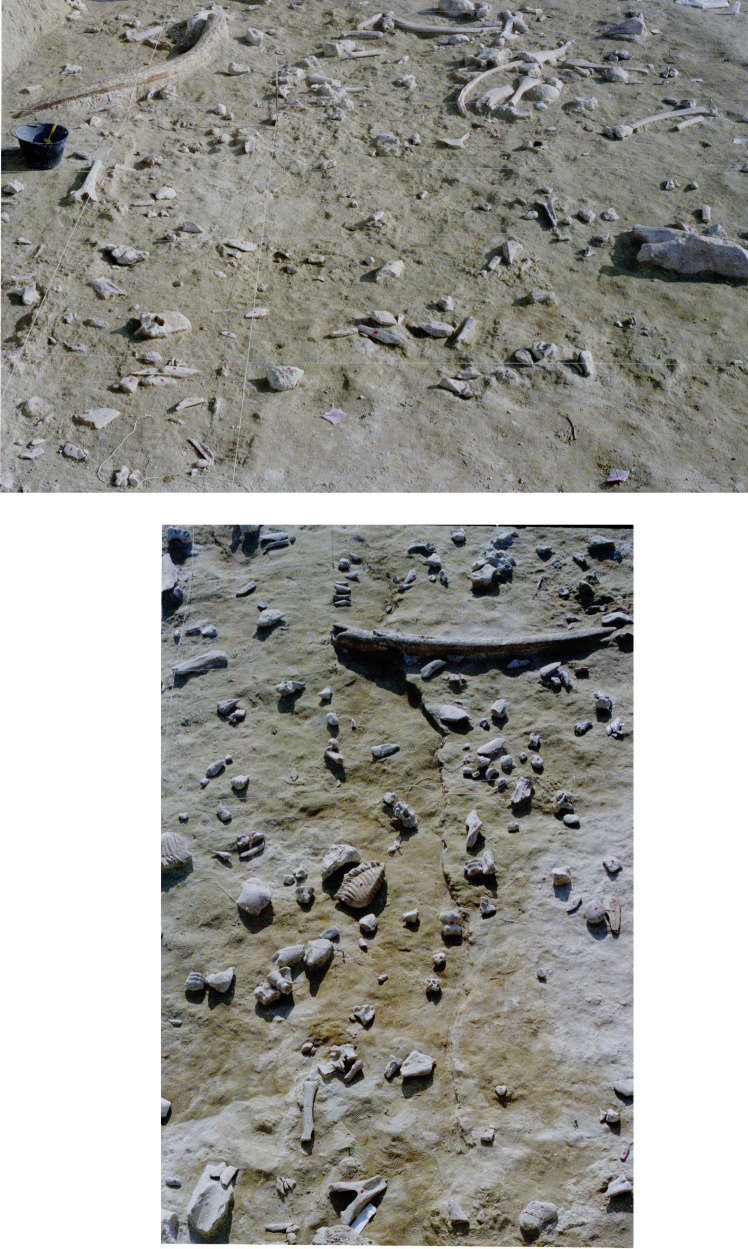
CdG excavation photo. Dispersed elements. The microfault (bottom image) is probably due to postdepositional sediment pressure or a small earthquake.

Castel di Guido (CdG) is an open-air Acheulian site about 20 km WNW of Rome, on the southern side of the Monti Sabatini volcanic complex. It is 9 km from the present-day Tyrrhenian Sea coast and 73 m a.s.l. It was excavated between 1979 and 1991 on an area of 1100 sq m [18]. The excavations produced a large number of faunal remains (S2, S3 Tables in S1 File) and lithic artifacts (all small and large tools plus cores, percussors and 20 unretouched flakes = 266; S5 Table in S1 File). These materials were found on the bottom of an erosion surface representing the remains of a gully shaped by an ephemeral stream (S1 Fig in S1 File). The bottom is 23–25 m wide, with roughly parallel banks. The remains appeared distributed on a single surface and embedded in a sandy layer 0 to 30 cm thick but geological and taphonomic studies [19–22] suggest that the site is a palimpsest of several episodes of human use with partial reworking, some removal of the fine fraction and association on the same surface of remains belonging to separate episodes of use. A process of continuous erosion by water flow “compressed” the layer so objects of different chronology but culturally homogeneous were lain side by side. This explains variability in surface preservation (Fig 2, S3, S4 Tables in S1 File), the high number of natural striations on bones from coarse and fine grains in the sandy water flow and the dispersal of small elements with only few large bones in anatomical association (Figs 2–5).

The CdG bone tool assemblage is the largest of all Middle Pleistocene sites that have yielded artifacts made by flaking elephant long bone diaphyses. It is known from a monograph published in 1996 [18] yet it is still poorly understood for a number of reasons.

First of all, many of the drawings published in the monograph are incorrect, a few are definitely wrong and the artifact state of preservation is overlooked. The drawings represent all artifacts as fresh and well-preserved, which is definitely not the case (S2, S3 Figs in S1 File). According to the monograph there are 373 bone tools including 99 bifaces. Misunderstanding of flaking features, limited technological and taphonomic analyses and overestimate of true bone tools by inclusion of pieces with a doubtful amount of human-made modifications led to imprecise and at times optimistic interpretations (S4 Fig in S1 File [23]). Lower quantities of bone bifaces were suggested by [24] but the total number of true artifacts remained unclear.

The lack of taphonomic analyses of the bone tools was the basis for skepticism expressed by Gary Haynes [25] regarding the interpretations of Acheulian bone bifaces and other bone tools as true artifacts. According to him, Castel di Guido and other sites that contained fragments of elephant bones flaked into a hand-axe shape or retouched along the edges had been affected by fluvial processes or carnivore gnawing and various natural processes of bone breakage. He argued that the specimen morphologies were not necessarily a reflection of human action and rejected their interpretation as unproven. He called for analyses of complete assemblages from a taphonomic viewpoint and a quantified and explicit description of features that define human action, as opposed to natural agencies. This is what we have done and we report in this paper. We have included in the study a small set of bone tools from Malagrotta (S5 and S11 Figs in S1 File).

## Dating

The site is currently inaccessible because the excavation area was refilled with sediment and the whole surrounding area was planted with trees by the landowning company after the end of the excavations. However, the original stratigraphic investigations performed during the excavation have been integrated with new field surveys at several geologic sections cropping out in the area which included Castel di Guido (CdG) and Malagrotta (MG). The age of the two sites has been reassessed thanks to four new samples dated by the ^40^Ar/^39^Ar on single sanidine crystals (Table 2; S17 Fig in S2 File). Two of these samples (CdG-1 and CdG-2) were collected during the original excavations and stored in the Biology Department of the University of Pisa. Sample MG-1 consisted of pyroclastic material cemented on a fossil bone fragment found during excavation, also stored in the Biology Department of the University of Pisa (S5 Fig in S1 File). MG-2 is a new sample collected from a geologic section located ca. 200 m from the archaeological site described by [26], a paper by Caloi and Palombo. They attribute the outcrop in which sample MG-2 was collected to the same stratigraphic sequence exposed at the Malagrotta archaeological site, as discussed in [13].

**Table 2:** ^40^Ar/^39^Ar dates.

Sample name	Latitude, Longitude	Age* (ka ± 2ϭ**)	N of crystals	*MSWD*	*P value*	Inv. Isochron age* (ka ± 2ϭ**)	*MSWD*	^40^Ar/^39^Ar_*Init*_. ± 2ϭ
**CDG-1**	41° 54’ 00.18" N	395 ± 3	3 out of 15	2.5	0.1	395 ±4	4.8	295 ± 38
	12° 16’ 39.53" E							
**CDG-2**	41° 54’ 00.18" N	465 ± 5	7 out of 9	0.7	0.6	465 ±5	0.8	301 ± 8
	12° 16’ 39.53" E							
**MG-1**	41° 53’ 03.74" N	378 ± 6	2 out of 14	0.4	0.5	N/A	N/A	N/A
	12° 19’ 01.05" E							
**MG-2**	41° 53’ 01.96" N	451 ± 2	10 out of 11	1.5	0.1	451 ±2	1.6	291 ± 18
	12° 19’ 06.30" E							

* Age recalculated at 2ϭ analytical uncertainties according to the optimized calibration of the standard [14, 15]. MSWD = Mean Square Weighted Deviation. See Geochronological and stratigraphic data in Supporting Information.

**Fig 6:**
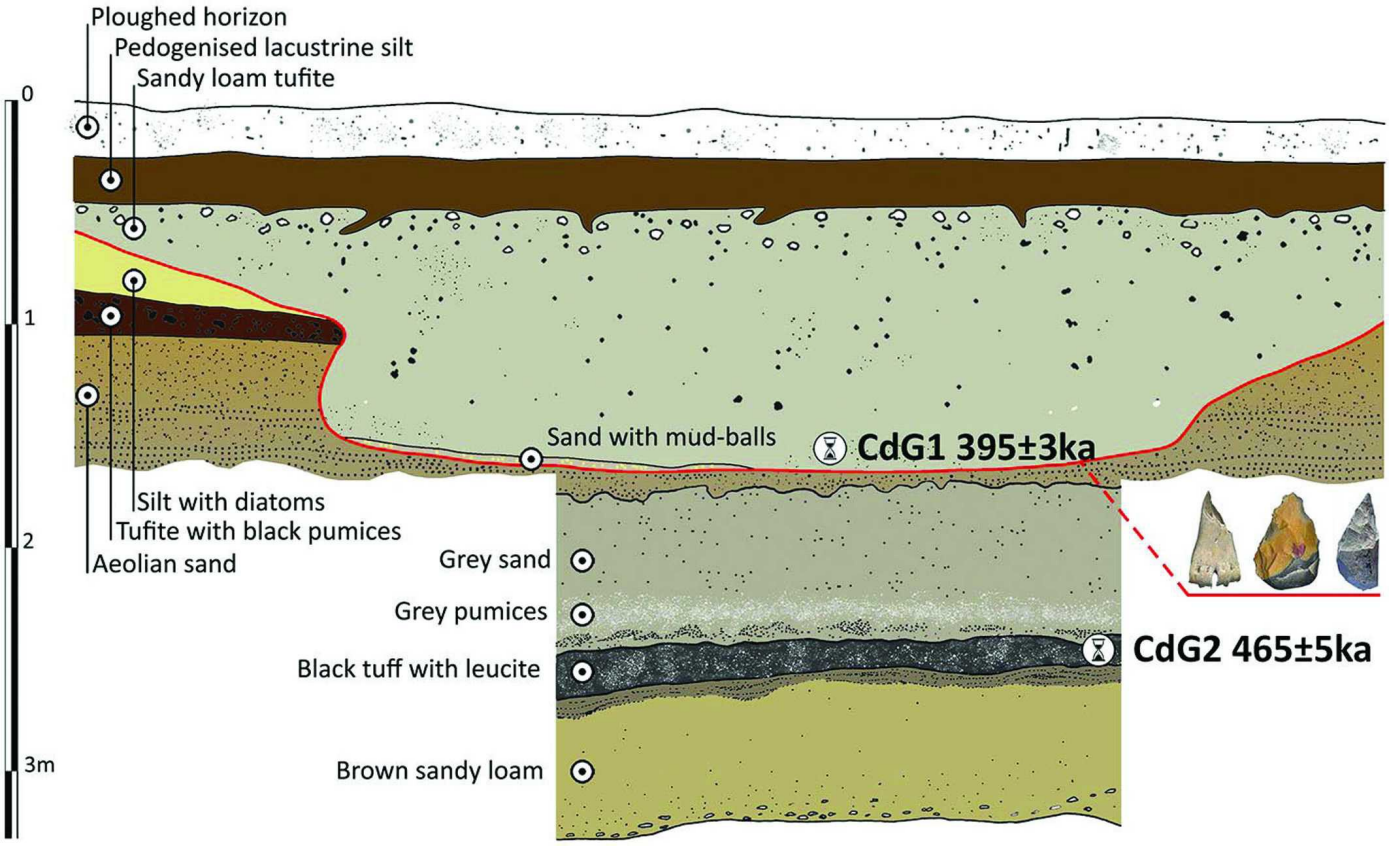
Excavation profile with location of the two dated samples CdG1 and CdG2. The red line indicates the paleosurface. Drawing by G. Boschian.

Sample CdG-1 was collected in a volcaniclastic deposit (“tufite”) above the paleosurface with artifacts and faunal remains. CdG-2 was collected in a primary pyroclastic deposit occurring ca.1 m below the archaeological level (Fig 6).

The weighted mean age of CdG-1 is 395 ± 3 ka, calculated from the weighted mean of the three youngest crystals out of 15 dated (Table 2) because no crystals belonging to the youngest eruption of the area were found within the sample. The probability diagram (S18 Fig in S2 File) is multimodal, indicating that this volcaniclastic deposit is reworked. The age of the youngest crystals provides the date before which the archaeological level was deposited. Sample CdG-2 yielded an age of 465 ± 5 ka, based on the weighted mean of seven of nine individually dated crystals. Thus the paleosurface is bracketed between 465 and 395 ka. Its stratigraphic position close to the 395 ka sample suggests an age of ~400 ka or not much older. The Malagrotta lithic and bone industry is bracketed by the ages of samples MG-1 and MG-2, between 451 and 378 ka (details in Supporting Information).

## Materials and methods

### Repositories

The large number of previously identified bone tools and possible bone tools, all the lithic materials of Castel di Guido and the small set of bone tools from Malagrotta are stored in the laboratory of Giovanni Boschian in the Department of Biology, University of Pisa. Because the materials had been moved years before from one main repository (the Department of Archaeological Sciences) to two different locations (the laboratory of Giovanni Boschian in the Department of Biology and the storage area of the University of Pisa in Montacchiello, a small town to the periphery of Pisa) there had been a certain amount of mixing and loss of information. Thus sorting and analyzing these materials took a good deal of time. Fortunately practically all bone pieces and all lithic pieces have a catalogue number. The fauna and the natural pebbles and cobbles are stored in Montacchiello.

### Sampling and sorting

Four different data bases on the CdG bone artifacts had been made before our study: 1. The list and description of all identified bone artifacts (n = 373) in the Radmilli and Boschian monograph on CdG [18]. 2. The work of Daniela Saccà [24] which classified bone tools (N = 79 including 4 on bovid bones) using three main categories (bifacially or unifacially flaked, pieces with limited modification and intermediate pieces (= wedges). 3. A list of bone tools (N = 71) done by a student (Alex Bertacchi) [27] who followed Saccà’s classification. 4. A more complete database (N = 204) made by Giovanni Boschian, including bone and stone artifacts illustrated by many photos.

We excluded from lithic analysis flake fragments, chunks, and natural pieces. After checking the database of Giovanni Boschian and the catalogue in the 1996 monograph we also excluded from analysis a fairly large number of bone and stone pieces (N = 88) for one or more of the following reasons: pieces too rolled, too altered or too damaged for correct identification, and various stone artifacts from the site of Malagrotta that had been mixed with those of Castel di Guido by various researchers.

For lithics we used a very simplified version of Bordes’ typology and classification, which is based on primary types. Our categories do not cross-cut Bordes’ types, they just lump them together. In the large tool categories one type “pointed tools”, which does not exist in Bordes’ typology, is similar to the same bone tool category by the shaping of only the functional part. Taphonomic attributes and attributes related to the mode of production are coded in the Excel file (see list of variables below). Pieces with marginal or partial retouch are classified as “Retouched pieces”.

### Nature of the bone sample

The comprehensive nature of the sample chosen for study is important because the study of early bone tools has often been based on the analysis of a few pieces picked from a larger assemblage of faunal remains. We avoided this pitfall thanks to work by Daniela Saccà who set aside a large number of broken elephant bones that were not considered artifacts (150 diaphysis fragments and other kinds of bone fragments) but showed fractures suggestive of human modification. Detailed analysis of this subsample led to the extraction of a few pieces added to our sample.

All the analyzed materials were bagged in individual Minigrip bags and labelled with pre-printed labels.

### Bone artifact analysis

The bone tools from Castel di Guido and Malagrotta do not lend themselves to the microscopic study of utilization traces. They come from open-air sites and were affected by sandy water abrasion (S4 Table in S1 File). To sort minimally modified bone artifacts from the by-products of marrow extraction, carnivore-gnawed bones or natural processes of bone breakage, and to recognize intentional modification for the purpose of shaping the artifacts, our approach must be based on the technology of the whole assemblage. Criteria for the identification of deliberately modified bone artifacts must be based on the recognition of the classic attributes of percussion flaking as expressed in the morphologically similar stone bifaces, partial bifaces and unifaces of Castel di Guido [28].

We used the following criteria: a) Regular shape of the worked edge in plan form and profile. b) Large and deep flaking scars with clear origin or bulb of percussion. c) Oblique angle of edge flaking. d) Invasive continuous removals. e) No nibbling or notching of the edges; hyenas’ gnawing will not produce scars bigger than 15 mm [29].

Extensive bifacial flaking and bilateral symmetry are highly diagnostic and some of these occur at Castel di Guido. Essentially we used criteria similar to those used in sorting stone artifacts from broken rocks [30, 31].

Taphonomic analysis of all the faunal remains is an important prerequisite of this paper. This work has been carried out by Daniela Saccà in her thesis [20] and papers [22, 24, 32]. Her work provides a comprehensive coverage of the context, the taxonomical composition of the assemblage, the state of preservation and the state of fragmentation of all the fauna. The total number of faunal remains is 3245 NISP (Number of Identified Specimens) of which 1381 are elephant (*Palaeoloxodon antiquus*) specimens with a MNI (Minimum Number of Individuals) of 11. NISP of aurochs (*Bos primigenius*) is 1399 with a MNI of 43 (S2 Table in S1 File). The absolute majority of bone tools are made on elephant diaphysis and only few intentionally flaked and shaped pieces were made on aurochs bones.

For the analysis of the presumed bone artifacts we built an Excel file (for a total of 299 specimens) recording the following main variables:

- Species or animal size (almost all of *Palaeoloxodon antiquus* size, only a few pieces were of *Bos* or bovid size).
- Blank element (e.g. diaphysis fragment, flake, compact bone, bovid radius, rib)
- Length, breadth and thickness (in mm)
- Taphonomic attributes. These are the same as those used for lithic analysis: fresh, slightly abraded, abraded, very abraded. Differences between state of abrasion of faces or edges are noted in a separate column.
- Shape of base and of distal edge
- Implement broken, complete or almost complete
- Type. This is the final classification grouping artifacts in accordance with their common characters and based on recognition of technological attributes.
- Index of confidence, using the following categories: 0 = unmodified or too abraded for a diagnosis; 1 = possible tool but no sufficient evidence, with unpatterned removals (likely due to natural damage) or removals too damaged to be identified with certainty; 2 = good evidence of intentionally flaked artifact; 3 = clear evidence supported by a combination of attributes.

## Results

We consider true bone tools only pieces with index of confidence 2 or 3. Table 3 shows that the total number of verified human-made artifacts is 109. However, of these 11 are unretouched flakes which are undoubtedly true artifacts, most likely the result of shaping large tools but not intentionally modified pieces. Two of these flakes may have been retouched but they are too abraded to diagnose with confidence. Thus the verified tools are 98. **This is the highest number of flaked bone tools from any Early or Middle Pleistocene site published to date.**

**Table 3:** Index of confidence*.

Index of confidence	N
0	115
1	60
2	61
3	48
Total	284

*The total number of pieces in our Excel file is 299 but 15 specimens were excluded from these counts (teeth or tusk fragments or misplaced bone fragments).

We classified them as follows:

Only 27% of 109 bone tools (including unretouched flakes in counts) are fresh or only slightly abraded. Many more are abraded but even some of the very abraded can often be recognized and identified with certainty because removals are patterned.

Smoothers (= *lissoirs*, in French) are a common Upper Paleolithic tool made on ungulate ribs, longitudinally split to produce two thin half ribs. These half ribs are then shaped by grinding and scraping, with a rounded end polished by use, showing wear facets and striations. By their similarity to ethnographic bone tools used by the Sami people (Lapps, indigenous people of northern Europe) they are interpreted as tools for smoothing dry hides. They are frequent in Aurignacian assemblages; four come from two Mousterian of Acheulian Tradition sites in France [37, 38]. The Castel di Guido and Schöningen pieces with clearly smoothed and polished tips are very similar to the Aurignacian and Mousterian *lissoir*s and can, in our view, be classed as smoothers. The CdG *Bos* radius and its similarity to *lissoirs* were first noted by Saccà [24].

## Discussion

Blanks of tools are mainly elephant diaphysis fragments, more exactly 77 on 105 (four bovid specimens excluded) that is 73%. All these diaphysis fragments have a shaft circumference less than one-half of the original and a shaft length less than one half of the original, more commonly less than one-fourth [24, 39].

In the faunal assemblage [22] there are about 464 unmodified diaphysis fragments of this kind. High frequencies of oblique angles of bone fractures and curved or V-shaped fracture outline (more than 60%) [22, 39] indicate that the elephant long bones were broken when still fresh, prior to their covering by sediment and in the almost total absence of carnivore damage. Compared to the elephant MNI (N = 11; S2 Table in S1 File) and given the almost total absence of complete or almost complete long bones, the high number of diaphysis fragments indicates a very high degree of fragmentation, unusual in elephant long bones [40, 41]. Although small marrow cavities are present in elephant bones and fragmentation for marrow extraction is a plausible hypothesis [42], the archaeological examples suggest that the very high degree of fragmentation of elephant limb bones may have another goal. Clearly the elephant long bones were broken systematically to obtain blanks appropriate to shape a tool, essentially elongate, flat and roughly pointed pieces of bone (S7 Fig in S1 File) [31]. A high degree of fragmentation of elephant long bones is also reported from Olduvai but the number of verified tools on elephant bone is only 19, and not many are on shaft fragments [3: Table 14].

With the exception of intermediate pieces and one smoother (the *Bos* radius with a smoothed tip, Fig 14) all the Castel di Guido bone tools were intentionally shaped by free hand direct percussion, like the stone tools. This is also true of most other Middle Pleistocene bone tools from Italy and other countries in Western Europe. However, the 88 bone tools of Schöningen 13-II-4 (the Spear Horizon, now dated to MIS 9 [43] hence quite younger than Castel di Guido) were mostly long bone and metapodial fragments used as retouchers, hammers and anvils, for percussive and knapping activities [16]. These tool types are not present at Castel di Guido. The bone tools of Schöningen 12 II-4, also dated to MIS 9 [17], consist of retouchers and percussors but also include a few smoothed-tip (i.e. smoothers or *lissoir*s) with the best example illustrated in [17: Fig 12] very similar to the smoother from Castel di Guido (Fig 14).

A significant aspect of the Castel di Guido bone tool assemblage is the **diversity of tool types.** Differently from the Olduvai elephant bone tools [3] and those listed in S1 Table in S1 File, the CdG bone tools are neither expedient nor occasional. The types listed in Table 4 are pieces that, with few exceptions, can be easily classified because their morphological and technological characters are stable and repetitive, with distinct flaking patterns.

**Table 4:** Castel di Guido bone tool specimens with index of confidence 2 or 3.

Type	N	%	Figures
Bifaces	8	7.3	Fig 7
Partial bifaces*	9	8.2	Fig 8
Partial biface-knives	4	3.7	Fig 9
Pointed tools	39	35.8	Figs 10 and 12
Intermediate tools (also called wedges)	16	14.7	Fig 11
Unifaces	6	5.5	Fig 12
Core-chopper, tool with multiple edges	2	1.8	Fig 13
Smoother (= *lissoir*; *Bos* radius with polished tip)	1	0.9	Fig 14
Retouched flakes, small tools	9	8.3	Fig 15
Broken tool, tool fragment	4	3.7	Fig 15
Unretouched flakes	11	10.1	S6 Fig in S1 File
Total	109	100	

*Partial bifaces are pieces with only limited bifacial retouch on one face [33].

**Fig 7:**
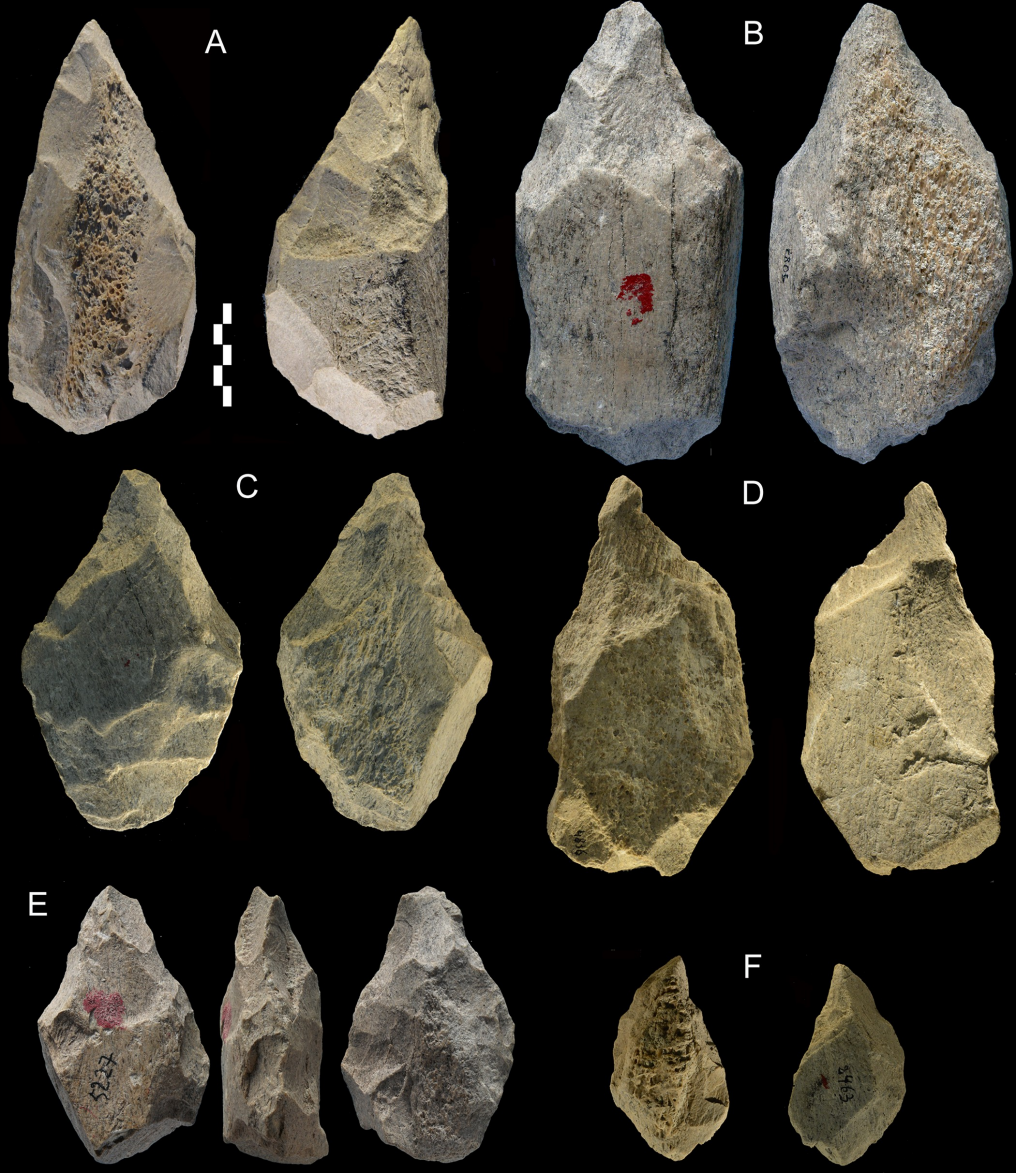
Castel di Guido, bifaces made on diaphysis fragments. Catalogue numbers (following the letter order) 1014, 5087, 5089, 4836, 5227, 2463. A and F are only slightly abraded, B-E have abraded and fresh removals on the same face, suggesting reuse (S4 Table in S1 File). Scale bar 5 cm.

**Fig 8:**
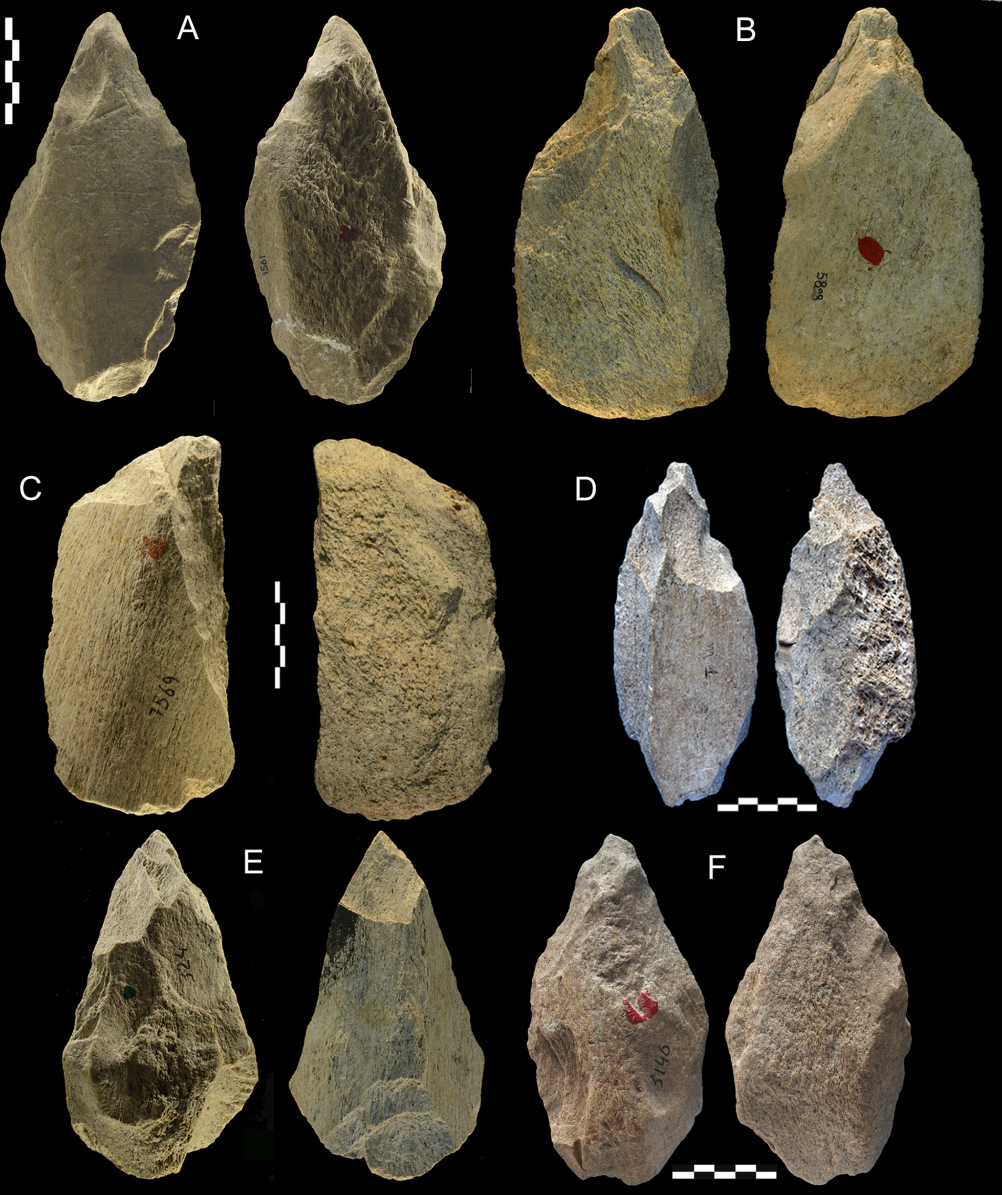
Castel di Guido. (A) biface; (B-F) partial bifaces. Catalogue numbers 1561, 5898, 7569, TM 10433, 324, 5146. Note that the partial bifaces B and E are abraded on one face and fresh or only slightly abraded on the other. This suggests that some large pieces were partly buried in sand and the exposed face (sometimes- but not always- indicated by a red or green dot assigned at excavation) was abraded in situ by the flow of sandy water. See similar occurrences in layer *m* of Torre in Pietra [34: p. 18]. Scale bar 5 cm.

**Fig 9:**
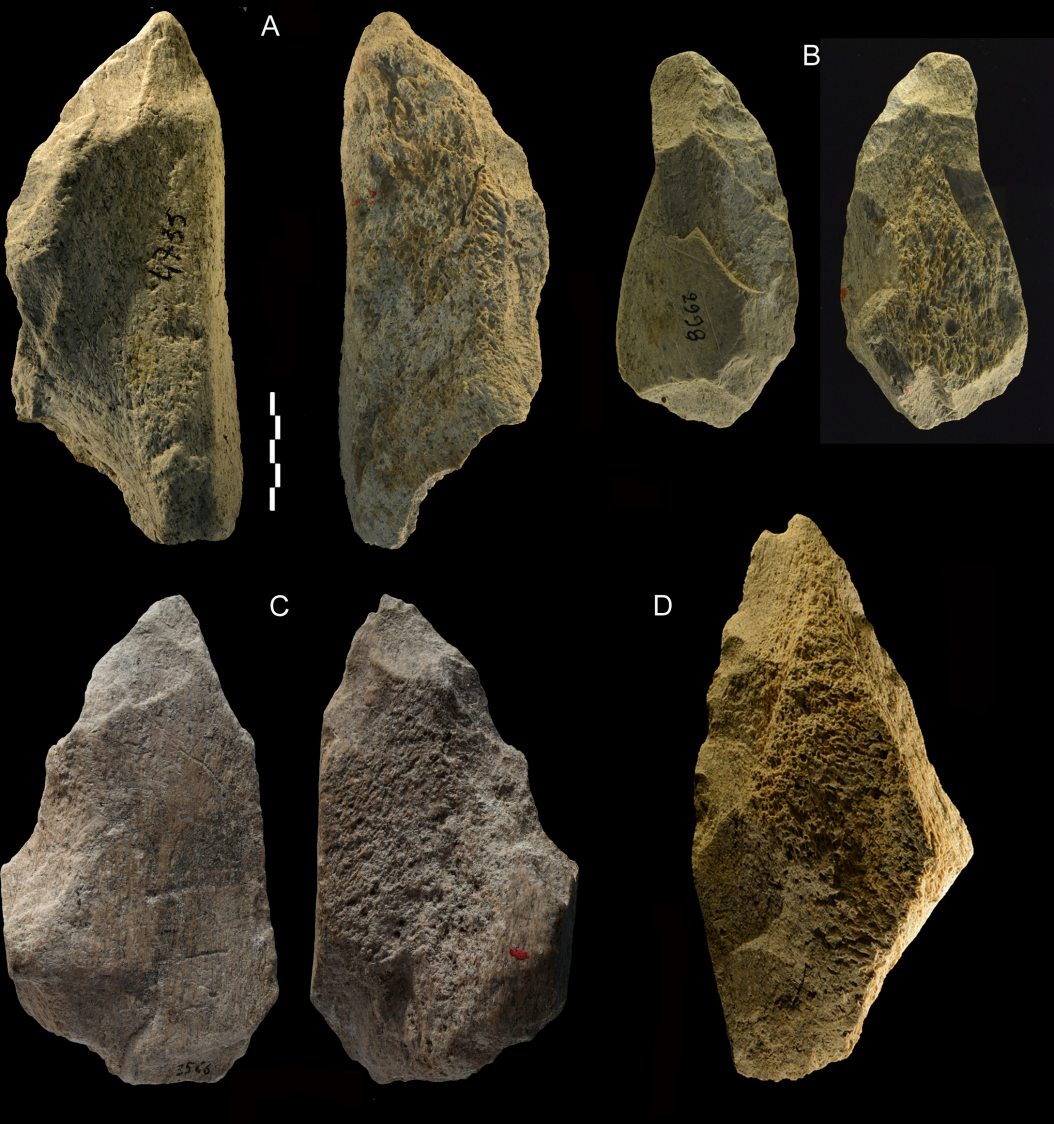
Castel di Guido. Partial bifaces-knives. (A-C) are asymmetrical large tools bifacially flaked on one side with a steep opposite side, either natural (as on these pieces) or blunted by retouch. This type of tools is found in the Middle Pleistocene Acheulian stone tools of East Africa where they are called “knives” [35, 36]. To our knowledge, these forms were not previously documented in the European Acheulian. (D) is unifacially flaked with a natural steep back. Catalogue numbers 4735, 2998, 3566, x281. Scale bar 5 cm.

**Fig 10:**
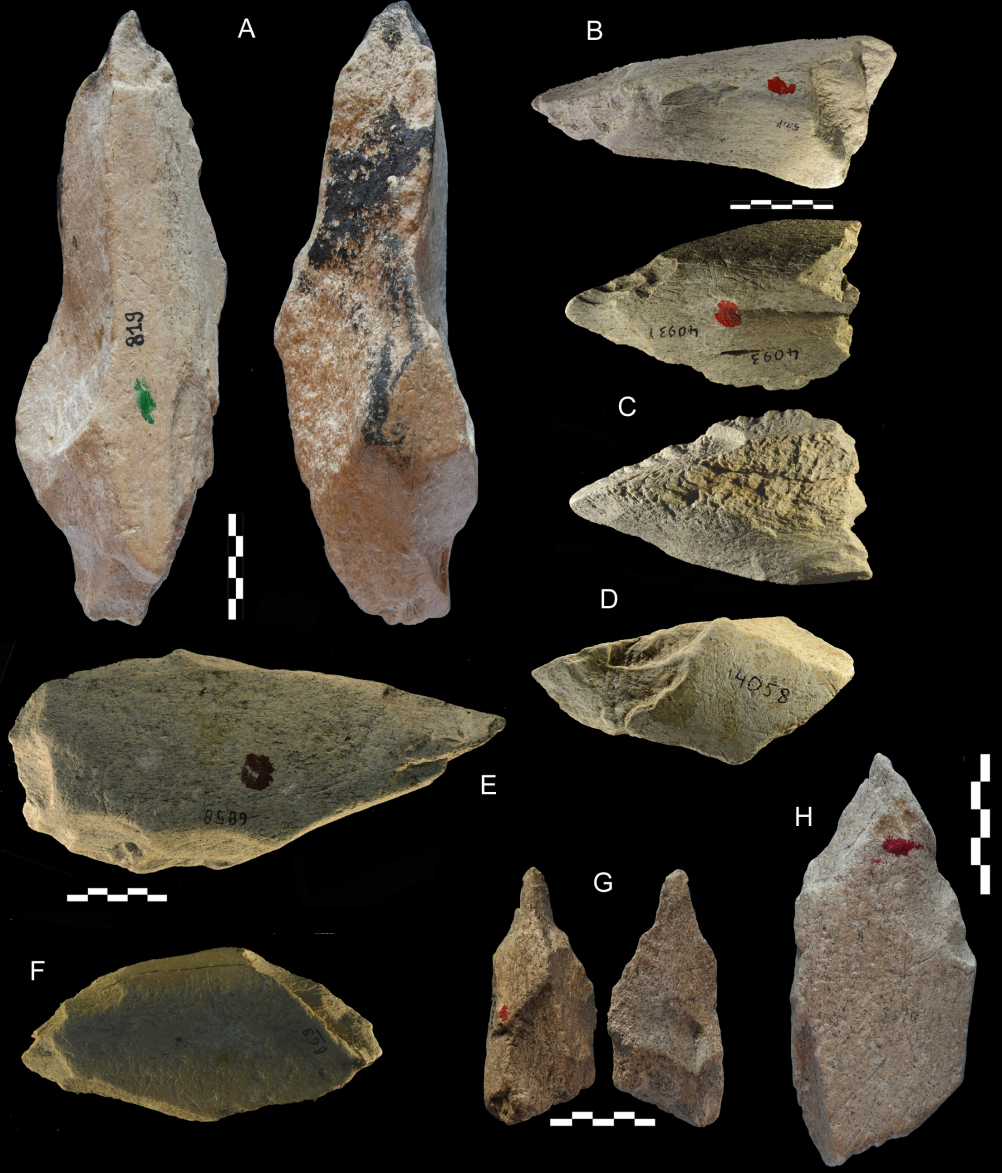
Castel di Guido. Pointed tools, abraded or slightly abraded (A-H). Catalogue numbers 819, 4093, 5817, 4058, 6858, 869, 3508, 2370. B and C can be classed as pointed wedges.

**Fig 11:**
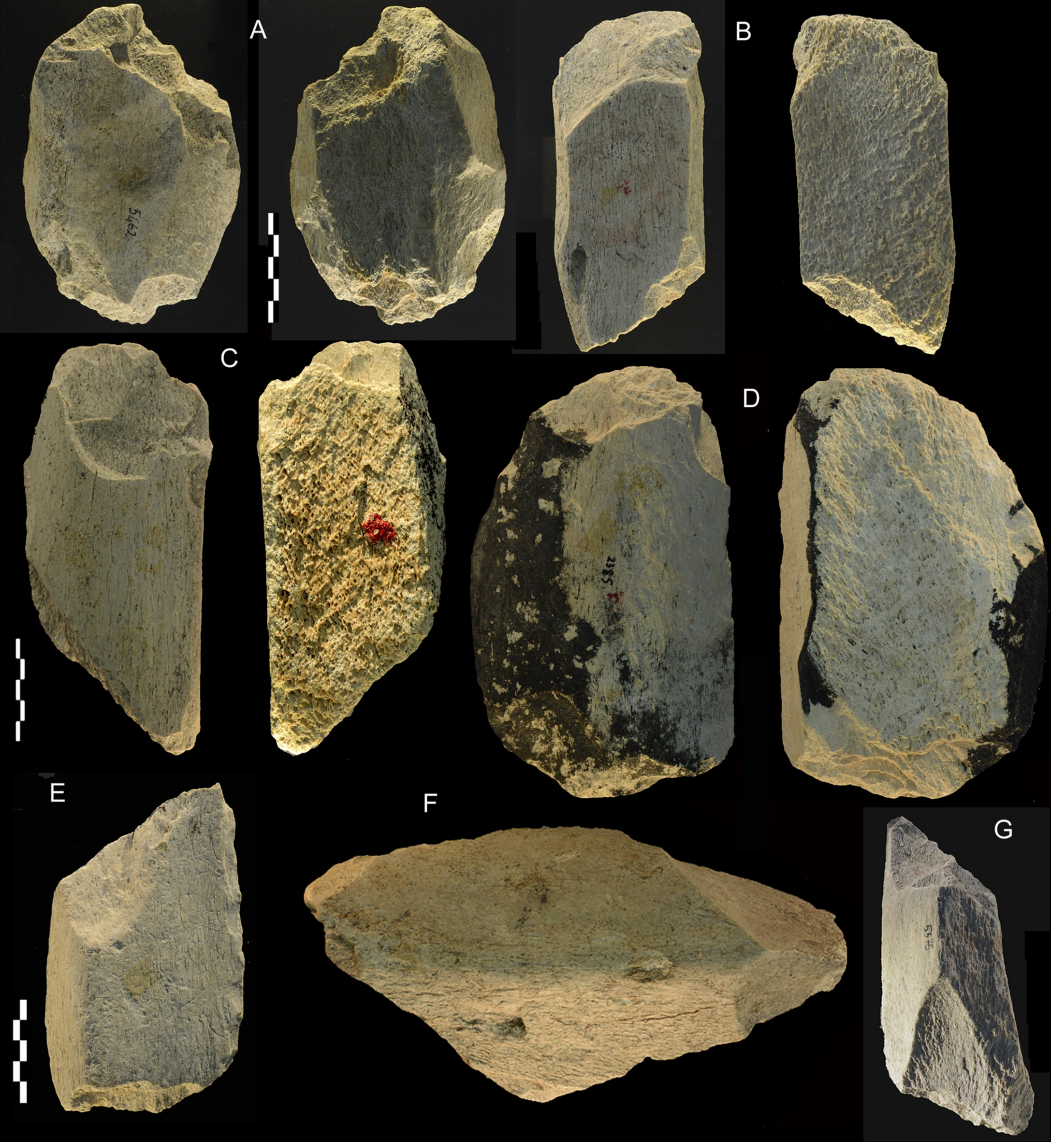
Castel di Guido. Intermediate tools (also called wedges). These tools, made on diaphysis fragments, show battering and crushing on the proximal end with some removal scars; the distal end has a broadly convex shape (A-D) or in some cases an end with a pointed morphology (E-G). They might have been used to work wood or to split bone. Catalogue numbers 5462, 4752, 5439, 2385,1294, 5500, 5575. Scale bar 5 cm.

**Fig 12:**
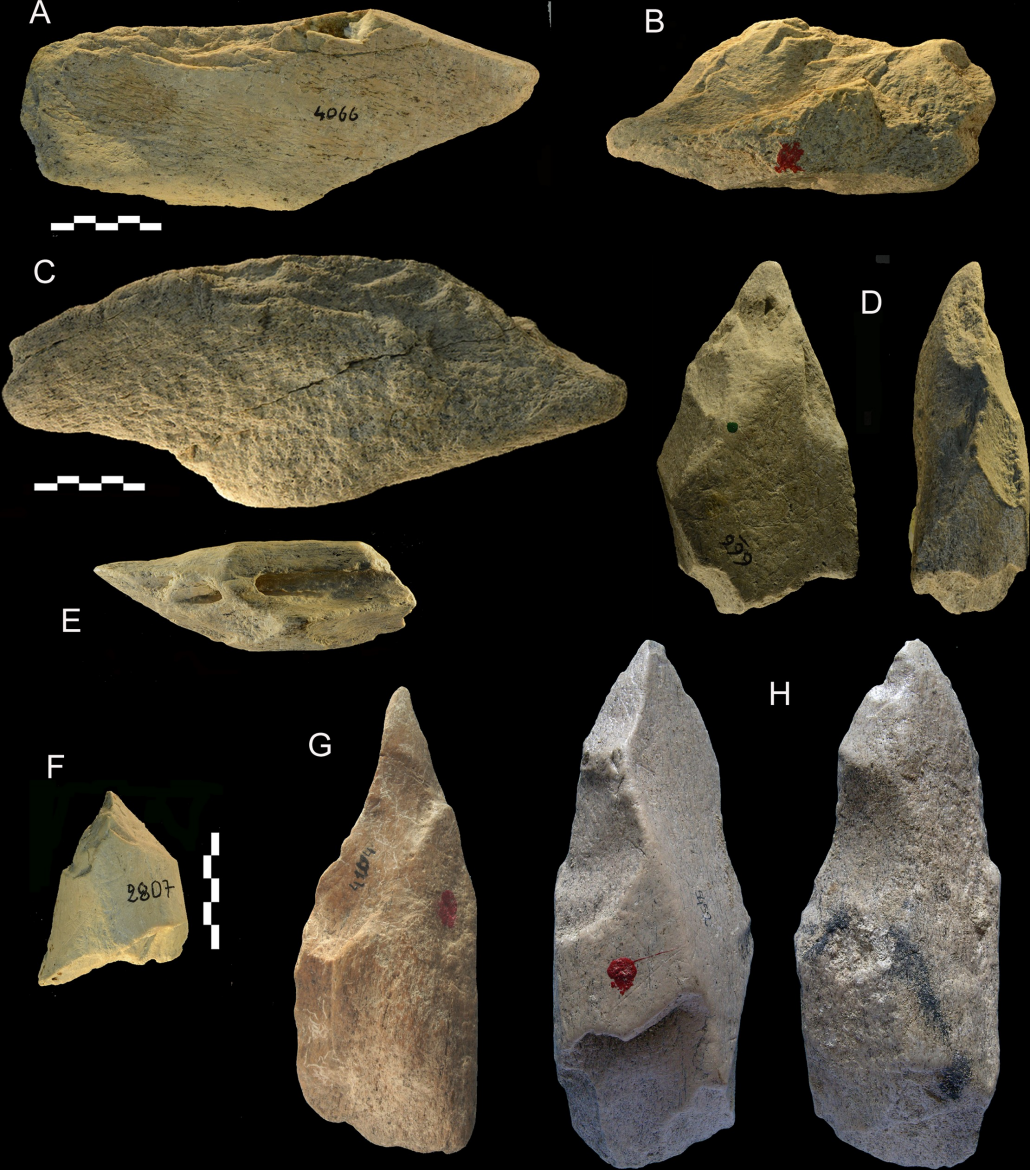
Castel di Guido, unifaces and pointed tools. (A-C) unifaces with a side scraper edge, catalogue numbers 4066, 6164, 4307. (D) pointed wedge (base is battered) catalogue number 666. (E-F) pointed tools on bovid diaphysis fragments, catalogue numbers 326, 2807. (G-H) pointed tools, catalogue numbers 4104, 5452.

**Fig 13:**
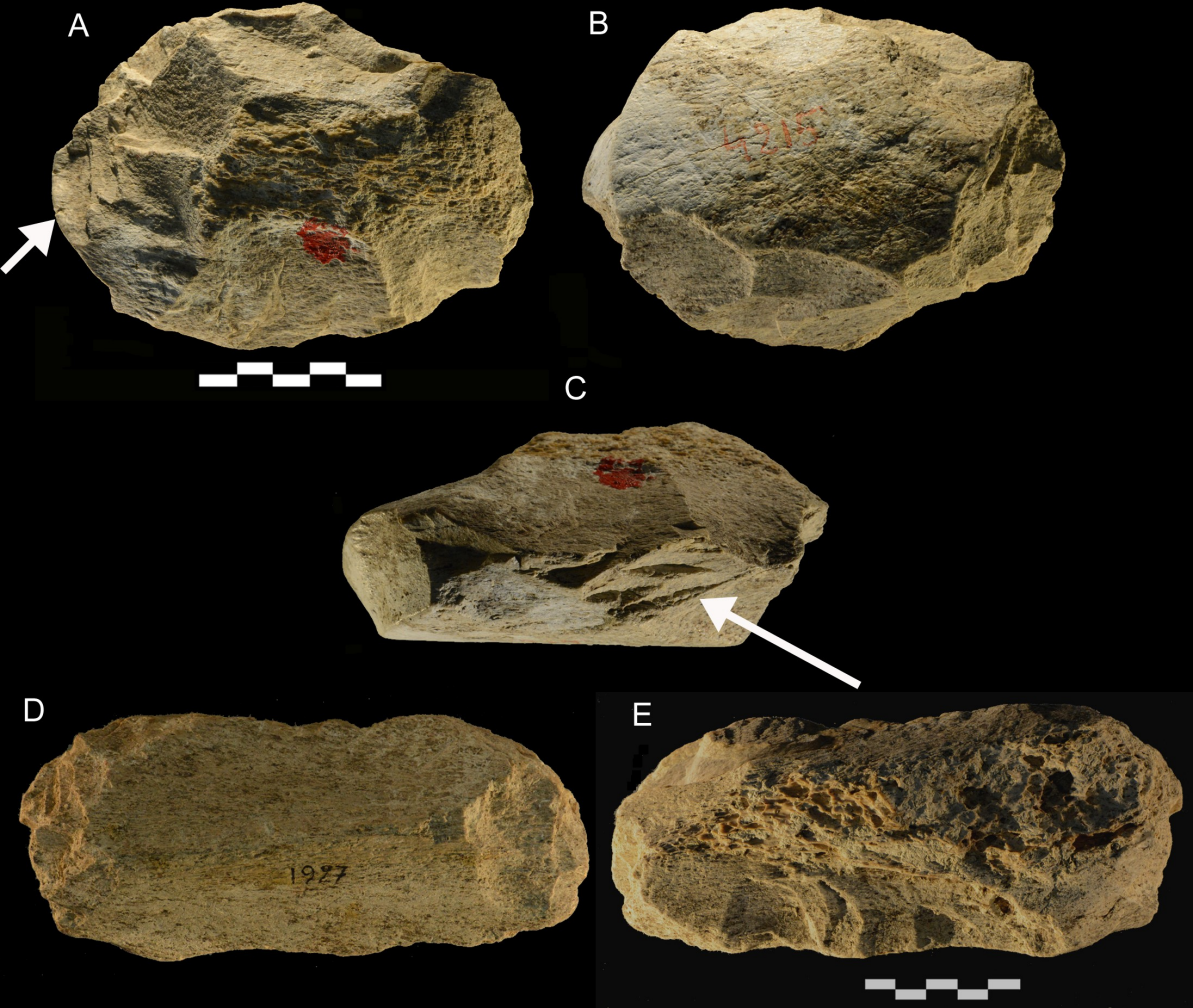
Castel di Guido. (A-C) Centripetal core with one heavily battered edge (arrow in C) and one edge (arrow in A) rounded and polished. (D, E) Intermediate piece with a lateral scraper edge. Both could be classed as tools with multiple uses.

**Fig 14:**
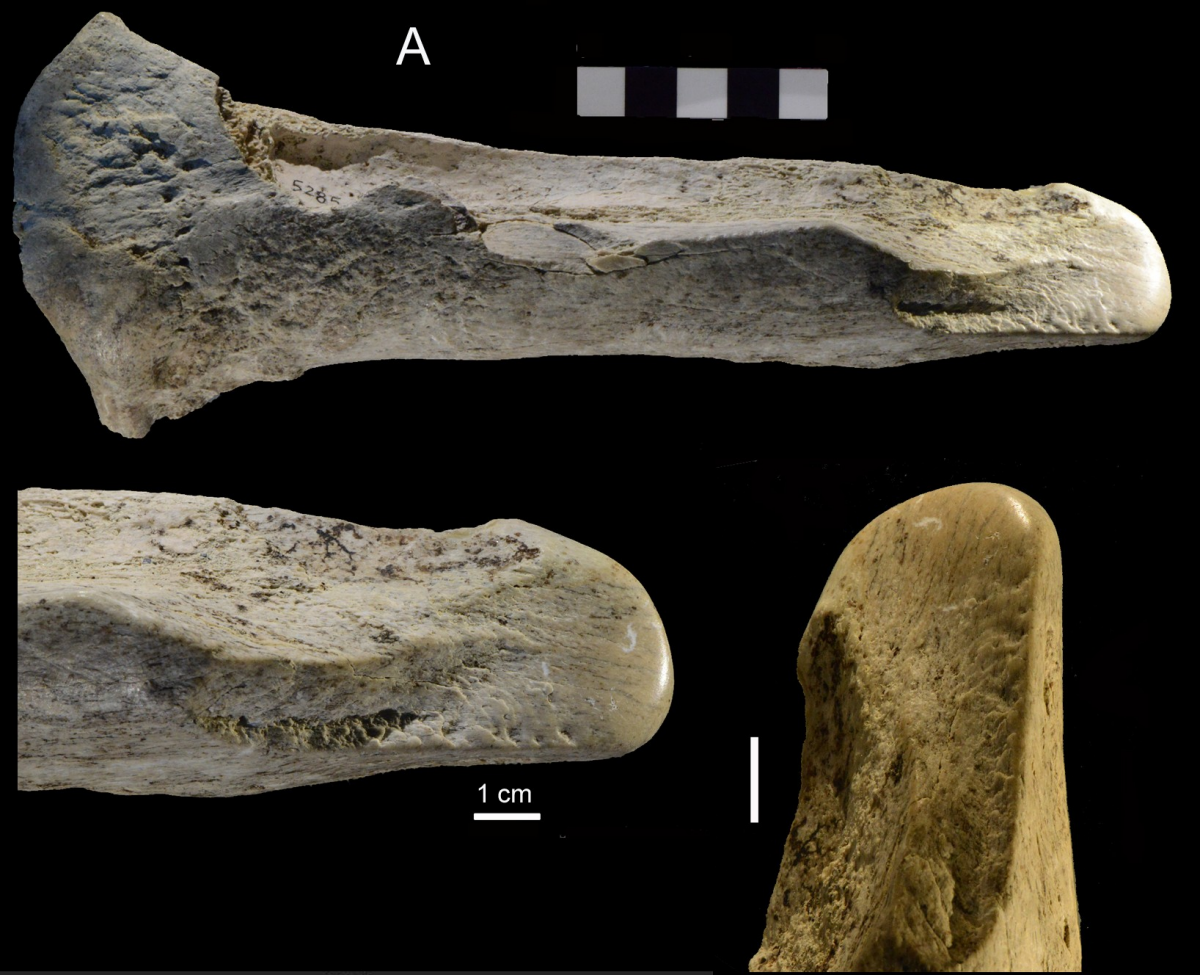
Castel di Guido, smoother. Right radius of *Bos primigenius* with polished tip, catalogue number 5285. Compare to Schöningen 12 II-4c, equid radius with polished tip [17].

**Fig 15:**
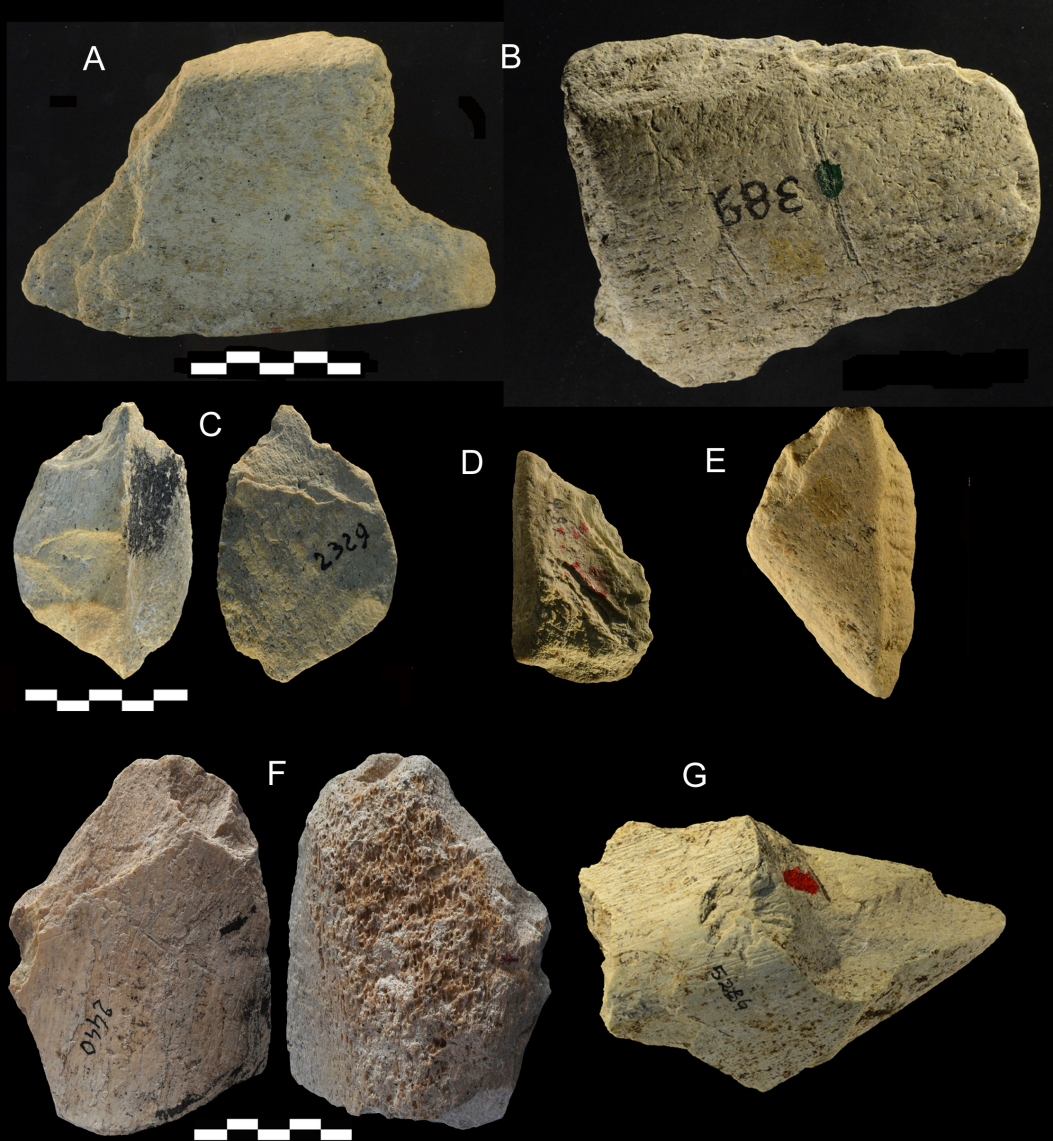
Castel di Guido. Retouched flakes (A, B, E), small tools on flakes (C, D) and broken tools (F, G). C is a *bec* on flake, D is a denticulate on flake. F has a stepped fracture at its base. The base of G is an irregular fracture and a percussion point in the center, probable cause of the break. This is most likely an intentional break. Catalogue numbers 765, 389, 2329, 4482, 1396, 2440, 5286. Scale 5 cm.

The smoother (Fig 14) and the same tool from Schöningen 12 II-4c1 can be considered as forerunners of similar tools found in Aurignacian assemblages [37]. They are common in Aurignacian assemblages; 117 *lissoirs* are reported from three Aurignacian sites in Southwest France (Castanet, Brassempouy and Gatzarria). The Castel di Guido and Schöningen pieces are on different kinds of blanks (radius of bovid or equid) but have very clearly smoothed and polished tips. They are similar to the later Aurignacian and Mousterian *lissoirs* but are much older and prove that some features of the innovative technologies of the Aurignacian have roots that go back to the Lower Paleolithic.

Of particular significance are the **intermediate tools,** also called wedges, made on diaphysis fragments (Fig 11) [24]. These artifacts show traces of use as intermediate pieces in indirect percussion, i.e. the proximal end shows battering and crushing with some removal (mostly stepped) scars while the distal edge is broadly convex or straight with a beveled profile or may be pointed. In the Early Aurignacian these unworked or only partly worked tools are quite common and it is suggested that they were used for wood or bone working [37]. Intermediate pieces occur in the Middle Paleolithic but they are almost never described with at least one exception: a published example of an intermediate bone tool from Crimea [44].

We believe that the CdG intermediate tools may have been used for splitting elephant diaphyses once an incision was done with a flint flake. It is well known that elephant bones are difficult to break with stone hammers [3]. It is easier with an axe or a heavy iron blade [25].

Two intermediate pieces occur at La Polledrara (S8 Fig in S1 File) and [55 in S6 File: Fig 1–1]. Two intermediate pieces are also figured and described but not identified as such at Fontana Ranuccio [6: Fig 1, n.1 and n. 2]. Wedge-like bone tools have been described in the Middle Stone Age of South Africa at Sibudu [45] but the Italian pieces are much older, particularly Castel di Guido, Fontana Ranuccio and Malagrotta (S10B, S10C Fig in S1 File) all now dated to about 400 ka and associated with Acheulian industries, while La Polledrara is dated to 325 ka [12].

Thus the Aurignacian pieces do not represent a technical innovation [46] since direct use of unworked bone fragments clearly goes back to the Acheulian and the Middle Paleolithic. In this respect at least there was no total innovation and breakthrough in bone tool technology between the Lower-Middle and the Upper Paleolithic.

Note however that the Aurignacian tools are much smaller than the Latium Acheulian pieces. The Aurignacian tools have an average length of 10 cm and an average thickness of 1.6 cm [46] while the Acheulian tools made on massive elephant bone have a length from 14 to 24 cm and are thicker (2.9 to 8.0 cm). The difference depends on the kind of tools wanted and the size of the hunted species which provided the bone blanks. The Aurignacian fauna in Western Europe is dominated by large and medium-sized herbivores (bovids, equids, cervids). The elephant is not represented and this is likely due to the fact that the population of *Palaeoloxodon antiquus* was decreasing since the Last Interglacial and may have survived in southern Europe only as far as 34 ka [47].

The systematic organization of the bone industry with weapons (points) made on antler, ornaments in ivory and bone used for simple tools like smoothers and awls is typical of the Aurignacian. The first stage of bone production is the extraction, by debitage or breakage, of blanks of standardized form and dimension which will facilitate the manufacture of standardized tools [48]. The Olduvai bone tools were simply based on the selection of convenient bone fragments. But the CdG bone tools show systematic production of standardized blanks. However the degree of transformation and standardization of the finished product is limited. In the lithic industry the standardization will be introduced by the Levallois technique which produces thin and regular flakes that can be hafted easily. In the bone industry the mass production of identical objects will come with the Upper Paleolithic.

### The stone tool assemblage

The analysis of the lithic large tools was done for comparison with the bone industry. Differences depend mostly on the mechanical, structural qualities of bone versus silica and their reaction to water abrasion. The raw material of large and small tools is indicated in S5 Table and their state of preservation is in S4 Table (both in S1 File).

The lithic small tools of Castel di Guido are all of flint or other forms of microcrystalline silica. The latter is called “chert” by many archaeologists who distinguish flint as characterized by a fine, homogeneous, glossy texture with smooth fracture surfaces and chert as being coarse textured and opaque. Both rocks can produce a hard, regularly shaped edge. Compact bone, the outer dense layer of a long bone reacts rather well to percussion flaking. However, due to its inhomogeneous structure a flaked bone edge is weaker and less sharp compared to a flint in a tool of the same weight. This might explain why small tools of bone occur in very small numbers (N = 9) while lithic small tools are quite abundant (N = 94). The interpretation of this disparity is supported by the absence of bone cores. The limited shaping of bone large tools produced only a limited number of flakes of irregular shape while the debitage of bone cores is practically inexistent. In the CdG lithic assemblage there are 55 cores and 42% of small tools are made on thick flakes, like at Torre in Pietra level *m* [34].

Nearly all lithic small tools are fresh or only slightly abraded (87.5%) while the few small tools of bone are mostly abraded (7 of 9). The large bone tools are also more abraded than the large stone tools (S4 Table in S1 File). We know that abrasion on bone develops much more rapidly than on flint [49, 50]. Comparisons in morphology and typology of large tools of bone (Table 4) and of stone (Table 5) show evident similarities. The exceptions are the effect of raw material on small pieces (very few small tools in bone and concurrently very few cores) and the effect of blank shape (trihedrals cannot easily be made on diaphysis fragments).

**Table 5:** Castel di Guido. Counts of stone large tool types (≥ 6 cm in maximum dimension)*.

Type	N	%	Figures
Bifaces	15	16.0	Fig 16
Partial bifaces	10	10.6	Fig 17
Unifaces	7	7.4	Fig 17
Trihedrals	3	3.2	S12 Fig in S1 File
Pointed tools	14	14.9	Fig 18
Flaked pebbles with straight or convex edge (choppers)	24	25.5	---
Denticulates	4	4.3	---
Notches	3	3.2	Fig 18
Scrapers	3	3.2	Fig 18
Minimally modified or irregular morphology	7	7.4	---
Broken tools, retouch flake	4	4.3	---
**Total**	**94**	100	
Cores and core fragments	55	---	Figs 19–21
Percussors, anvils	6		S16 Fig in S1 File

*The reason for the size limit is explained in [34: p. 33].

**Fig 16:**
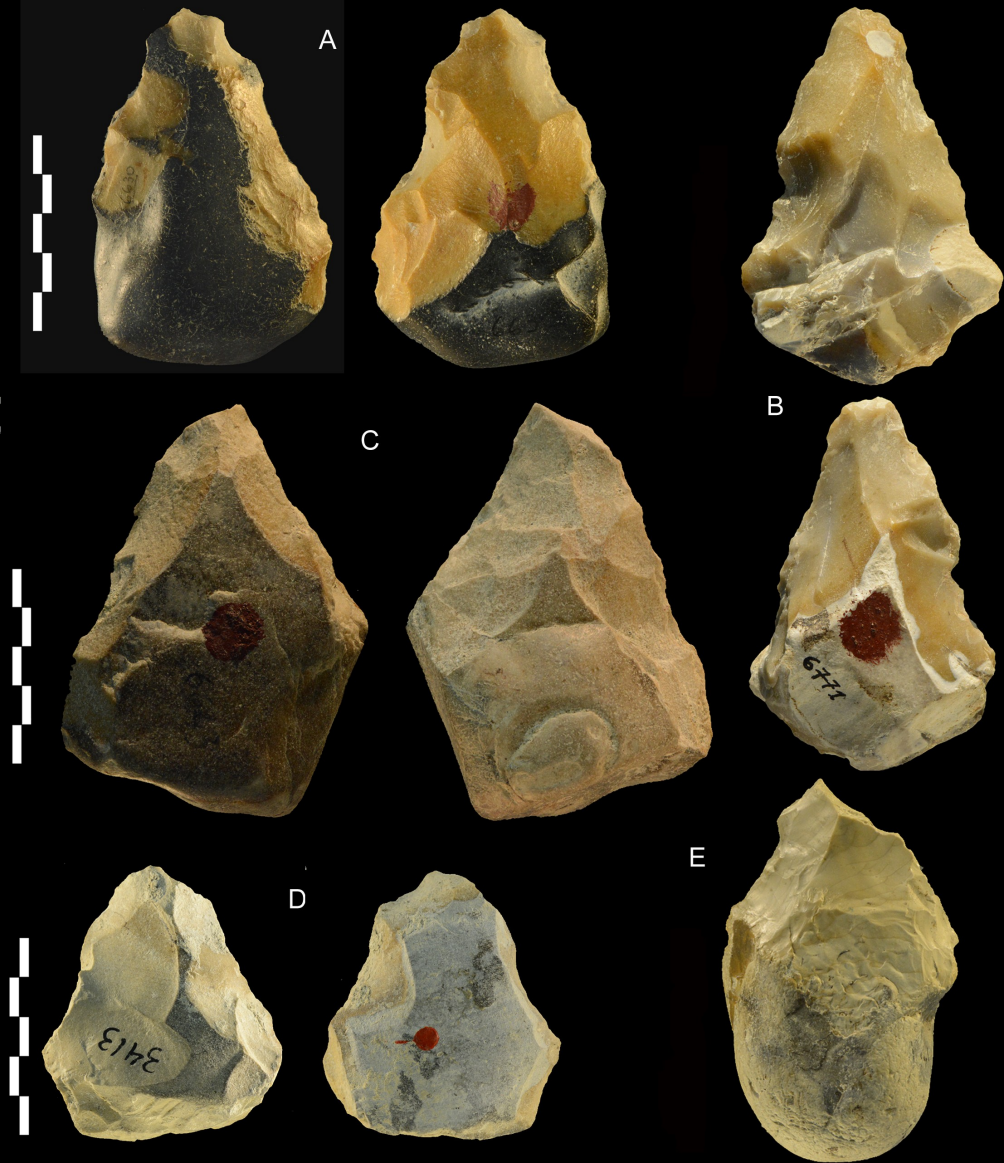
Castel di Guido lithics. Bifaces of flint (A, B, E) and chert (C, D). Catalogue numbers 6630, 6771, 6643, 3413, 1315. E might be considered as passing to partial biface but the lower face shows bifacial scars over half of the perimeter. A has double patina. Scale bar 5 cm.

**Fig 17:**
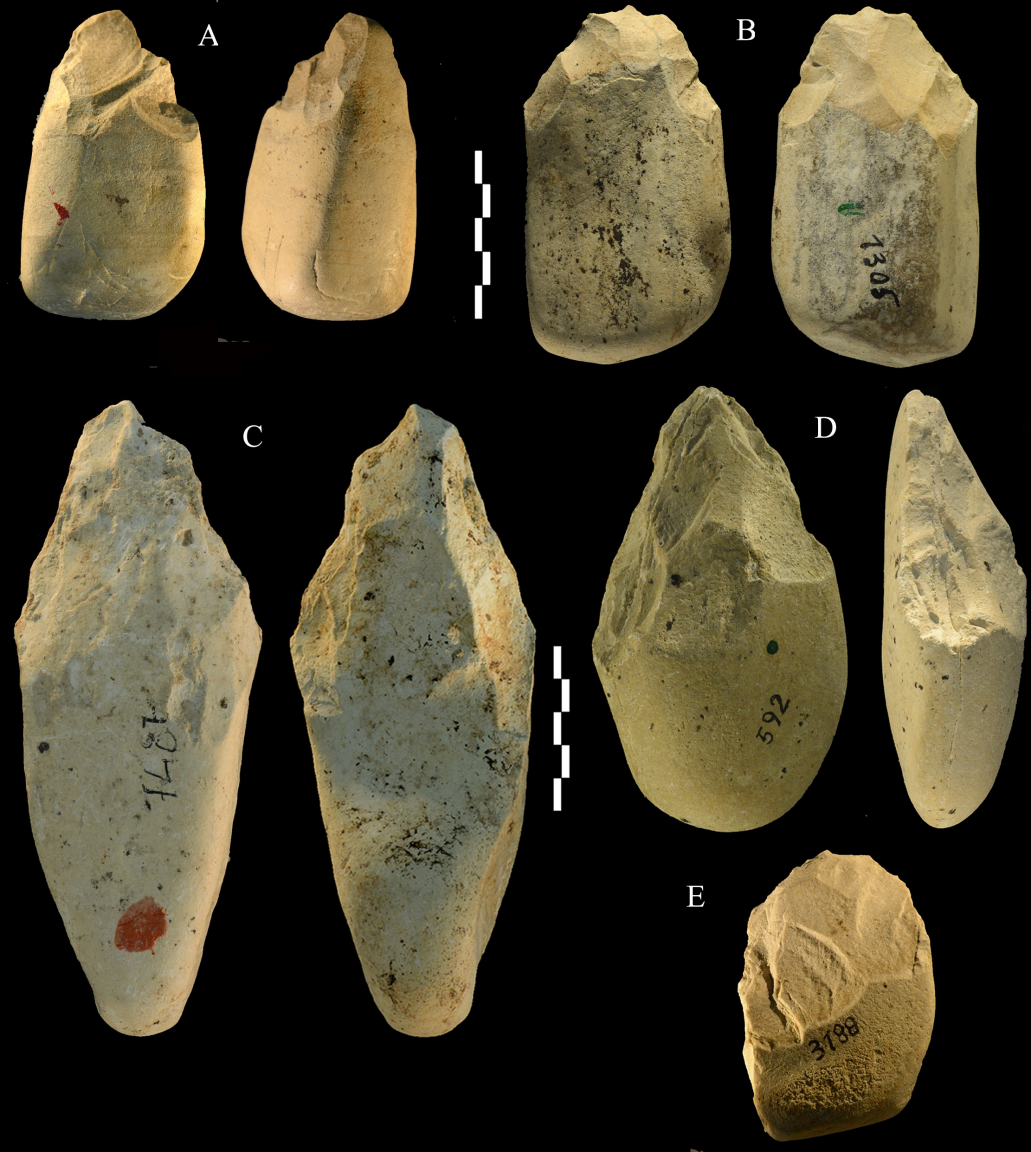
Castel di Guido lithics. Partial bifaces (A, B, C) and unifaces (D, E). A, B, E of chert, C and D of siltstone. Catalogue numbers 2398, 1305, 7487, 592, 3188. Scale bar 5 cm.

**Fig 18:**
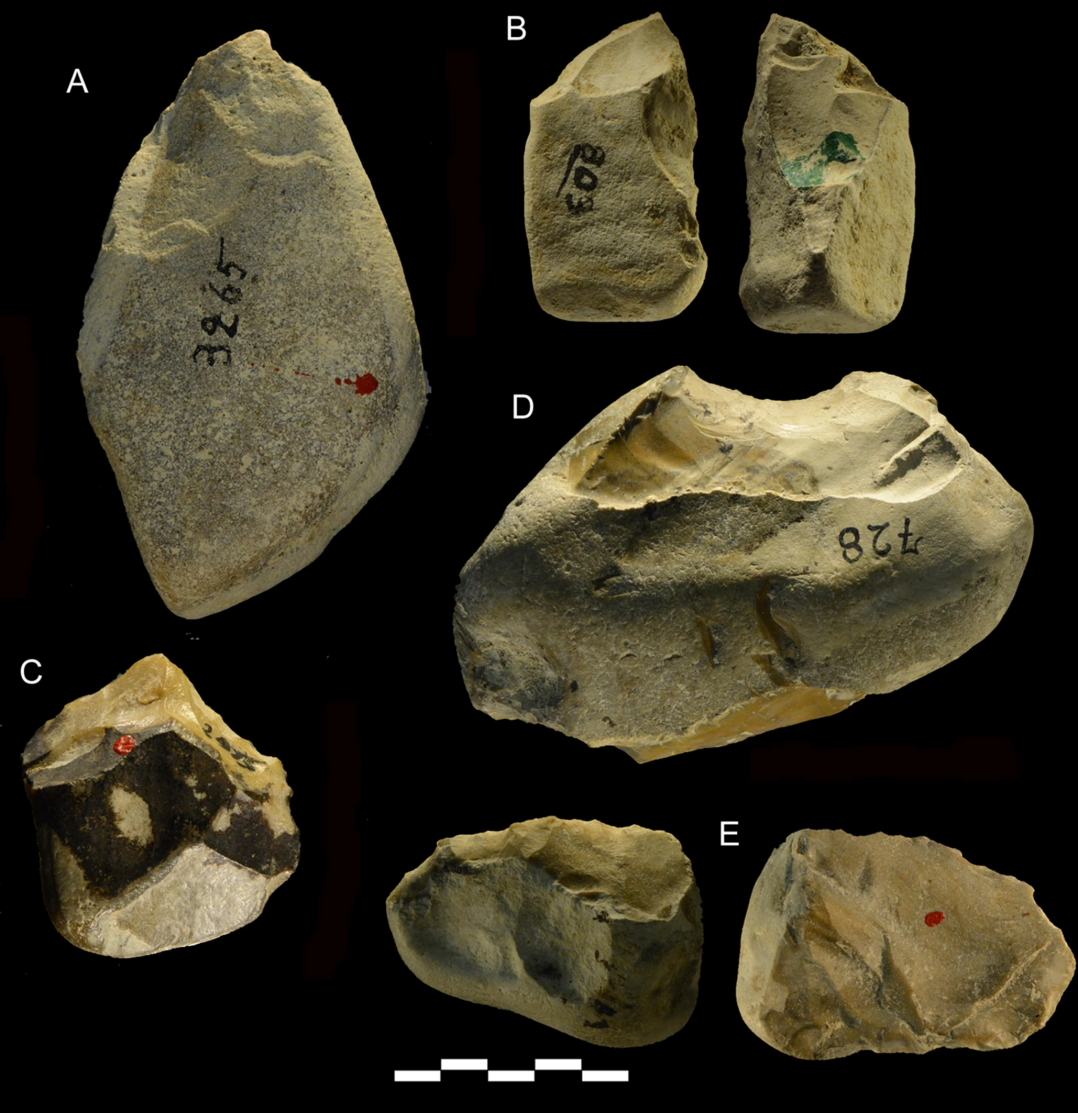
Castel di Guido lithics. A, B, E of chert, C, D of flint; (A, B, C) pointed tools; (D) retouched notch; (E) scraper edge on core. Catalogue numbers 3265, 803, 2915, 728, 3208. Scale bar 5 cm.

**Fig 19:**
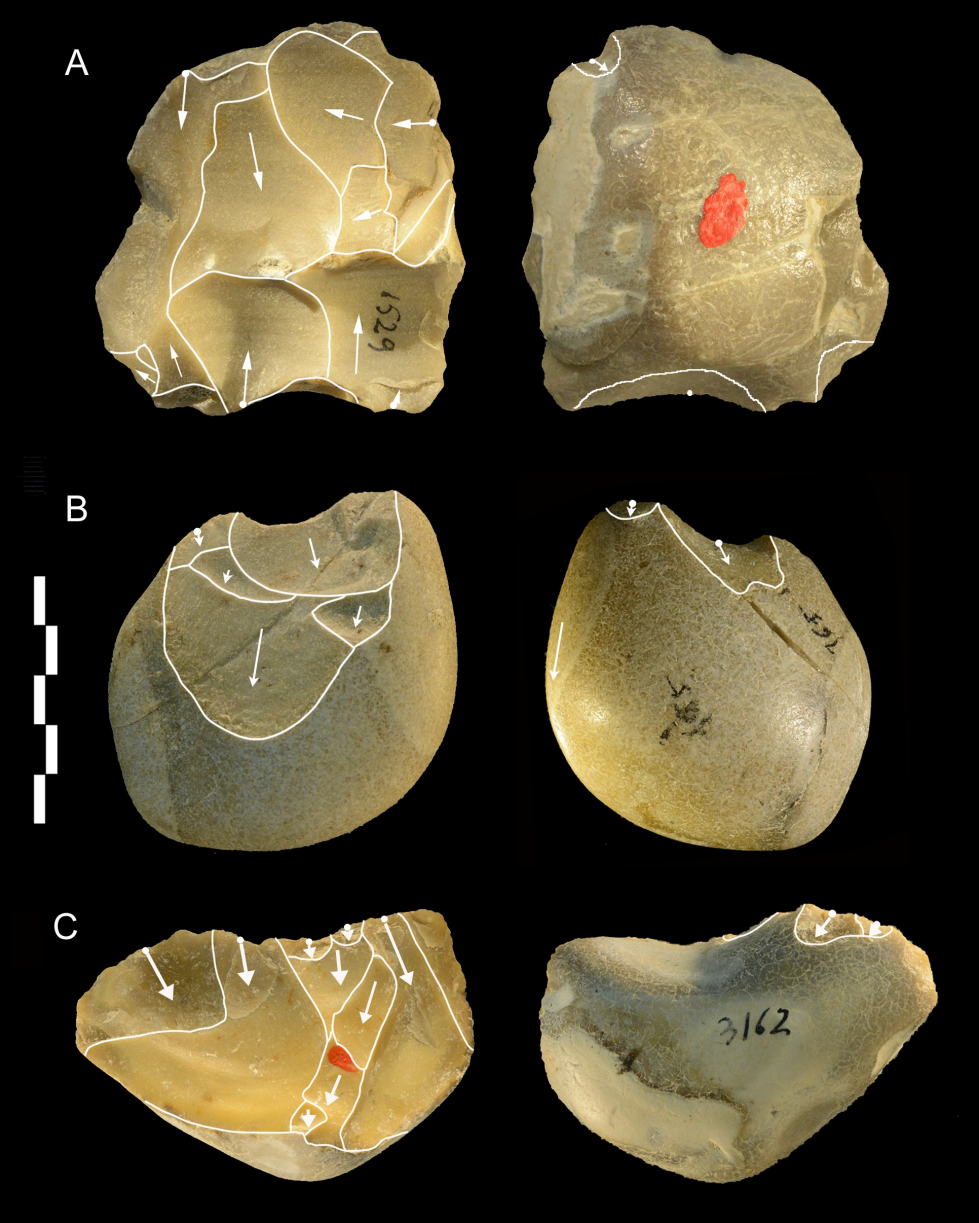
Castel di Guido lithics. Cores, A and C of flint, B of chert. All three have one debitage surface, A is centripetal, B and C are unidirectional. Catalogue numbers 1529, 165, 3162. Scale bar 5 cm.

**Fig 20:**
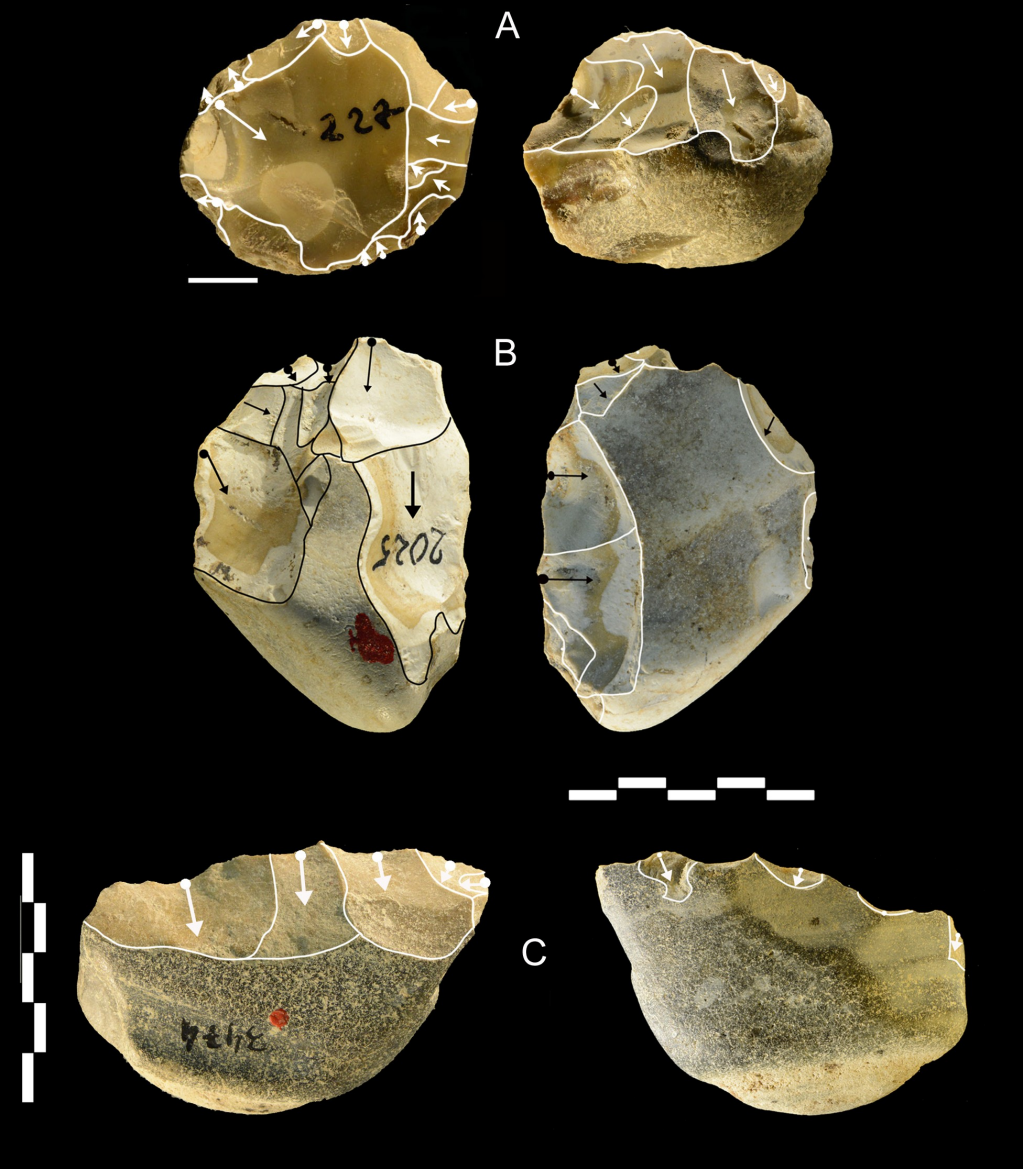
Castel di Guido lithics. Cores, A of flint, B and C of chert. A is centripetal non-Levallois, B is multidirectional and C is unidirectional. Catalogue numbers 227, 2065, 3474. Scale bar of A is 1 cm, of B and C is 5 cm.

**Fig 21:**
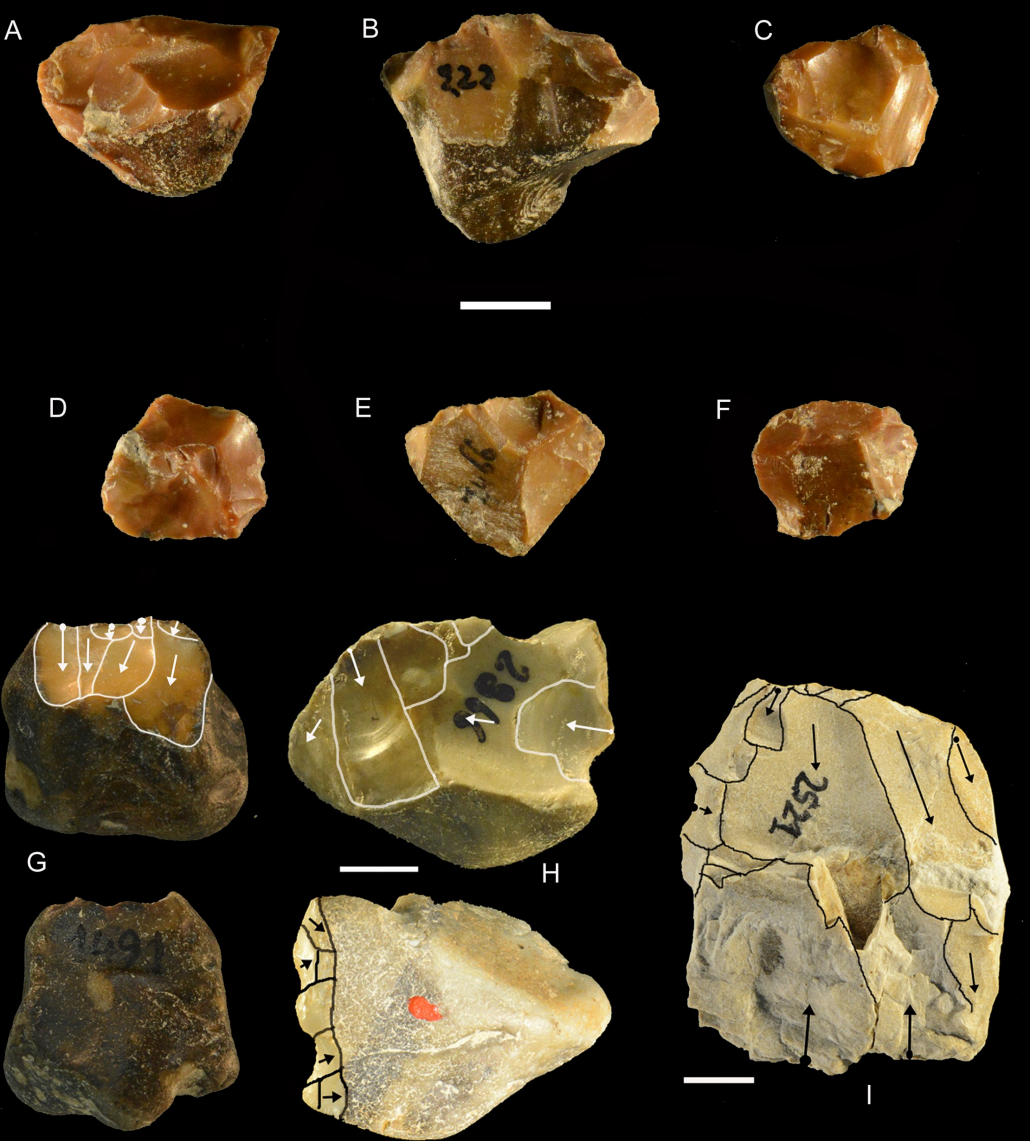
Castel di Guido lithics. Cores A-F are all of flint and all exhausted. Catalogue numbers 5158, 222, 3296,4394,7466,1991. G is of flint and is unidirectional. H has double patina indicating reuse. I is bidirectional but the lower removals are clearly failed due to the fissured and irregular texture of the raw material. Catalogue numbers of G-I are 1491, 2866, 2521. Scale bar 1 cm.

The size and shape of cores is quite variable. Cores do not show a stable debitage procedure, the flake removal is opportunistic. The lack of repeating features is evident, as at Torre in Pietra level *m* [34]). Cores were knapped in short sequences, without any special preparation of the debitage surface, with series of unidirectional removals, less commonly with bidirectional removals; two are centripetal cores. Flint was a desirable raw material and a large number of cores are exhausted (N = 16).

**Small tools (Tables 6 and 7).** The state of preservation and the kind of raw material are in S4 and S5 Tables in S1 File.

**Table 6:** Castel di Guido. Counts of stone small tools by major categories (< 6 cm).

Type	N	%
Scrapers (including side, double, convergent)	22	23.4
End scrapers	6	6.4
Denticulates	16	17.0
Notches	9	9.6
*Bec* *	10	10.6
Chopper, small tool on pebble	7	7.5
Retouched pieces	24	25.5
Total	94	100

*Bec is a French term indicating a short, thick awl formed by two opposing notches [33].

**Table 7:** Castel di Guido. Blanks of small tools*.

Categories	N	%
Flakes (31) and flake fragments (8)	39	46.4
Pebbles (23) and rolled blocks (3)	26	31.0
Cores (13), core fragments (1) and chunks (5)	19	22.6
Total	84	100

* Indeterminate blanks are excluded.

A few small tools at CdG are on a particular kind of core. Direct hard hammer percussion was used to split the pebble in half with the resulting flake as large as the core. We call the flakes “positive blank” and the core “negative blank” (S15 Fig in S1 File). These are special kinds of blanks which also occur in small numbers at Torre in Pietra level *d (*dated 270–240) at Sedia del Diavolo and Monte delle Gioie (dated 295–290 ka) and even at Grotta dei Moscerini [34: S1 File: Fig 1–1] [51: Fig 16] and [52, p. 2].

The small tool blanks of the Acheulian were thick and provided a relatively easy manual prehension. The flakes produced by the short reduction sequences of cores were also thick and almost all with some cortex which also facilitate manual prehension; partial bifaces have a cortical base. The Levallois method which produces thin and regular flakes without cortex is completely absent at Castel di Guido and at Torre in Pietra level *m* dated to about 350 ka.

The thickness/length ratio of the Castel di Guido small tools on flakes, cores and pebbles is on the average 0.4, while the mean thickness/length of Levallois flakes (from Torre in Pietra level d) is 0.2. The choice of Levallois thin flakes in the Middle Paleolithic is an innovation likely to correlate with the emergence of hafting [34: Fig 22].

## Conclusions

Our data shows that Castel di Guido is more than 100,000 years older than expected. The site is now dated to ~400 ka, i.e. to MIS 11, not to MIS 9—MIS 8 as in [53]. In spite of the fact that the number of bone bifaces had been largely overestimated, the number of verified, human-made bone tools is 98. This is the highest number of flaked bone tools from any Lower or Middle Pleistocene site published so far. The bone assemblage is characterized by a clear diversity of tool types and by the systematic production of diaphysis fragments as tool blanks. The CdG bone tools are neither expedient nor occasional.

The capacity of producing series of identical blanks is the first phase of most *chaînes opératoires*. Systematic production of blanks of standardized form and dimensions will facilitate the manufacture of standardized tools. However at CdG the degree of transformation and standardization of the finished product is limited. Mass production of identical objects (e.g. projectile points) will come with the Upper Paleolithic when shaping of bones, especially of elephants, by percussion flaking was abandoned, likely because of the decline and extinction of *Palaeoloxodon* in the Late Pleistocene of Western Europe, and because more complex techniques of bone shaping became common and adapted to a smaller fauna. A clear example is the exploitation of deer antler to make projectile points. However, smoothers from CdG and Schöningen and the intermediate pieces prove that some aspects of Aurignacian technology have roots that go back to the Middle Pleistocene.

The CdG hominids had reached the first step in the process of increasing complexity of bone technology. Yet the intensive and systematic production and use of elephant diaphyses of standardized shape appears as a unique occurrence, the CdG hominids simply taking advantage of the occasional abundance of elephant bones to overcome the difficulty of obtaining lithic raw material for the production of large-size tools. Throughout the Middle Pleistocene and the early part of the Late Pleistocene the human involvement with *Paleoloxodon* bones is rare, reflecting the decline and final disappearance of *Paleoloxodon* from southern Europe during MIS 3. It is clear that the cognitive capacities of the CdG hominids had reached the level of technological ability needed for the making of repetitive reduction sequences and the mass production of standardized tools. Yet these abilities remained an isolated phenomenon, at least in the evolution of bone technology of the Middle Pleistocene. The complex bone technology of the Upper Paleolithic is not the exclusive marker of the superior cognition of modern humans since it reflects, at least in part, a change in the hunting equipment in response to the need of light projectile weapons. The heavy wooden spears and spears armed with Levallois and Mousterian lithic points of the Middle and early Late Pleistocene hunters were replaced by lighter throwing weapons with split-based points made on antler and lithic bladelets as armatures [54]. Thus the view that early humans were incapable of developing sophisticated techniques diagnostic of behaviorally modern humans is unsupported. The emergence of complex bone technology at the end of the Mousterian period appears to be more a matter of technical evolution than an innovation due to higher levels of cognition.

## Supporting information

S1 File Figures and tables. (PDF)

S2 File Geochronological and stratigraphic. (PDF)

S3 File ^40^Ar/^39^Ar dataset. (PDF)

S4 File Permissions from copyright holders. (PDF)

S5 File Behavioral complexity of the Castel di Guido assemblage. (PDF)

S6 File References 55–81 for supporting information. (PDF)
