# Correction: Elephant bones for the Middle Pleistocene toolmaker

**DOI:** 10.1371/journal.pone.0306788

**Published:** 2024-07-08

**Authors:** Paola Villa, Giovanni Boschian, Luca Pollarolo, Daniela Saccà, Fabrizio Marra, Sebastien Nomade, Alison Pereira

An additional affiliation is missing for the second author. Giovanni Boschian is also affiliated with the Palaeo-Research Institute—University of Johannesburg, P.O. Box 524, Auckland Park, 2006, South Africa.
