# Supplementary material for: Elephant bones for the Middle Pleistocene toolmaker
Source: PLoS One. 2021 Aug 26;16(8):e0256090. doi: 10.1371/journal.pone.0256090 (PMC8389514; doi:10.1371/journal.pone.0256090)
Supplement: S1 File — (PDF) [file pone.0256090.s001.pdf]

Supporting Information

Elephant bones for the Middle Pleistocene toolmaker

Paola Villa*,Giovanni Boschian, Luca Pollarolo, Daniela Saccà, Fabrizio Marra, Sebastien Nomade, Alison Pereira.

Correspondence to: villap@colorado.edu

S1 File. Figures and Tables

This PDF file includes:
- Figures S1- S16
- Tables S1- S5

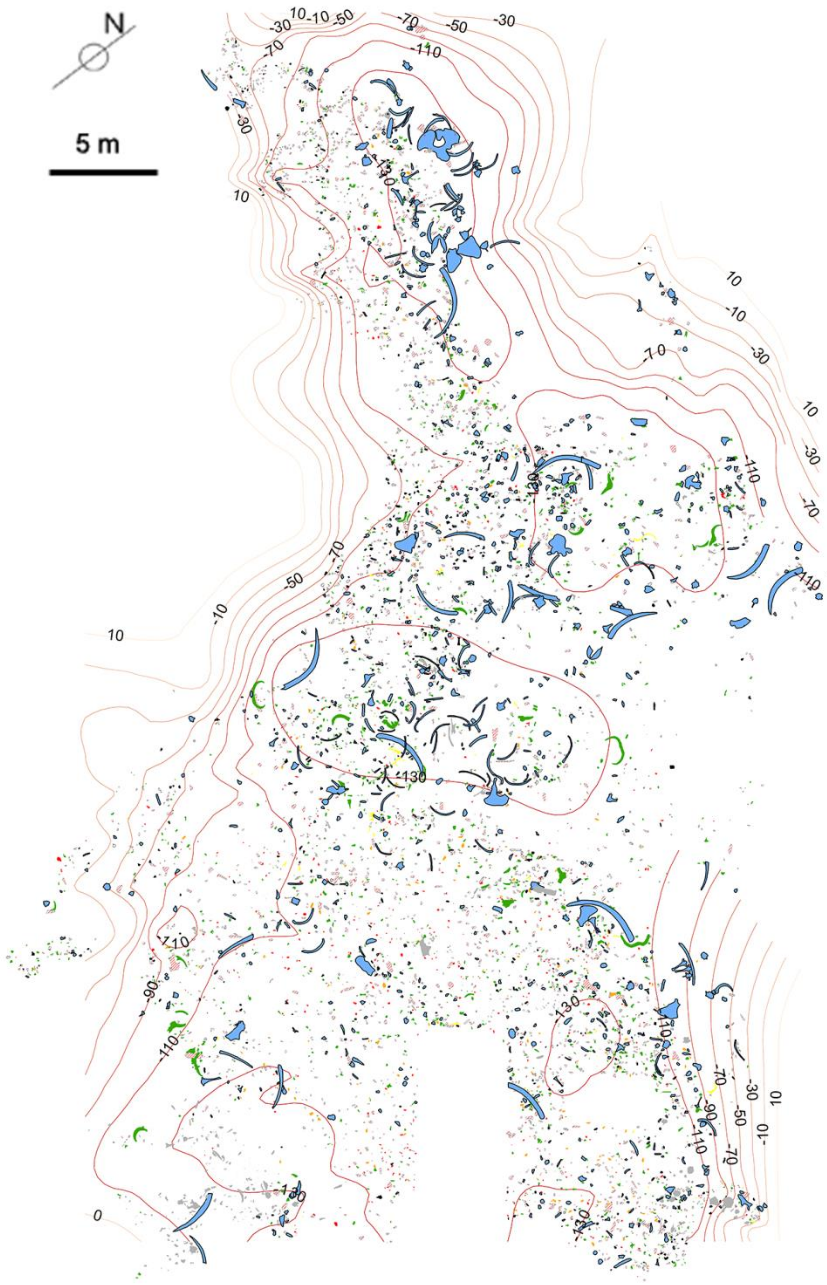

Fig S1. Castel di Guido excavation area. Distribution plan of the remains and contour map of the topography. Elephant bones in blue. Drawing by G. Boschian.

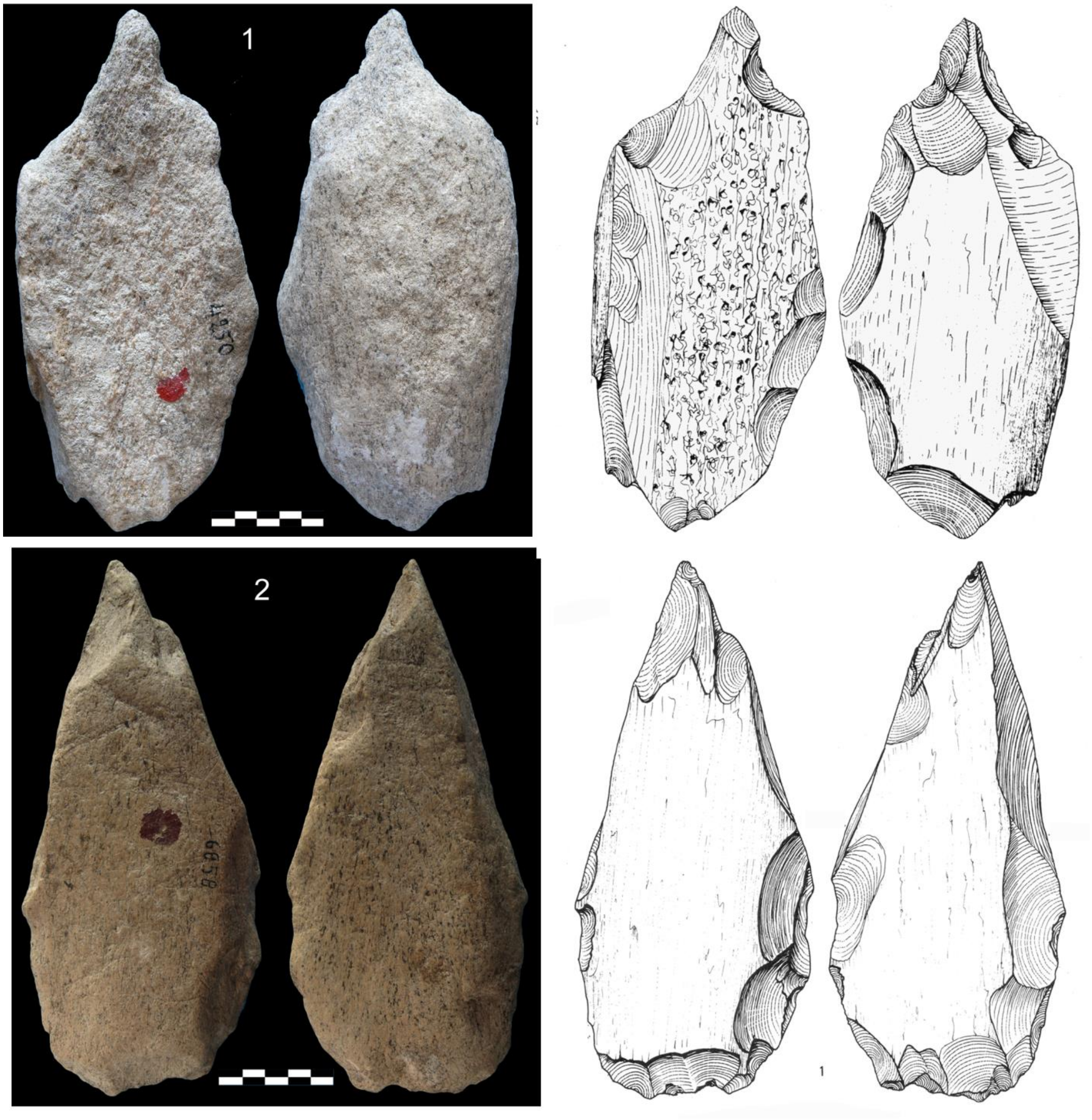

Fig S2. Comparison of photos and drawings in the 1996 monograph. (1) Pointed tool, very abraded, catalogue number 4250, photo by G. Boschian. (2) Pointed tool, abraded, catalogue number 6858, photo by G. Boschian. In the monograph this piece is classed as a biface.

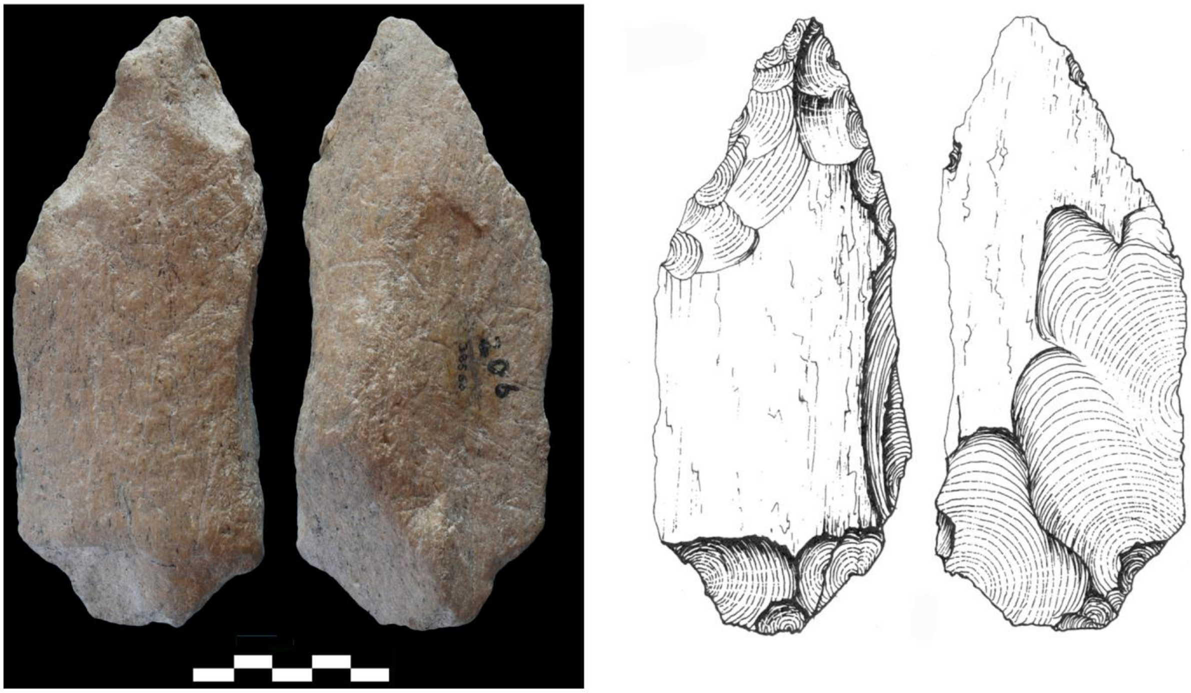

Fig S3. Comparison of photo and drawing in the 1996 monograph. Pointed tool, catalogue number 206, abraded. Photo by G. Boschian.

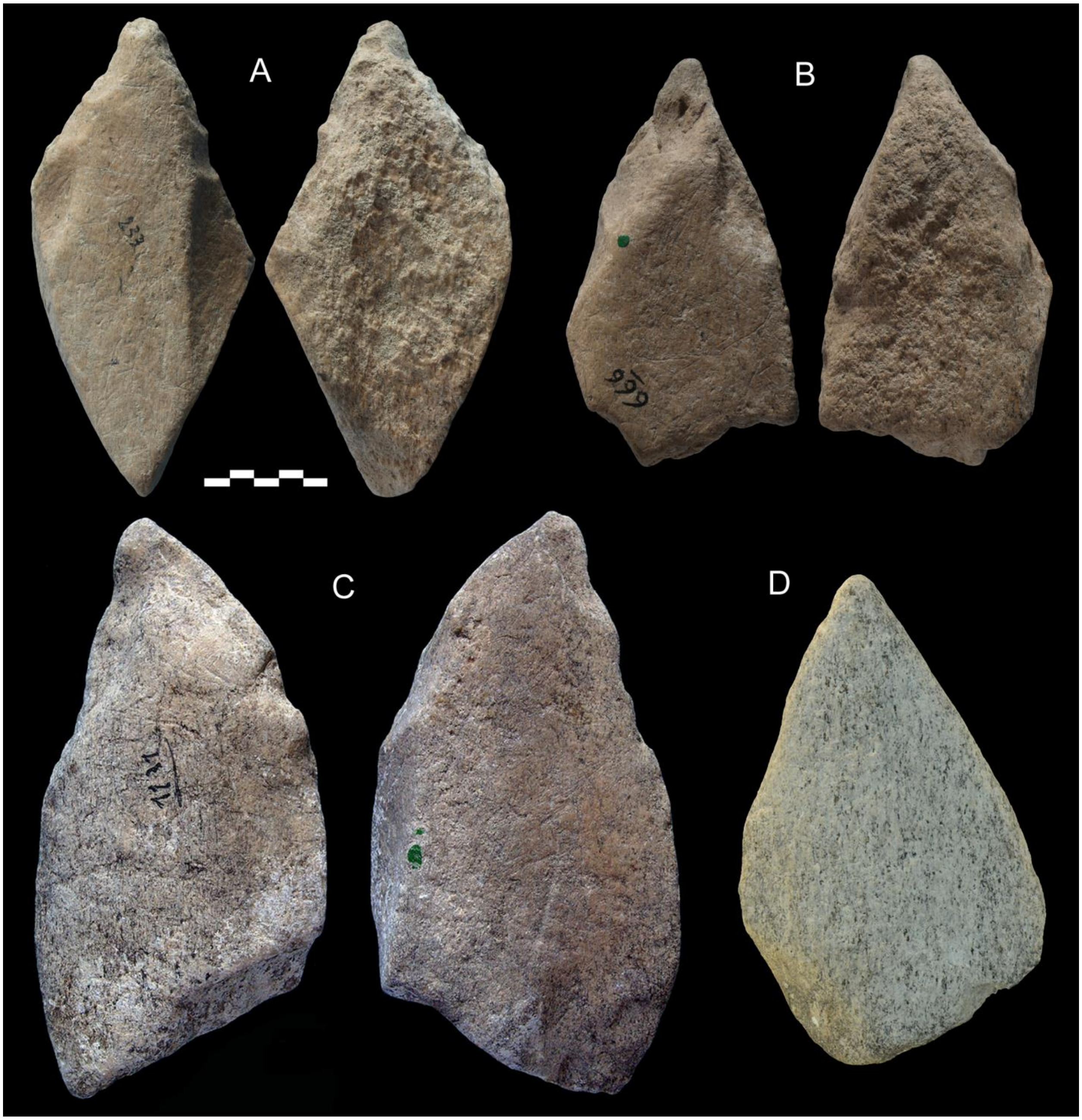

Fig. S4. Examples of bone artifacts classed as bifaces in the 1996 monograph. (A-D) catalogue number 233, 666, 1131, 7588. We identify them as pointed tool (A), pointed wedge (B, the base is battered), pointed tool but too abraded for sure diagnosis (C) and unmodified (D). Photos G. Boschian and P. Villa.

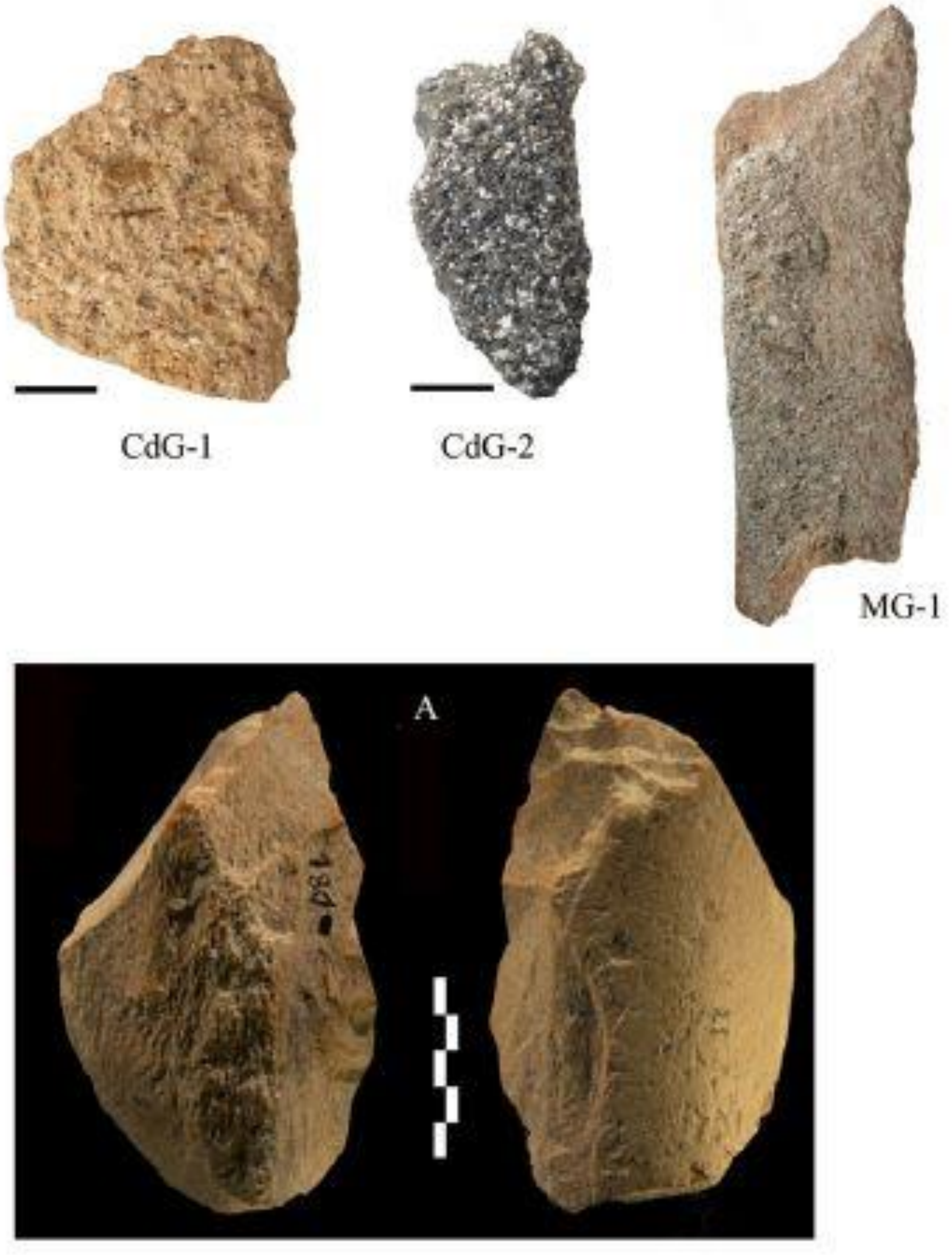

Fig S5. Top: Two dated samples from Castel di Guido and one from Malagrotta.
Bottom: Pointed bone tool from Malagrotta

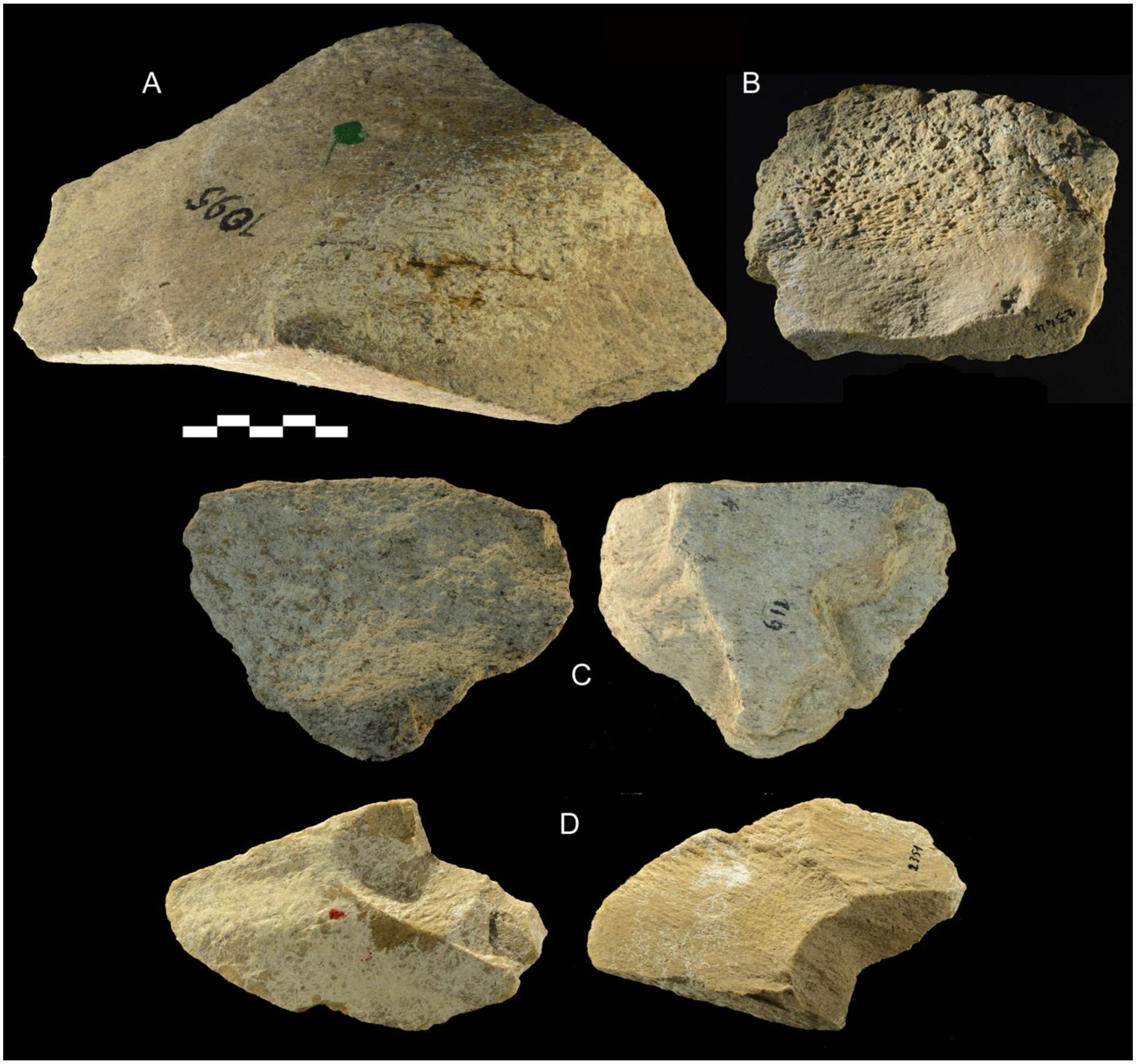

Fig S6. Unretouched flakes. A is cortical, C and D are partly cortical, B is taken in the thickness of the cancellous bone. C is more abraded on the dorsal face. Catalogue number 1095, 2344, 119, 2351.

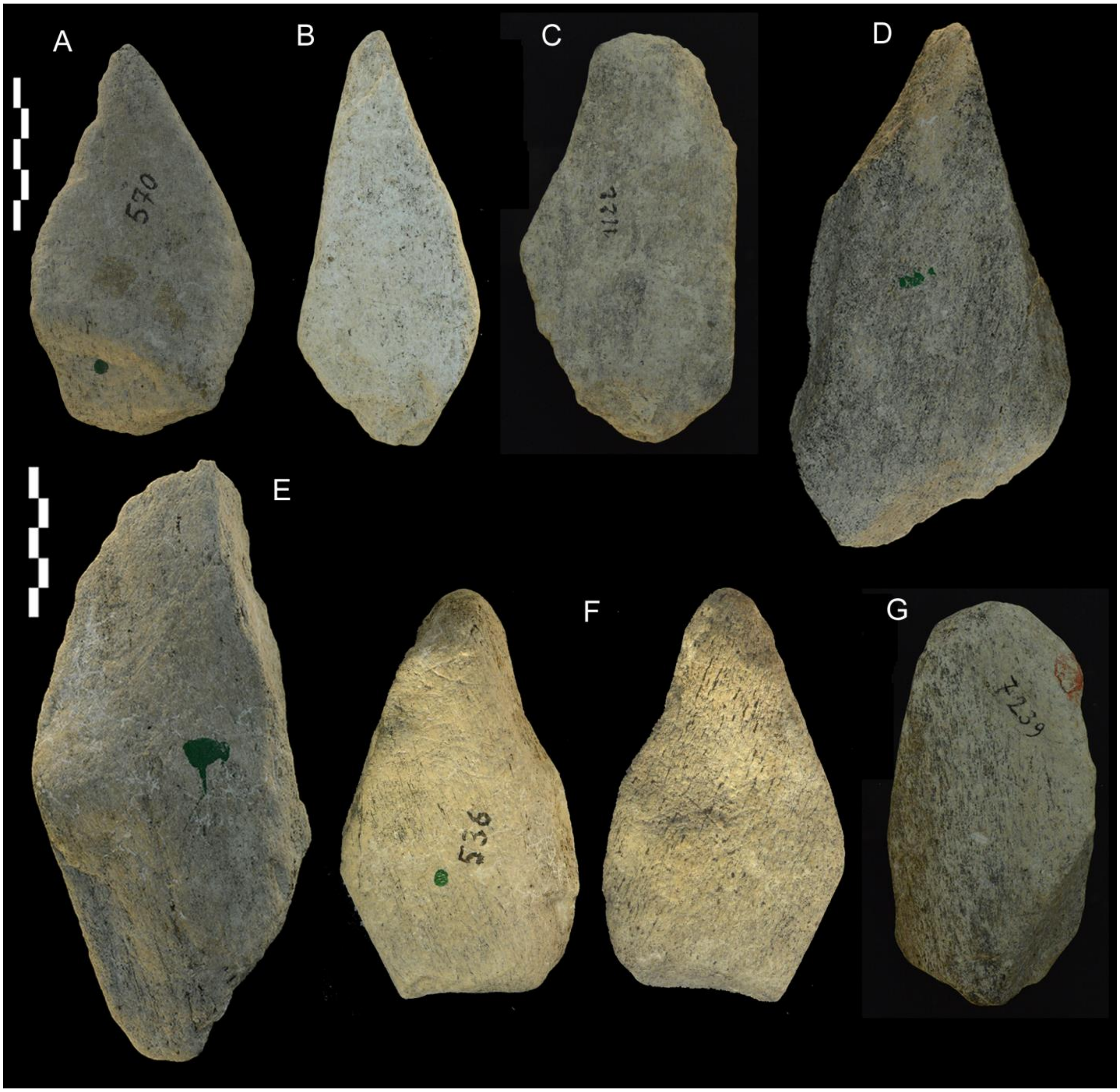

Fig S7. Examples of unmodified diaphysis fragments. (A-G) Catalogue number 570, 610, 1122, 1341, 1255, 536, 7239. The number 536 (F) was classed as biface in the 1996 monograph. Photos G. Boschian and P. Villa.

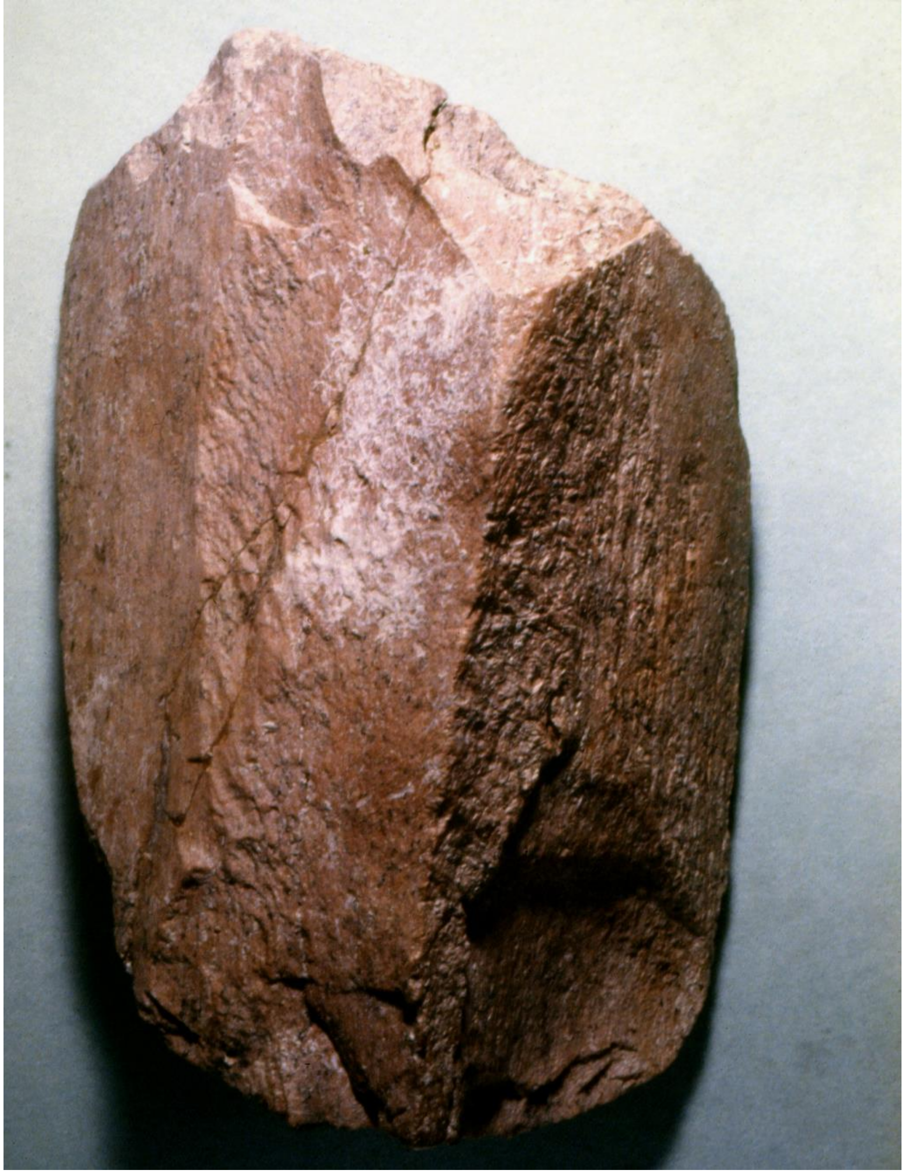

Fig. S8. Intermediate piece from La Polledrara.
Length 24 cm. Photo P. Villa

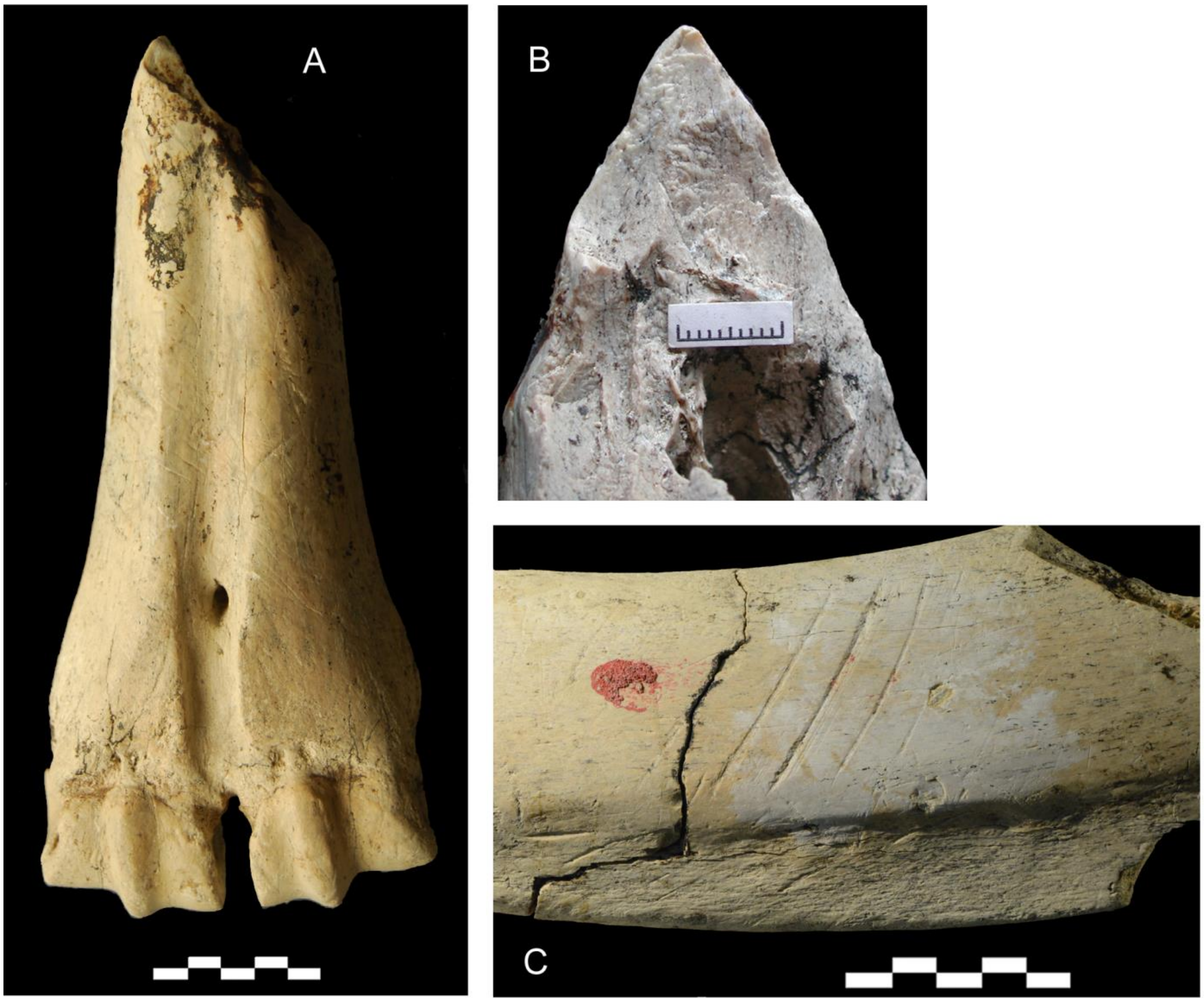

Fig S9. Castel di Guido. (A) Pointed tool on a *Bos* middle-distal metatarsal, catalogue number 5482. (B) detail of the pointed end shaped by a number of removals. Modified from (22: fig.5). Courtesy of Daniela Saccà. (C) elephant rib with cutmarks, catalogue number 5220. This is one of the few pieces with clearly identifiable human-made cutmarks, due to the good state of preservation of the surface.

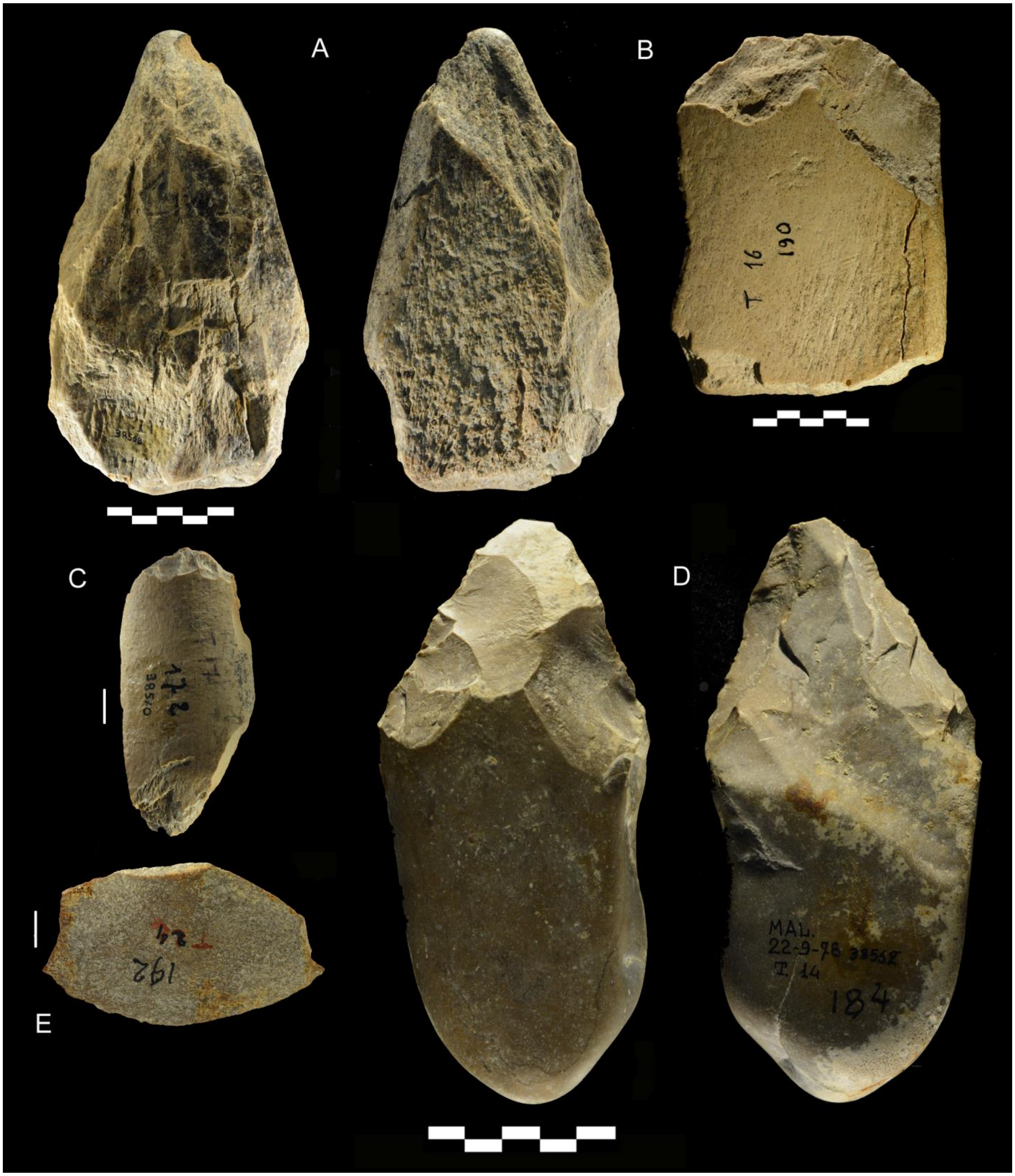

Fig S10. Malagrotta bone and stone artifacts. (A) Bone biface made on diaphysis fragment, slightly abraded. (B) Bone wedge. (C) Diaphysis fragment (of bovid size) possibly used as a wedge. (D) Partial biface of siliceous limestone. (E) Ventral face of a bone flake with some retouch/utilization on right side. Scale bar 5 cm.

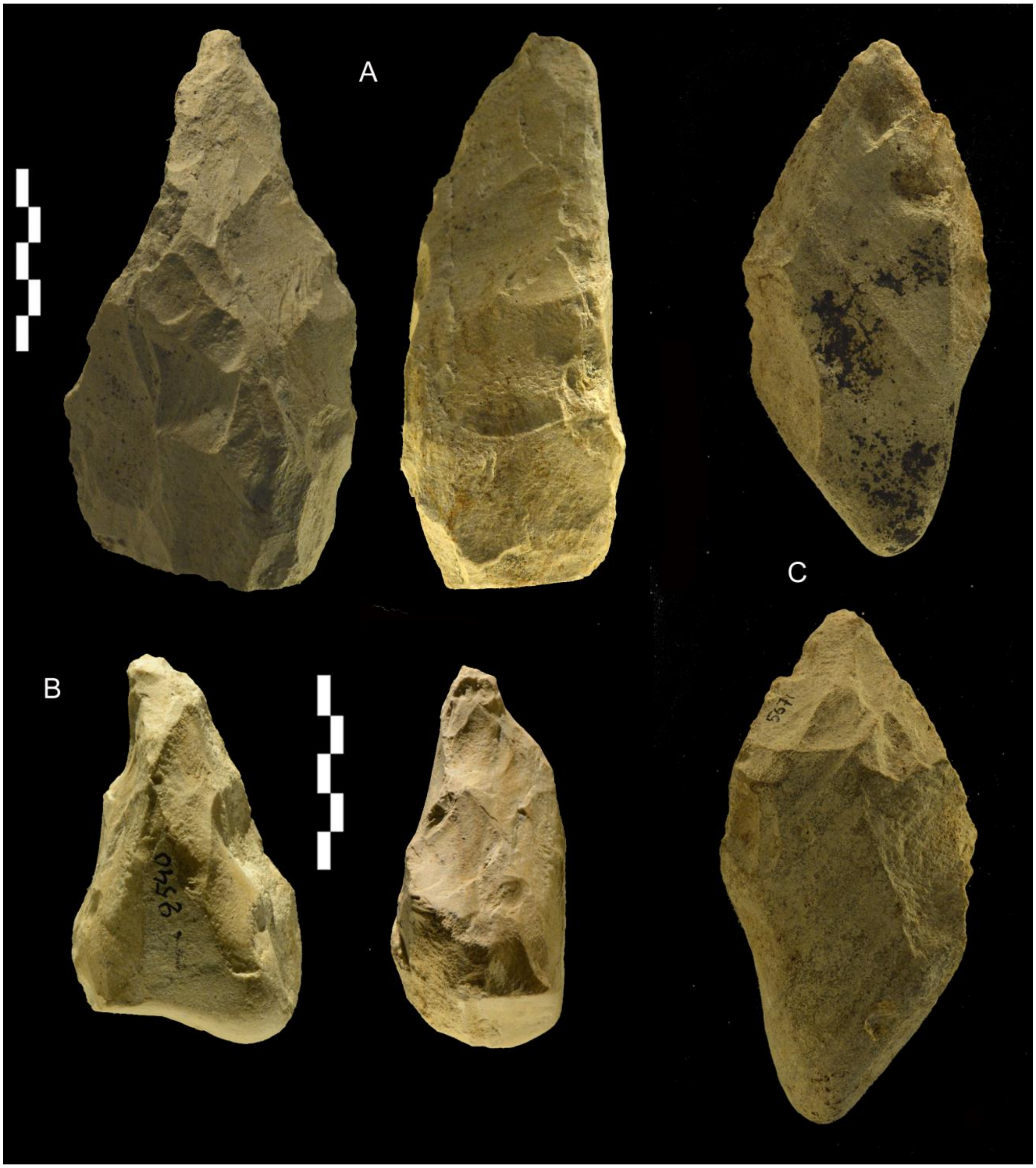

Fig S11. Castel di Guido lithics. A-B trihedrals; upper face and right side of A, upper face and left side of B. C is a biface of chert. Catalogue number 3000, 2540, 5671. Scale bar 5 cm.

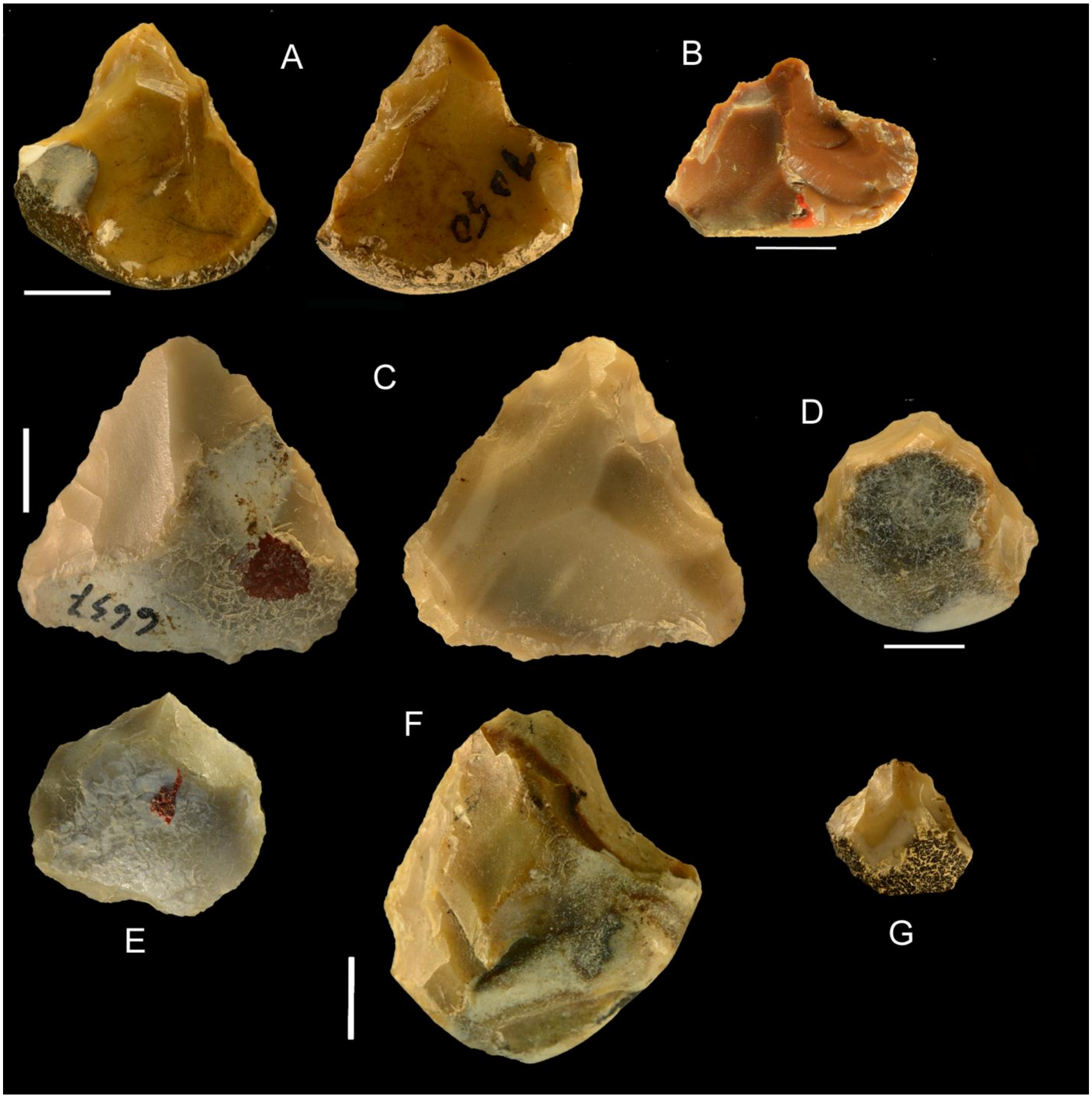

Fig.S12. Castel di Guido lithics. Small tools on flake, all of flint. (A, B, E) short and thick awl, “bec” in French terminology. (C) convergent denticulate. (D, G) end scraper. (F) side scraper with a notch on the other side. Catalogue number: 1050, 3446, 6637, 3564, 3318, 2676, 6928. Scale bar 1 cm.

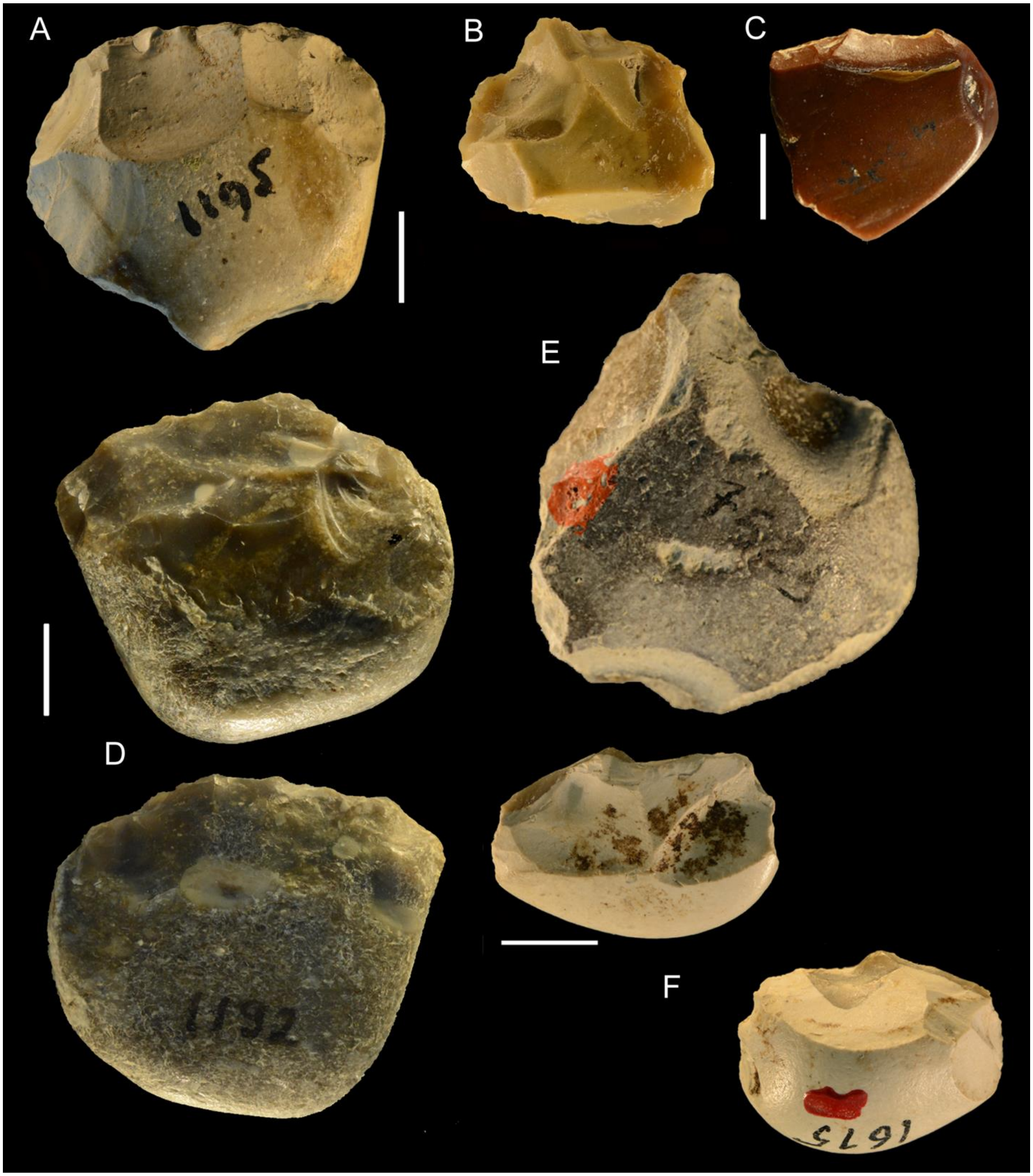

Fig S13. Castel di Guido, small tools on pebble (A, C, D-F) and rolled block (B), all flint except A, E which are of chert. Catalogue number 1195 (bifacial ly worked), 2136 (side scraper reworked in denticulate) 4398 (unifacial retouch), 1192 (bifacial retouch), 7525 (denticulate with a short awl-bec-), 1615 (bifacially worked as a core but with some retouch). Scale bar 1 cm.

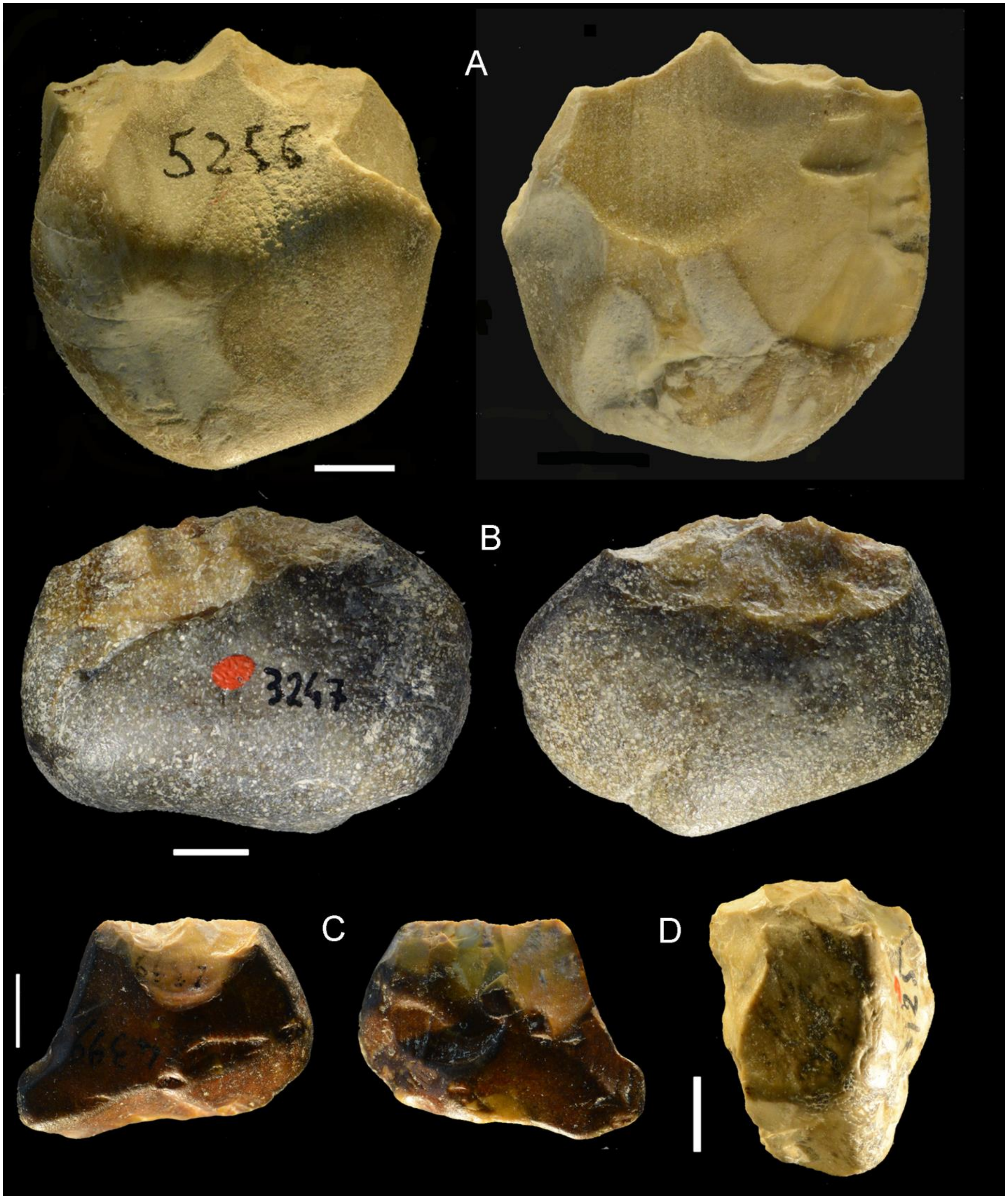

Fig.S14. Castel di Guido, small tools on pebble, A-B of chert, C-D of flint. A and B have L = 55 and 57 mm and are at the limit of the small tool category. A is a “bec”, B is a retouched piece, C has a bifacial edge and D is an end scraper on a core. Catalogue numbers 5256, 3247,4399,3125. Scale bar 1 cm.

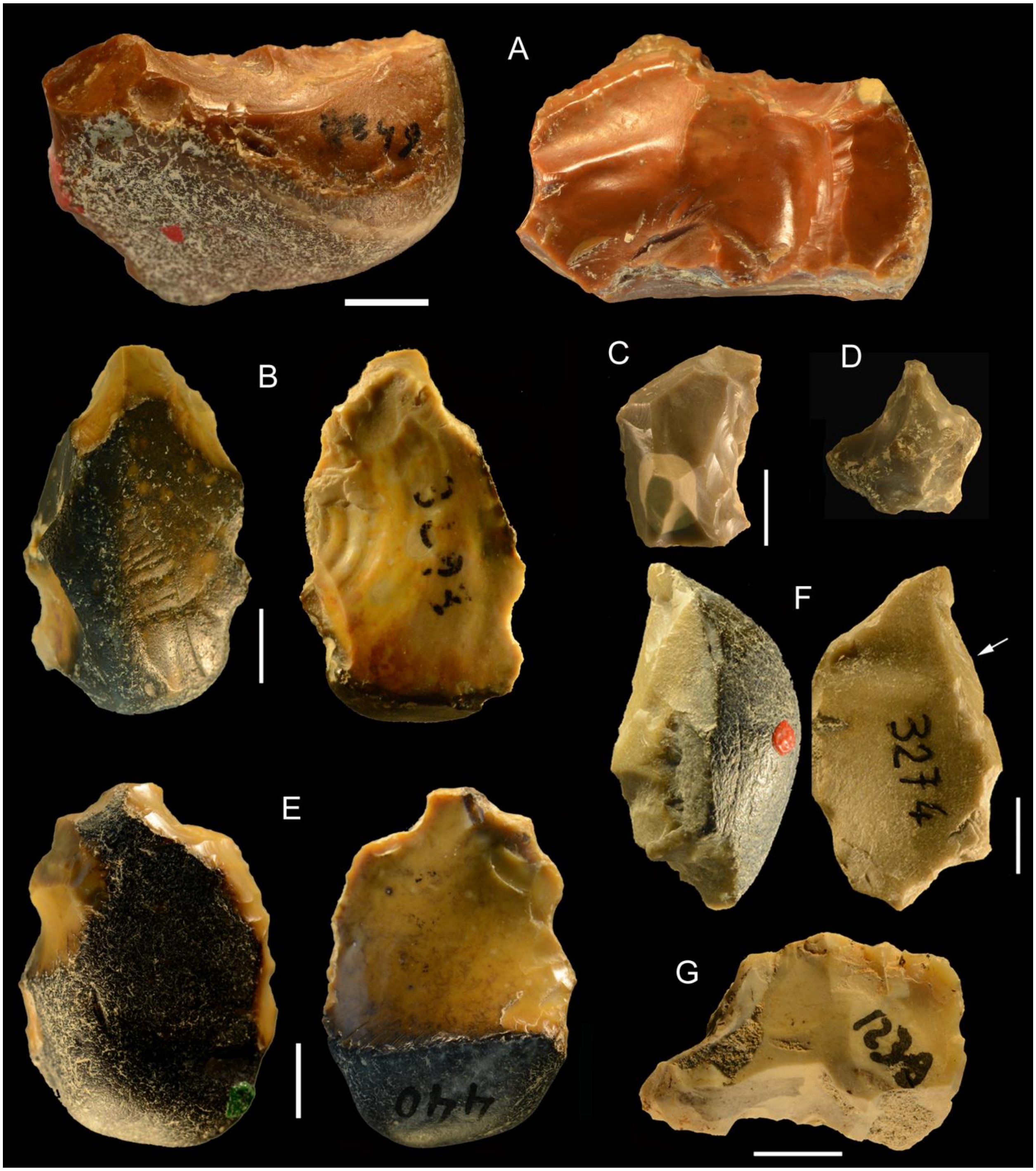

Fig.S15. Castel di Guido lithics. Small tools on core, all of flint. (A) side scraper on unidirectional core; (B, E) denticulates on a negative blank (Villa et al. 2016 p. 33); (C) concave scraper; (D) bec on core; (F) negative blank with some retouch, the arrow indicates a break prior to retouch; (G) retouched notch, note the double patina. Catalogue numbers 4849, 3293, 1388, 214, 440, 3274,1238. Scale bar 1 cm.

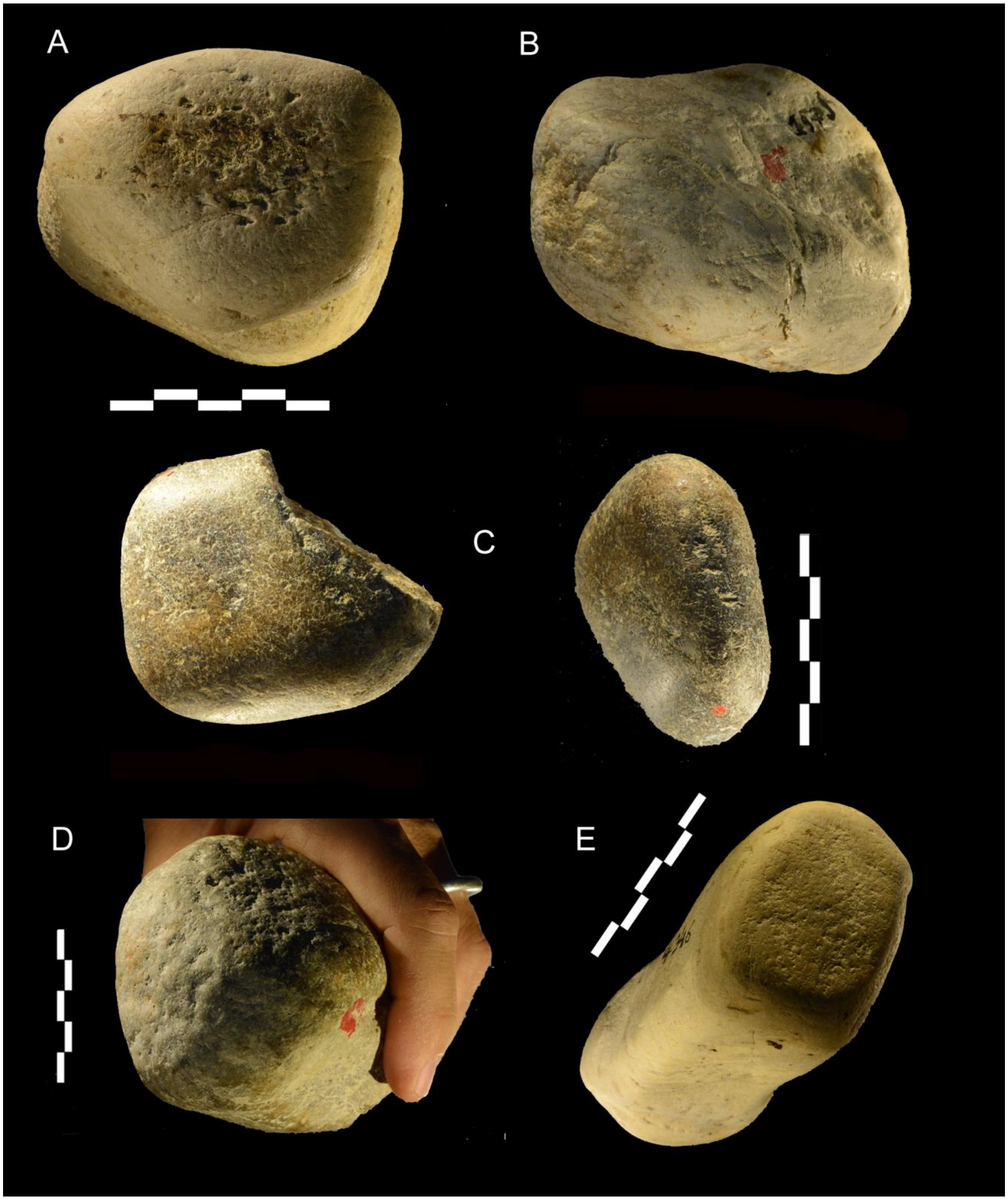

Fig S16. Percussors/anvils. A,B,C,E chert, D trachyte. A is 381.6 gms; B is 427.4 gms; C is 203 gms; D is 1008.6 gms; E is 363 gms. Catalogue numbers 1219, 1575, 3610, 4683, 4346. Scale bar 5 cm.

Tables S1-S5

Table S1. Middle Pleistocene European and Western Asia sites with flaked bone tools

**Site**	**Age**	**No. of bone tools**	**References**
Bilzingsleben (Germany) Archaeological layer at the base of a travertine sequence	234U/230Th and ESR, ~350 000 - 420 000 years	119 bone tools according to Mania. But the number is controversial for lack of technological and taphonomic analyses.	(56-58)
Rhede (Germany)	No date	1 biface of mammoth bone.	(59)
Spiennes (Belgium)	No date	1 minimally modified pointed tool of a large size mammal.	(60*)*
Torralba (Spain)	MIS 7, younger than Ambrona (which is dated to end of MIS 11).	Large but unspecified number of bone tools made on elephant bones and other smaller mammals. Analysis based on morphology, not on shaping technology. Controversial.	(61-64)
Marathousa (Greece)	500-400 ka BP	1 flake,1 denticulate, 1 percussor	(65)
Revadim (Israel)	500-300 ka	2 bone bifaces	(66)

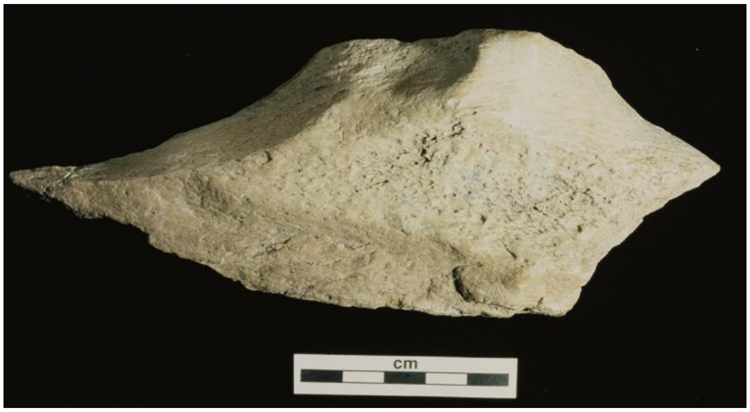

Torralba, possible bone tool. Catalogue number Q 1124

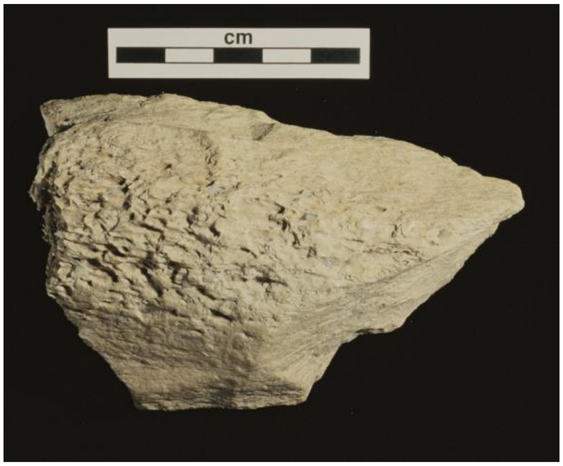

Torralba, possible bone tool.
Catalogue number Q 1868.

Table S2. The faunal assemblage of Castel di Guido [from 22].

Taxa	NISP	MNI	
		Adult	Young
Bos primigenius	1399	42	1
Palaeoloxodon antiquus	1381	10	1
Equus ferus	385	15	1
Cervus elaphus	71	12	-
Cervidae	2	1	-

Other mammalian taxa (carnivores, lagomorphs) are represented by only few or single finds). 792 remains were unidentified or classed by size.

Table S3. Castel di Guido. State of preservation of the main faunal species. From [22}.

State of preservation	*Palaeoloxodon*	*Bos primigenius*
Fresh	6.9	5.5
Slightly abraded	43.8	51.5
Abraded	45.7	41.0
Very abraded	3.6	1.9

Table S4. Castel di Guido. Stone and bone tools by state of preservation

**State of preservation**	**Stone small tools**		**Stone large tools**		**Bone large tools**		**Bone small tools**	
	N	%	N	%	N	%	N	%
Fresh	52	59.1	28	33.7	6	6.8	-	-
Slightly abraded	25	28.4	24	28.9	16	18.2	2	22.2
Abraded and very abraded	1	1.1	20	24.2	30	34.1	2	22.2
Faces with different abrasion	6	6.8	9	10.8	28	31.8	3	33.3
Reused, recycled (with younger retouch on an abraded or patinated piece)	4	4.6	2	2.4	8	9.1	2	22.2
Total	88	100.0	83	100.0	88	100.0	9	100.0

Note. Indeterminate cases are excluded.

Table S5. Castel di Guido, Counts of stone tools by raw material

**Raw Material**	**Small tools**		**Large tools**	
	**N**	**%**	**N**	**%**
Flint	66	70.2	7	7.4
Chert	27	28.7	64	68.1
Siltstone	1	1.1	22	23.4
Other	-	-	1	1.1
Total	94	100	94	100

Note. This table shows a clear reversal in the frequency of flint between small and large tools. It has been suggested that the small size of the flint pebbles and the need to have larger blanks for tool manufacturing contributed to the use of bone as raw material [*4*].
