# Supplementary material for: Elephant bones for the Middle Pleistocene toolmaker
Source: PLoS One. 2021 Aug 26;16(8):e0256090. doi: 10.1371/journal.pone.0256090 (PMC8389514; doi:10.1371/journal.pone.0256090)
Supplement: S2 File — (PDF) [file pone.0256090.s002.pdf]

Supporting Information

Elephant bones for the Middle Pleistocene toolmaker

Paola Villa*,Giovanni Boschian, Luca Pollarolo, Daniela Saccà, Fabrizio Marra, Sebastien Nomade, Alison Pereira.

Correspondence to: villap@colorado.edu

S2 File. Geochronological and stratigraphic data

This PDF file includes:
Text
Figures S17-S19

Geochronological and stratigraphical data

by

Fabrizio Marra, Sebastien Nomade, Alison Pereira

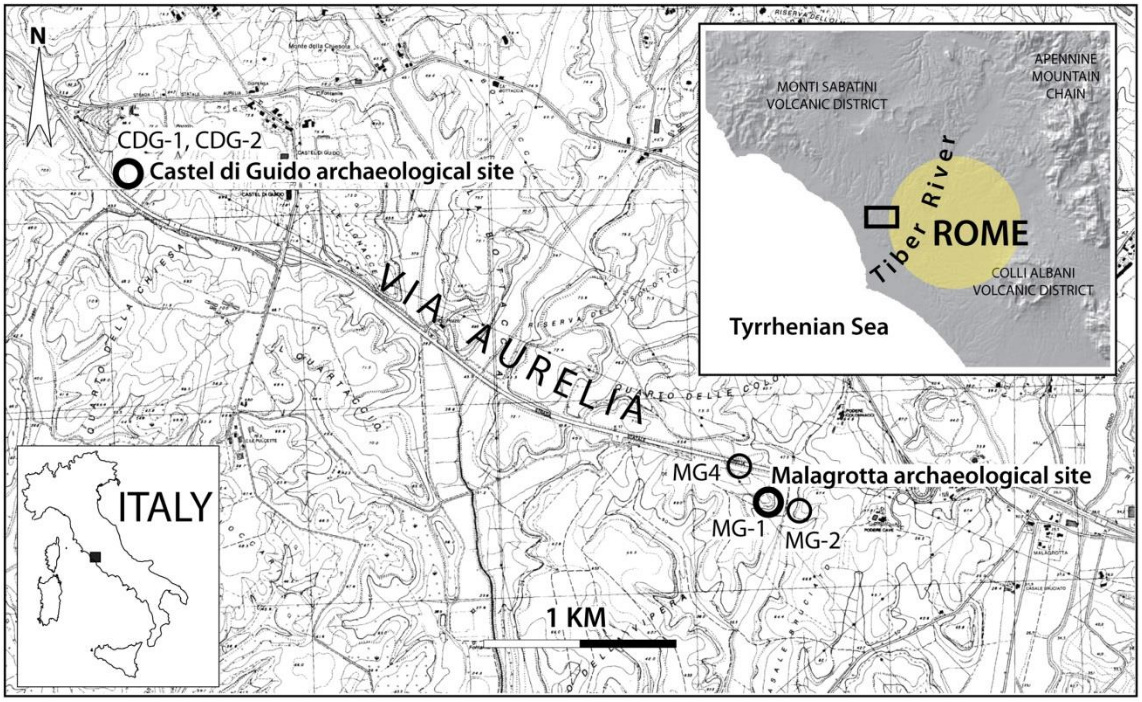

Fig S17. Topographic map of the investigated area (Carta tecnica regionale 10K formato TIFF, 1990-91, Regione Lazio, available under the Creative Commons Attribution license at: http://dati.lazio.it/weblist/cartografia/prodotti/1990_1991_CTR_10K_TIF/) showing location of the archaeological sites of Castel di Guido and Malagrotta and other sites (thinner circle lines) where the samples dated by $^{40}Ar/^{39}Ar$ method (yellow labels) were collected.

Digital Elevation Map in inset: TINITALY/01 square WA 6570, property of the Istituto Nazionale di Geofisica e Vulcanologia, Rome, http://tinitaly.pi.ingv.it/, used under permission.

Methods

Samples were analyzed at the Laboratoire des Sciences du Climat et de l'Environnement (CNRS-CEA, Gif-sur-Yvette), France, using procedures described in (*67*). Single grain measurements were done on potassic feldspars. Ages are calculated according the K total decay constant of (*68*) and the monitor flux standard ACs-2 dated to 1.1891 Ma (*69*). Procedural blanks were measured every two or three unknown samples. Mass discrimination was monitored by analysis of air pipette throughout the analytical period, and relative to a $^{40}Ar/^{36}Ar$ ratio of 298.56 (*70*). Results for each dated sample as well as irradiation duration (Oregon University, TRIGA reactor, CLICIT Facility) and J-values are presented as probability diagrams in Fig S18; full analytical data are provided in $^{40}Ar/^{39}Ar$ dataset.

Results

The weighted mean age for CDG-1 (394.8±2.6 ka, 2ϭ analytical uncertainties) is calculated from the three youngest crystals out of fifteen dated. The probability diagram is multimodal, showing that this volcaniclastic material is deeply reworked (Fig. S19).

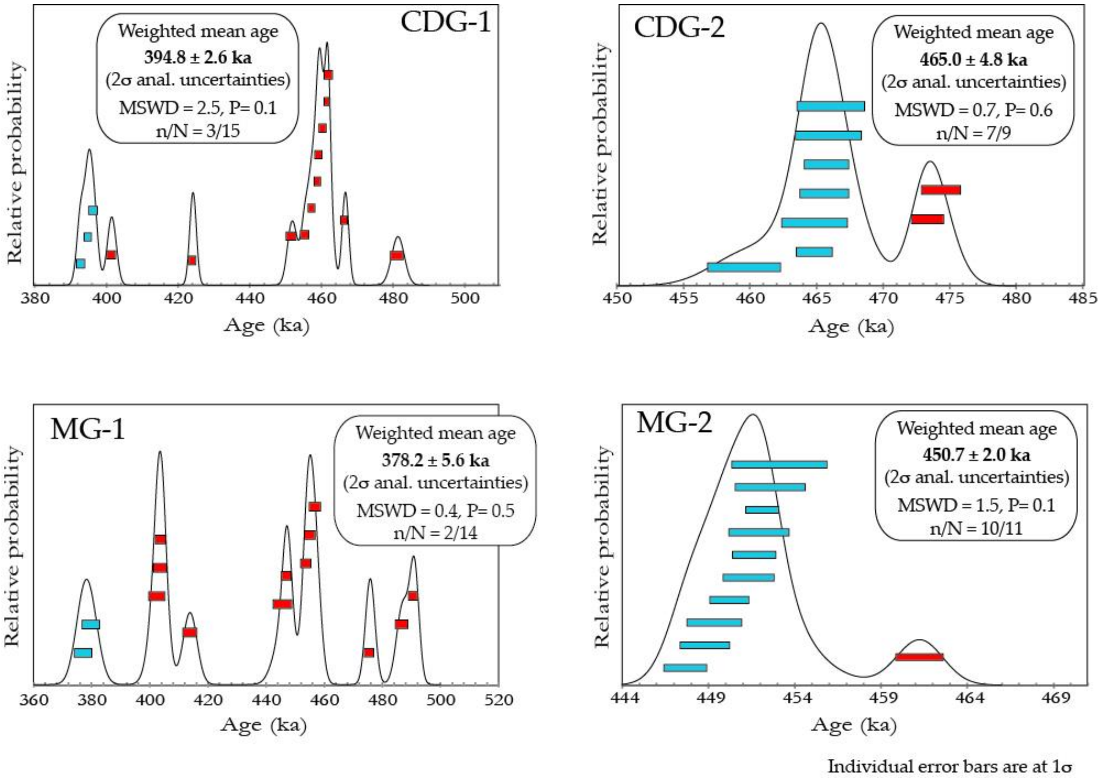

Fig S18 **-** Relative probability age plots for the samples dated by $^{40}Ar/^{39}Ar$ method in this work.

Sample CDG-2 is associated to a primary, fallout volcanic deposit. The weighted mean age of 465.0±4.8 ka (2ϭ analytical uncertainties) relies on seven of nine individually dated crystals. The scattered crystal age distribution provided by MG-1 demonstrates the reworked nature also of this sample, as only two crystals of the fourteen dated constitute the youngest population (378.2±5.6 ka, 2ϭ analytical uncertainties).

Sample MG-2 is associated to a primary volcanic, pyroclastic-flow deposit dated to 450.7±2.0 ka (10/11 dated crystals, 2ϭ analytical uncertainties).

Interpretations

Castel di Guido

Sample CDG-1 was collected in the reworked, matrix supported, volcaniclastic deposit above the paleosurface with lithic and bone artifacts (*21*) (Fig S19a). It yielded a youngest crystal population of 394.8 ±2.6 ka, consistent with previous inference about an age younger than that of the Vico α or Vico β eruption (414.8 ± 2.2 ka, 406.5 ± 2.4 ka *(67)*, based on the presence of reworked pumice of these geochemically very similar units, evidenced in *(13)*.

Moreover, based on the continued volcanic activity occurred at the Monti Sabatini Volcanic District in this time span (*71*), we assume that the age of the youngest crystal population provides a terminus ante-quem (maximum age) for the underlying paleosurface (see *(72)* for an in depth discussion). Such assumption is also supported by the lack in sample CDG-1 of the crystal populations of 378-381 ka occurring in two samples of the stratigraphically higher volcaniclastic layer (see Malagrotta section, Figure S19b).
Sample CDG-2 was collected in a primary pyroclastic deposit occurring ca. 1 m below the archaeological horizon (Figure S19a). It yielded a rather homogeneous crystal population providing a weighted mean age of 465.0±4.8 ka, consistent with its stratigraphic position below a volcanic layer, also underlying the archaeological paleosurface, which was petrographically identified (13) as a distal, partially reworked deposit of the 456±4 ka Pozzolane Rosse eruption unit from the Alban Hills Volcanic District (7*3*) (Figure S19a). Age, compositional features and stratigraphic position allow to attribute sample CDG-2 to the Monti Sabatini Fall C eruption unit (461±2 ka, *(74)*).

Malagrotta

Sample MG-1 yielded a youngest crystal population of 378.2 ±5.6 ka (Fig S18). The exact stratigraphic position within the Malagrotta sedimentary section of the fossil from which the dated volcanic material was extracted is unknown. However, this age is identical within uncertainties to that yielded by a sample collected from a volcaniclastic deposit (381±2 ka, sample MG4 in *(75*) cropping out at 62-65 m a.s.l. in Via Aurelia, ca. 400 m far from Malagrotta (Fig S17). Therefore, we infer that the bone from which the dated pyroclastic material was separated was incorporated within the petrographically equivalent volcaniclastic layer occurring ca. 60-62.5 m a.s.l. on top of the Malagrotta section, which was described in *(76*) (Fig. S19b').
Sample MG-2 was collected from a primary pyroclastic-flow deposit overlying a markedly erosive surface above sandy gravel deposits of the Santa Cecilia Formation (649-600 ka *(77*). Age and compositional features of the dated volcanic deposit allow us to attribute it to the Tufo Rosso a Scorie Nere eruption unit (452±2 ka *(74*).
This outcrop is located on the opposite stream valley bank and was described in *(26*), who attributed it to the same stratigraphic succession exposed at the Malagrotta archaeological site (Figure S19b"). Our chronostratigraphic investigations confirm that the pyroclastic-flow deposit occurs at the base of the same MIS 11 aggradational succession, hosting the artifacts recovered at the Malagrotta section described in *(76*).

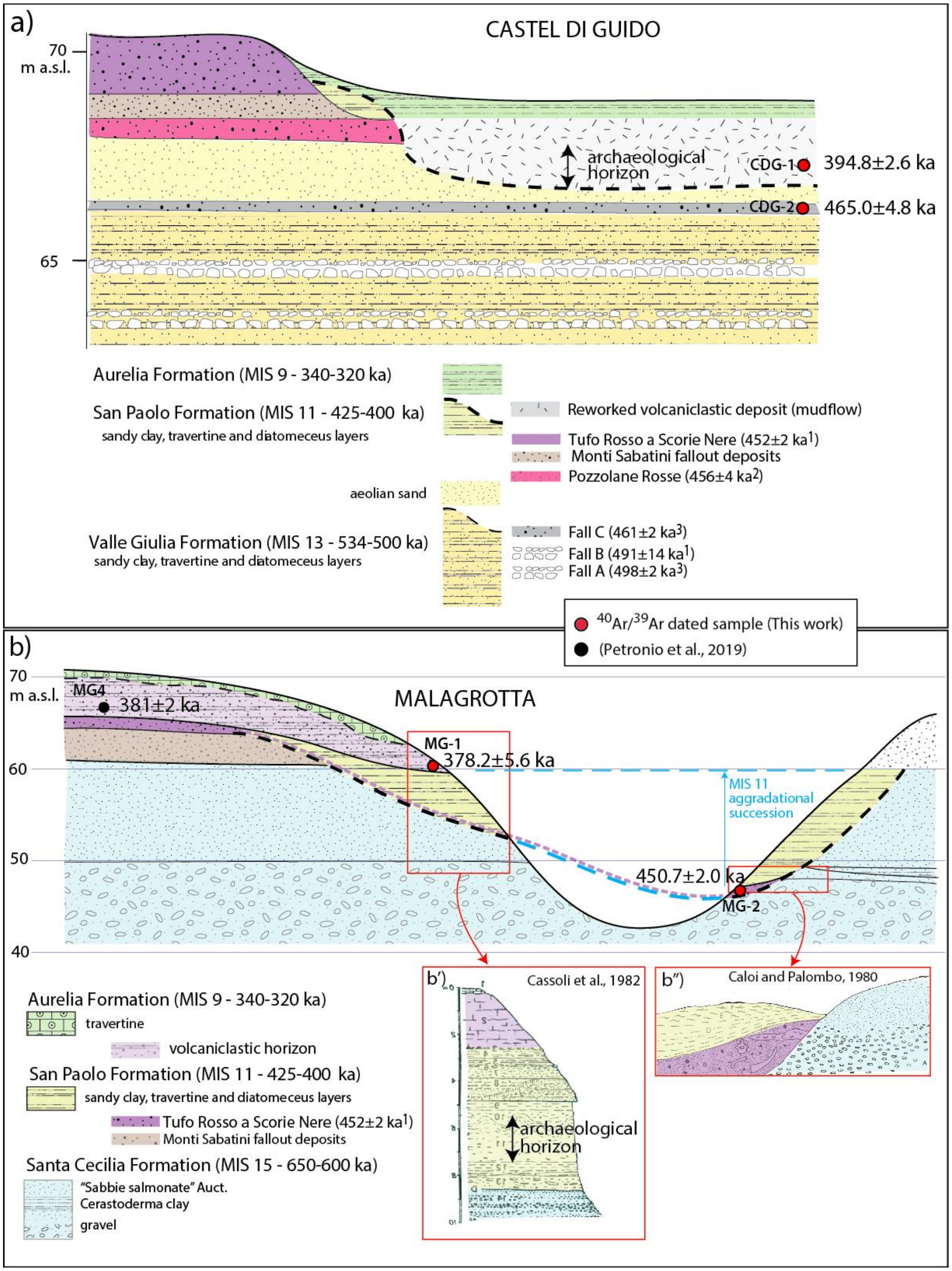

Fig S19 - Stratigraphic sketches of Castel di Guido (a) and Malagrotta (b) archaeological sites, showing position of the dated samples discussed in the present work. See text for explanations.
