# Supplementary material for: Elephant bones for the Middle Pleistocene toolmaker
Source: PLoS One. 2021 Aug 26;16(8):e0256090. doi: 10.1371/journal.pone.0256090 (PMC8389514; doi:10.1371/journal.pone.0256090)
Supplement: S3 File — (PDF) [file pone.0256090.s003.pdf]

Supporting Information

Elephant bones for the Middle Pleistocene toolmaker

Paola Villa*,Giovanni Boschian, Luca Pollarolo, Daniela Saccà, Fabrizio Marra, Sebastien Nomade, Alison Pereira.

Correspondence to: villap@colorado.edu

S3 File. $^{40}Ar/^{39}Ar$ dataset

$^{40}Ar/^{39}Ar$ dataset

Sebastien Nomade, Alison Pereira

**Instrument**	LSCE Micromass 5400		
**Standard:**	Alder Creek rhyolite sanidine		
**Standard age ± Ma:**	1.1891	± 0.0008	Niespolo et al., 2017
**Atmospheric argon ratios**			
$^{40}Ar/^{36}Ar$	298.56	± 0,31	Lee et al. (2006)
$^{38}Ar/^{36}Ar$	0.1885	± 0,0003	Lee et al. (2006)
**Decay constant (Renne et al., 2011)**			
λ_{40Ar}	$(0.5757 \pm 0.016) \times 10^{-10}\ a^{-1}$		
λ_{B}-	$(4.9548 \pm 0.0134) \times 10^{-10}\ a^{-1}$		
λ_{Tot}	$(5.531 \pm 0.0135) \times 10^{-10}\ a^{-1}$		

**Interferences Corrections**			
39/37(Ca)	±(%1σ)	6.5560E-04	1.36
38/37(Ca)	±(%1σ)	7.1800E-05	12.81
36/37(Ca)	±(%1σ)	2.6630E-04	1.5
40/39(K)	±(%1σ)	3.8230E-03	2.67
38/39(K)	±(%1σ)	1.2031E-02	0.16
36/38(Cl)	±(%1σ)	262.8	1.71
K/Ca	±(%1σ)	0.43	0
K/Cl	±(%1σ)	1.17	0

**Sample ID:**	**CDG-1**	Lab#N1572-01/N1572-15	J = 0.00052990 ± 0.00000106		
Sanidines			Irradiation #	reactor:	CICLIT, Oregon University, Triga reactor
Flux standard monitor	ACs-2	1.1891 Ma	CO-001 (120 min)	Single crystal total fusion	

N	^{40}Ar (moles)	^{36}Ar V	$\pm\sigma_{36}$ V	^{37}Ar V	$\pm\sigma_{37}$ V	^{38}Ar V	$\pm\sigma_{38}$ V	^{39}Ar V	$\pm\sigma_{39}$ V	^{40}Ar V	$\pm\sigma_{40}$ V	D(1)	$\pm\%s_D$	$\%^{40}$Ar*	Age (ka)	±σ	K/Ca	±σ
**N1572-01**	**2.96E-15**	**6.97E-08**	**19.67**	**7.65E-05**	**1.27**	**6.32E-05**	**0.35**	**5.16E-03**	**0.13**	**2.16E-03**	**0.09**	**1.01**	**0.10**	**98.41**	**394.97**	**0.99**	**29.03**	**0.37**
**N1572-02**	**6.12E-15**	**2.03E-06**	**0.95**	**1.20E-04**	**0.96**	**9.95E-05**	**0.28**	**8.04E-03**	**0.11**	**4.47E-03**	**0.07**	**1.01**	**0.10**	**85.95**	**457.47**	**0.97**	**28.78**	**0.28**
**N1572-03**	**1.08E-14**	**1.20E-06**	**1.78**	**2.26E-04**	**0.59**	**1.92E-04**	**0.26**	**1.55E-02**	**0.10**	**7.87E-03**	**0.07**	**1.01**	**0.10**	**94.93**	**461.71**	**0.72**	**29.50**	**0.18**
**N1572-04**	**4.26E-15**	**2.34E-07**	**7.35**	**9.29E-05**	**0.99**	**7.51E-05**	**0.31**	**6.29E-03**	**0.12**	**3.11E-03**	**0.09**	**1.01**	**0.10**	**97.22**	**460.55**	**1.06**	**29.12**	**0.29**
**N1572-07**	**5.38E-15**	**2.63E-07**	**7.93**	**1.21E-04**	**0.83**	**9.81E-05**	**0.32**	**7.98E-03**	**0.11**	**3.93E-03**	**0.08**	**1.01**	**0.10**	**97.47**	**459.39**	**0.98**	**28.32**	**0.24**
**N1572-08**	**4.17E-15**	**1.02E-06**	**2.07**	**1.51E-04**	**0.87**	**8.01E-05**	**0.43**	**6.59E-03**	**0.11**	**3.04E-03**	**0.10**	**1.01**	**0.10**	**89.57**	**396.44**	**1.12**	**18.73**	**0.16**
**N1572-09**	**7.26E-15**	**1.50E-06**	**2.15**	**1.43E-04**	**0.70**	**1.24E-04**	**0.23**	**1.00E-02**	**0.11**	**5.30E-03**	**0.07**	**1.01**	**0.10**	**91.05**	**462.23**	**1.12**	**30.06**	**0.21**
**N1572-10**	**5.51E-15**	**2.16E-07**	**9.18**	**1.17E-04**	**0.84**	**9.68E-05**	**0.36**	**8.07E-03**	**0.11**	**4.02E-03**	**0.05**	**1.01**	**0.10**	**97.86**	**466.64**	**0.92**	**29.55**	**0.25**
**N1572-11**	**3.41E-15**	**9.52E-07**	**1.93**	**7.28E-05**	**1.02**	**6.52E-05**	**0.36**	**5.34E-03**	**0.11**	**2.49E-03**	**0.05**	**1.01**	**0.10**	**87.99**	**392.93**	**1.12**	**31.51**	**0.32**
**N1572-12**	**4.00E-15**	**6.72E-07**	**1.79**	**8.30E-05**	**1.02**	**6.87E-05**	**0.32**	**5.65E-03**	**0.12**	**2.92E-03**	**0.09**	**1.01**	**0.10**	**92.62**	**459.17**	**0.94**	**29.23**	**0.30**
**N1572-13**	**3.73E-15**	**9.96E-07**	**2.04**	**8.65E-05**	**1.60**	**6.99E-05**	**0.41**	**5.76E-03**	**0.12**	**2.72E-03**	**0.12**	**1.01**	**0.10**	**88.53**	**401.48**	**1.26**	**28.63**	**0.46**
**N1572-14**	**4.44E-15**	**1.12E-06**	**1.58**	**9.03E-05**	**1.39**	**7.46E-05**	**0.46**	**6.07E-03**	**0.12**	**3.24E-03**	**0.11**	**1.01**	**0.10**	**89.14**	**455.54**	**1.16**	**28.91**	**0.40**
**N1572-15**	**2.60E-15**	**2.33E-07**	**6.58**	**6.58E-05**	**1.70**	**4.68E-05**	**0.59**	**3.86E-03**	**0.12**	**1.90E-03**	**0.09**	**1.01**	**0.10**	**95.83**	**451.82**	**1.34**	**25.19**	**0.43**

Results	^{40}Ar*/^{39}Ark ± 1σ	Age ± 1σ (ka)	MSWD	^{39}Ar(k) (%, n)	K/Ca ± 1σ
Weighted mean	0.4121 ± 0.0010	394.8 ± 1.3	2.46	15.79	22.4 ± 3.9
	± 0.25%	± 0.32%		3	
	Full External Error	± 1.5	1.21		
	Analytical Error	± 1.0	1.5698		

Results	40(a)/36(a) ± 1σ	40(r)/39(k) ± 1σ	Age ± 1σ (ka)	MSWD
Inverse Isochron	294.60 ± 18.8	0.4125 ± 0.0024	395.2 ± 2.4	4.83
	± 6.36%	± 0.58%	± 0.61%	
Full External Error			± 2.5	
Analytical Error			± 0.6	

Statistics			
Statistical F ratio	0.87	Convergence	0.0000509398
Error Magnification	2.1979	Number of Iterations	3
Number of Data Points	3	Calculated Line	Weighted York-2

Background corrections CDG-1

N	^{36}Ar V	±s36 V	^{37}Ar V	±s37 V	^{38}Ar V	±s38 V	^{39}Ar V	±s39 V	^{40}Ar V	±s40 V
**N1572-01**	**2.27E-07**	**8.64E-09**	**2.49E-07**	**1.72E-08**	**5.86E-08**	**1.25E-08**	**6.57E-07**	**1.14E-07**	**1.68E-05**	**2.86E-07**
**N1572-02**	**2.27E-07**	**8.64E-09**	**2.49E-07**	**1.72E-08**	**5.86E-08**	**1.25E-08**	**6.57E-07**	**1.14E-07**	**1.68E-05**	**2.86E-07**
**N1572-03**	**2.20E-07**	**1.50E-08**	**2.40E-07**	**2.11E-08**	**8.34E-08**	**1.06E-08**	**1.37E-06**	**2.16E-07**	**1.87E-05**	**6.74E-07**
**N1572-04**	**2.20E-07**	**1.50E-08**	**2.40E-07**	**2.11E-08**	**8.34E-08**	**1.06E-08**	**1.37E-06**	**2.16E-07**	**1.87E-05**	**6.74E-07**
**N1572-05**	**2.03E-07**	**9.72E-09**	**2.57E-07**	**1.85E-08**	**1.08E-07**	**2.25E-08**	**5.56E-06**	**8.90E-08**	**1.87E-05**	**6.74E-07**
**N1572-06**	**2.03E-07**	**9.72E-09**	**2.57E-07**	**1.85E-08**	**1.08E-07**	**2.25E-08**	**5.56E-06**	**8.90E-08**	**2.05E-05**	**2.66E-07**
**N1572-07**	**2.03E-07**	**9.72E-09**	**2.57E-07**	**1.85E-08**	**1.08E-07**	**2.25E-08**	**5.56E-06**	**8.90E-08**	**2.05E-05**	**2.66E-07**
**N1572-08**	**2.03E-07**	**9.72E-09**	**2.57E-07**	**1.85E-08**	**1.08E-07**	**2.25E-08**	**5.56E-06**	**8.90E-08**	**2.05E-05**	**2.66E-07**
**N1572-09**	**2.13E-07**	**1.66E-08**	**2.65E-07**	**1.06E-08**	**5.65E-08**	**1.92E-08**	**4.57E-07**	**1.10E-07**	**1.81E-05**	**5.24E-07**
**N1572-10**	**2.13E-07**	**1.66E-08**	**2.65E-07**	**1.06E-08**	**5.65E-08**	**1.92E-08**	**4.57E-07**	**1.10E-07**	**1.81E-05**	**5.24E-07**
**N1572-11**	**1.76E-07**	**6.34E-09**	**2.02E-07**	**1.98E-08**	**6.88E-08**	**6.26E-09**	**9.90E-07**	**1.73E-07**	**1.95E-05**	**5.06E-07**
**N1572-12**	**1.76E-07**	**6.34E-09**	**2.02E-07**	**1.98E-08**	**6.88E-08**	**6.26E-09**	**9.90E-07**	**1.73E-07**	**1.95E-05**	**5.06E-07**
**N1572-13**	**1.55E-07**	**1.49E-08**	**2.37E-07**	**1.21E-08**	**1.05E-07**	**2.16E-08**	**9.75E-07**	**1.68E-07**	**1.81E-05**	**5.26E-07**
**N1572-14**	**1.55E-07**	**1.49E-08**	**2.37E-07**	**1.21E-08**	**1.05E-07**	**2.16E-08**	**9.75E-07**	**1.68E-07**	**1.81E-05**	**5.26E-07**
**N1572-15**	**1.38E-07**	**1.05E-08**	**1.50E-07**	**1.12E-08**	**4.79E-08**	**1.40E-08**	**2.44E-07**	**2.02E-07**	**1.62E-05**	**4.05E-07**

Table S4

**Sample ID:**	**CDG-2**	Lab#N1578-01/N1578-09				J = 0.00052907 ± 0.00000265	
Sanidines			Irradiation #		reactor:		CICLIT, Oregon University, Triga reactor
Flux standard monitor	ACs-2	1.1891 Ma	CO-001 (120 min)	Single crystal total fusion			

N	^{40}Ar (moles)	^{36}Ar V	$\pm\sigma_{36}$ V	^{37}Ar V	$\pm\sigma_{37}$ V	^{38}Ar V	$\pm\sigma_{38}$ V	^{39}Ar V	$\pm\sigma_{39}$ V	^{40}Ar V	$\pm\sigma_{40}$ V	D[(1)]	$\pm\%s_D$	%^{40}Ar*	Age (ka)	±σ	K/Ca	±σ
**N1578-01**	**3.34E-15**	**2.30E-06**	**1.23**	**6.82E-05**	**1.53**	**4.38E-05**	**0.36**	**3.58E-03**	**0.13**	**2.44E-03**	**0.08**	**1.01**	**0.10**	71.50	466.13	2.47	22.55	0.35
**N1578-02**	**4.63E-15**	**2.47E-06**	**1.07**	**1.00E-04**	**1.11**	**6.59E-05**	**0.47**	**5.40E-03**	**0.12**	**3.38E-03**	**0.08**	**1.01**	**0.10**	77.83	465.80	1.63	23.21	0.26
**N1578-03**	**3.87E-15**	**3.65E-07**	**5.50**	**1.04E-04**	**1.45**	**6.66E-05**	**0.41**	**5.45E-03**	**0.12**	**2.82E-03**	**0.15**	**1.01**	**0.10**	95.70	474.19	1.41	22.45	0.33
**N1578-04**	**1.72E-15**	**8.58E-08**	**22.97**	**4.73E-05**	**2.37**	**2.98E-05**	**0.61**	**2.52E-03**	**0.14**	**1.26E-03**	**0.14**	**1.01**	**0.10**	97.50	465.94	2.42	22.91	0.54
**N1578-05**	**4.01E-15**	**6.51E-07**	**2.92**	**1.03E-04**	**0.78**	**6.62E-05**	**0.42**	**5.49E-03**	**0.11**	**2.92E-03**	**0.06**	**1.01**	**0.10**	92.92	473.20	1.17	22.85	0.18
**N1578-06**	**4.72E-15**	**1.04E-06**	**2.43**	**1.31E-04**	**1.29**	**7.88E-05**	**0.42**	**6.42E-03**	**0.12**	**3.45E-03**	**0.07**	**1.01**	**0.10**	90.55	464.90	1.31	21.03	0.27
**N1578-07**	**2.24E-15**	**1.42E-07**	**13.24**	**5.44E-05**	**1.59**	**3.90E-05**	**0.40**	**3.25E-03**	**0.12**	**1.63E-03**	**0.07**	**1.01**	**0.10**	96.91	465.63	1.78	25.74	0.41
**N1578-08**	**1.82E-15**	**1.65E-07**	**12.42**	**5.23E-05**	**1.49**	**3.22E-05**	**0.86**	**2.63E-03**	**0.13**	**1.33E-03**	**0.13**	**1.01**	**0.10**	95.86	464.93	2.41	21.61	0.32
**N1578-09**	**1.72E-15**	**2.17E-07**	**9.60**	**4.75E-05**	**1.10**	**3.01E-05**	**0.52**	**2.46E-03**	**0.13**	**1.25E-03**	**0.19**	**1.01**	**0.10**	94.39	459.75	2.65	22.27	0.25

Results	^{40}Ar*/^{39}Ark ± 1σ	Age ± 1σ (ka)	MSWD	^{39}Ar(k) (%, n)	K/Ca ± 1σ
Weighted mean	0.4862 ± 0.0008	465.0 ± 2.4	0.77	0.44	22.5 ± 0.5
	± 0.16%	± 0.52%		7	
	Full External Error	± 2.6	1.01		
	Analytical Error	± 0.7	1.0000		

Results	40(a)/36(a) ± 1σ	40(r)/39(k) ± 1σ	Age ± 1σ (ka)	MSWD
Inverse Isochron	301.38 ± 3.8	0.4856 ± 0.0011	464.5 ± 2.6	0.82
	± 1.25%	± 0.23%	± 0.55%	
Full External Error			± 2.7	
Analytical Error			± 1.1	

Statistics				
Statistical F ratio	2.07	Convergence	0.0056717462
Error Magnification	1.2084	Number of Iterations	2
Number of Data Points	9	Calculated Line	Weighted York-2

Background corrections CDG-2

N	^{36}Ar V	±s36 V	^{37}Ar V	±s37 V	^{38}Ar V	±s38 V	^{39}Ar V	±s39 V	^{40}Ar V	±s40 V
**N1578-01**	**1.43E-07**	**1.62E-08**	**8.86E-08**	**2.48E-08**	**7.82E-08**	**2.18E-08**	**3.63E-07**	**1.45E-07**	**2.45E-05**	**2.45E-07**
**N1578-02**	**1.43E-07**	**1.62E-08**	**8.86E-08**	**2.48E-08**	**7.82E-08**	**2.18E-08**	**3.63E-07**	**1.45E-07**	**2.45E-05**	**2.45E-07**
**N1578-03**	**1.77E-07**	**1.71E-08**	**1.18E-07**	**2.04E-08**	**8.99E-08**	**3.11E-08**	**2.90E-06**	**1.45E-07**	**2.44E-05**	**5.19E-07**
**N1578-04**	**1.77E-07**	**1.71E-08**	**1.18E-07**	**2.04E-08**	**8.99E-08**	**3.11E-08**	**2.90E-06**	**1.45E-07**	**2.44E-05**	**5.19E-07**
**N1578-05**	**1.77E-07**	**1.71E-08**	**1.18E-07**	**2.04E-08**	**8.99E-08**	**3.11E-08**	**2.90E-06**	**1.45E-07**	**2.44E-05**	**5.19E-07**
**N1578-06**	**1.67E-07**	**1.62E-08**	**1.12E-07**	**2.95E-08**	**4.55E-08**	**1.92E-08**	**3.97E-07**	**2.41E-07**	**2.14E-05**	**2.78E-07**
**N1578-07**	**1.67E-07**	**1.62E-08**	**1.12E-07**	**2.95E-08**	**4.55E-08**	**1.92E-08**	**3.97E-07**	**2.41E-07**	**2.14E-05**	**2.78E-07**
**N1578-08**	**1.68E-07**	**1.92E-08**	**2.18E-07**	**2.53E-08**	**7.93E-08**	**1.87E-08**	**3.00E-07**	**2.59E-07**	**2.03E-05**	**3.65E-07**
**N1578-09**	**1.68E-07**	**1.92E-08**	**2.18E-07**	**2.53E-08**	**7.93E-08**	**1.87E-08**	**3.00E-07**	**2.59E-07**	**2.03E-05**	**3.65E-07**

Table S5

**Sample ID:**	**MG-1**	Lab#N1577-01/N1577-14		J = 0.00052803 ± 0.00000264	
Sanidines			Irradiation #	reactor:	CICLIT, Oregon University, Triga reactor
Flux standard monitor	ACs-2	1.1891 Ma	CO-001 (120 min)	Single crystal total fusion	

N	^{40}Ar (moles)	^{36}Ar V	$\pm\sigma_{36}$ V	^{37}Ar V	$\pm\sigma_{37}$ V	^{38}Ar V	$\pm\sigma_{38}$ V	^{39}Ar V	$\pm\sigma_{39}$ V	^{40}Ar V	$\pm\sigma_{40}$ V	D(1)	$\pm\%s_D$	$\%^{40}Ar^*$	Age (ka)	±σ	K/Ca	±σ
**N1577-01**	**3.29E-15**	**1.00E-06**	**1.54**	**7.00E-05**	**1.72**	**5.19E-05**	**0.42**	**4.20E-03**	**0.17**	**2.40E-03**	**0.17**	**1.01**	**0.10**	**87.11**	**475.78**	**1.64**	**25.80**	**0.45**
**N1577-02**	**2.40E-15**	**4.87E-07**	**3.41**	**5.19E-05**	**1.21**	**4.12E-05**	**0.47**	**3.37E-03**	**0.13**	**1.75E-03**	**0.14**	**1.01**	**0.10**	**91.22**	**453.97**	**1.70**	**27.87**	**0.34**
**N1577-03**	**2.15E-15**	**3.85E-07**	**4.86**	**6.01E-05**	**1.58**	**4.19E-05**	**0.51**	**3.43E-03**	**0.13**	**1.57E-03**	**0.13**	**1.01**	**0.10**	**92.16**	**403.69**	**1.75**	**24.53**	**0.39**
**N1577-04**	**2.12E-15**	**1.06E-06**	**2.15**	**3.93E-05**	**2.06**	**3.48E-05**	**0.50**	**2.83E-03**	**0.13**	**1.55E-03**	**0.08**	**1.01**	**0.10**	**79.18**	**413.77**	**2.42**	**31.01**	**0.64**
**N1577-05**	**2.47E-15**	**7.01E-07**	**2.72**	**4.67E-05**	**4.45**	**4.01E-05**	**0.49**	**3.32E-03**	**0.14**	**1.81E-03**	**0.15**	**1.01**	**0.10**	**87.92**	**457.04**	**1.92**	**30.58**	**1.36**
**N1577-06**	**1.53E-15**	**7.21E-07**	**2.69**	**2.93E-05**	**2.36**	**2.57E-05**	**0.52**	**2.13E-03**	**0.14**	**1.12E-03**	**0.14**	**1.01**	**0.10**	**80.21**	**402.35**	**2.77**	**31.23**	**0.74**
**N1577-07**	**5.97E-15**	**5.14E-06**	**0.66**	**7.81E-05**	**1.66**	**6.65E-05**	**0.27**	**5.50E-03**	**0.12**	**4.35E-03**	**0.10**	**1.01**	**0.10**	**64.43**	**487.02**	**2.18**	**30.28**	**0.50**
**N1577-08**	**1.61E-15**	**3.22E-07**	**5.88**	**3.12E-05**	**1.06**	**3.12E-05**	**0.60**	**2.54E-03**	**0.13**	**1.18E-03**	**0.14**	**1.01**	**0.10**	**91.23**	**403.54**	**2.27**	**34.97**	**0.38**
**N1577-09**	**2.50E-15**	**6.37E-07**	**3.17**	**5.40E-05**	**1.78**	**4.14E-05**	**0.45**	**3.41E-03**	**0.13**	**1.83E-03**	**0.10**	**1.01**	**0.10**	**89.11**	**455.34**	**1.87**	**27.18**	**0.49**
**N1577-10**	**8.65E-16**	**3.03E-07**	**4.36**	**2.38E-05**	**2.31**	**1.65E-05**	**0.84**	**1.36E-03**	**0.16**	**6.32E-04**	**0.17**	**1.01**	**0.10**	**85.15**	**376.89**	**2.93**	**24.58**	**0.57**
**N1577-11**	**1.33E-15**	**6.31E-07**	**2.72**	**1.97E-05**	**3.91**	**1.99E-05**	**0.65**	**1.66E-03**	**0.17**	**9.68E-04**	**0.16**	**1.01**	**0.10**	**80.03**	**445.95**	**3.19**	**36.16**	**1.42**
**N1577-12**	**9.34E-16**	**8.97E-08**	**18.67**	**4.05E-05**	**2.84**	**1.99E-05**	**0.63**	**1.64E-03**	**0.13**	**6.82E-04**	**0.16**	**1.01**	**0.10**	**95.63**	**379.61**	**3.02**	**17.42**	**0.49**
**N1577-13**	**4.51E-15**	**2.07E-06**	**1.17**	**7.59E-05**	**1.54**	**6.32E-05**	**0.35**	**5.18E-03**	**0.12**	**3.30E-03**	**0.07**	**1.01**	**0.10**	**80.82**	**491.03**	**1.56**	**29.32**	**0.45**
**N1577-14**	**1.98E-15**	**5.31E-08**	**27.56**	**4.30E-05**	**2.50**	**3.65E-05**	**0.46**	**3.03E-03**	**0.13**	**1.44E-03**	**0.12**	**1.01**	**0.10**	**98.34**	**447.26**	**1.61**	**30.31**	**0.76**

Results		$^{40}Ar^*/^{39}Ark \pm 1\sigma$	Age ± 1σ (ka)	MSWD	$^{39}Ar(k)$ (%, n)	K/Ca ± 1σ
Weighted mean		0.3962 ± 0.0022	378.2 ± 2.8	0.42	0.29	20.5 ± 3.5
		± 0.56%	± 0.75%		2	
	Full External Error		± 2.9	1.63		
	Analytical Error		± 2.1	1.0000		

Background corrections MG-1

N	^{36}Ar V	±s36 V	^{37}Ar V	±s37 V	^{38}Ar V	±s38 V	^{39}Ar V	±s39 V	^{40}Ar V	±s40 V
**N1577-01**	**2.48E-07**	**1.14E-08**	**2.18E-07**	**2.53E-08**	**7.11E-08**	**2.58E-08**	**5.52E-07**	**1.85E-07**	**2.55E-05**	**2.80E-07**
**N1577-02**	**2.48E-07**	**1.14E-08**	**2.18E-07**	**2.53E-08**	**7.11E-08**	**2.58E-08**	**5.52E-07**	**1.85E-07**	**2.55E-05**	**2.80E-07**
**N1577-03**	**2.48E-07**	**1.14E-08**	**2.18E-07**	**2.53E-08**	**7.11E-08**	**2.58E-08**	**5.52E-07**	**1.85E-07**	**2.55E-05**	**2.80E-07**
**N1577-04**	**2.38E-07**	**1.45E-08**	**2.07E-07**	**2.19E-08**	**7.94E-08**	**1.88E-08**	**3.96E-07**	**1.55E-07**	**2.65E-05**	**5.56E-07**
**N1577-05**	**2.38E-07**	**1.45E-08**	**2.07E-07**	**2.19E-08**	**7.94E-08**	**1.88E-08**	**3.96E-07**	**1.55E-07**	**2.65E-05**	**5.56E-07**
**N1577-06**	**2.25E-07**	**1.46E-08**	**2.59E-07**	**9.57E-09**	**4.76E-08**	**1.65E-08**	**3.80E-07**	**6.39E-08**	**2.34E-05**	**2.80E-07**
**N1577-07**	**2.25E-07**	**1.46E-08**	**2.59E-07**	**9.57E-09**	**4.76E-08**	**1.65E-08**	**3.80E-07**	**6.39E-08**	**2.34E-05**	**2.80E-07**
**N1577-08**	**2.15E-07**	**1.36E-08**	**1.43E-07**	**1.54E-08**	**1.57E-07**	**1.50E-08**	**5.86E-07**	**2.72E-07**	**2.71E-05**	**3.26E-07**
**N1577-09**	**2.15E-07**	**1.36E-08**	**1.43E-07**	**1.54E-08**	**1.57E-07**	**1.50E-08**	**5.86E-07**	**2.72E-07**	**2.71E-05**	**3.26E-07**
**N1577-10**	**2.00E-07**	**8.00E-09**	**2.50E-07**	**1.25E-08**	**8.00E-08**	**1.60E-08**	**4.00E-07**	**1.60E-07**	**1.81E-05**	**3.89E-07**
**N1577-11**	**2.00E-07**	**8.00E-09**	**2.50E-07**	**1.25E-08**	**8.00E-08**	**1.60E-08**	**4.00E-07**	**1.60E-07**	**1.81E-05**	**3.89E-07**
**N1577-12**	**2.02E-07**	**1.27E-08**	**1.73E-07**	**3.22E-08**	**5.62E-08**	**1.69E-08**	**3.77E-07**	**1.67E-07**	**1.77E-05**	**3.83E-07**
**N1577-13**	**2.02E-07**	**1.27E-08**	**1.73E-07**	**3.22E-08**	**5.62E-08**	**1.69E-08**	**3.77E-07**	**1.67E-07**	**1.77E-05**	**3.83E-07**
**N1577-14**	**2.02E-07**	**1.27E-08**	**1.73E-07**	**3.22E-08**	**5.62E-08**	**1.69E-08**	**3.77E-07**	**1.67E-07**	**1.77E-05**	**3.83E-07**

Table S6

Sample ID:	MG-2	Lab#N1651-01/N1651-11	J = 0.00053080 ± 0.00000096		
Sanidines			Irradiation # CO-001 (120 min)	reactor:	CICLIT, Oregon University, Triga reactor
Flux standard monitor	ACs-2	1.1891 Ma		Single crystal total fusion	

N	^{40}Ar (moles)	^{36}Ar V	$\pm\sigma_{36}$ V	^{37}Ar V	$\pm\sigma_{37}$ V	^{38}Ar V	$\pm\sigma_{38}$ V	^{39}Ar V	$\pm\sigma_{39}$ V	^{40}Ar V	$\pm\sigma_{40}$ V	D(1)	$\pm\%s_D$	$\%^{40}Ar^*$	Age (ka)	±σ	K/Ca	±σ
**N1651-01**	**5.87E-15**	**1.59E-07**	**8.63**	**5.97E-05**	**3.80**	**4.65E-05**	**0.44**	**3.70E-03**	**0.12**	**1.83E-03**	**0.13**	**1.01**	**0.09**	**96.91**	**461.25**	**1.34**	**26.63**	**1.01**
**N1651-02**	**6.33E-15**	**1.70E-07**	**8.45**	**5.83E-05**	**3.97**	**4.96E-05**	**0.49**	**4.11E-03**	**0.11**	**1.98E-03**	**0.10**	**1.01**	**0.09**	**96.87**	**447.74**	**1.23**	**30.29**	**1.20**
**N1651-03**	**1.91E-15**	**5.92E-08**	**18.68**	**3.06E-05**	**7.28**	**1.54E-05**	**0.76**	**1.22E-03**	**0.13**	**5.98E-04**	**0.14**	**1.00**	**0.09**	**96.68**	**453.17**	**2.74**	**17.19**	**1.25**
**N1651-04**	**3.78E-15**	**8.87E-08**	**17.55**	**3.90E-05**	**6.05**	**2.95E-05**	**0.57**	**2.44E-03**	**0.13**	**1.18E-03**	**0.14**	**1.00**	**0.09**	**97.23**	**452.65**	**2.03**	**26.84**	**1.63**
**N1651-05**	**3.56E-15**	**7.02E-08**	**10.59**	**3.56E-05**	**7.06**	**2.77E-05**	**0.36**	**2.32E-03**	**0.11**	**1.11E-03**	**0.09**	**1.00**	**0.09**	**97.58**	**450.29**	**1.12**	**27.95**	**1.97**
**N1651-06**	**3.51E-15**	**2.97E-07**	**3.05**	**3.00E-05**	**5.46**	**2.56E-05**	**0.51**	**2.13E-03**	**0.14**	**1.10E-03**	**0.11**	**1.00**	**0.09**	**91.38**	**451.40**	**1.48**	**30.53**	**1.67**
**N1651-07**	**3.04E-15**	**1.17E-07**	**8.79**	**3.42E-05**	**5.75**	**2.37E-05**	**0.51**	**1.93E-03**	**0.12**	**9.51E-04**	**0.13**	**1.00**	**0.09**	**95.83**	**451.99**	**1.74**	**24.32**	**1.40**
**N1651-08**	**4.69E-15**	**1.42E-07**	**4.91**	**5.46E-05**	**5.12**	**3.64E-05**	**0.57**	**3.01E-03**	**0.11**	**1.47E-03**	**0.08**	**1.01**	**0.09**	**96.62**	**452.18**	**0.94**	**23.67**	**1.21**
**N1651-09**	**4.19E-15**	**1.07E-07**	**8.22**	**3.78E-05**	**8.18**	**3.30E-05**	**0.35**	**2.70E-03**	**0.14**	**1.31E-03**	**0.11**	**1.00**	**0.09**	**97.01**	**451.72**	**1.26**	**30.68**	**2.51**
**N1651-10**	**6.65E-15**	**8.80E-07**	**2.03**	**6.03E-05**	**3.40**	**4.76E-05**	**0.39**	**3.85E-03**	**0.13**	**2.08E-03**	**0.11**	**1.01**	**0.09**	**86.87**	**449.43**	**1.56**	**27.47**	**0.93**
**N1651-11**	**6.57E-15**	**7.47E-07**	**2.23**	**5.41E-05**	**4.05**	**4.70E-05**	**0.45**	**3.89E-03**	**0.11**	**2.05E-03**	**0.09**	**1.01**	**0.09**	**88.62**	**448.88**	**1.42**	**30.91**	**1.25**

Results		$^{40}Ar^*/^{39}Ark \pm 1\sigma$	Age ± 1σ (ka)	MSWD	$^{39}Ar(k)$ (%, n)	K/Ca ± 1σ
Weighted mean		0.4697 ± 0.0006	450.7 ± 1.0	1.54	2.84	26.5 ± 1.4
		± 0.12%	± 0.22%	13.00	10	
	Full External Error		± 1.3	1.47		
	Analytical Error		± 0.5	1.2421		

Results	40(a)/36(a) ± 1σ	40(r)/39(k) ± 1σ	Age ± 1σ (ka)	MSWD
Inverse Isochron	291.07 ± 8.5	0.4703 ± 0.0009	451.3 ± 1.2	1.59
	± 2.92%	± 0.18%	± 0.26%	
Full External Error			± 1.4	
Analytical Error			± 0.8	

Statistics				
	Statistical F ratio	1.50	Convergence	0.0003894810
	Error Magnification	1.2614	Number of Iterations	3
	Number of Data Points	10	Calculated Line	Weighted York-2

Background corrections MG-2

N	^{36}Ar V	±s36 V	^{37}Ar V	±s37 V	^{38}Ar V	±s38 V	^{39}Ar V	±s39 V	^{40}Ar V	±s40 V
**N1651-01**	**1.07E-07**	**1.33E-08**	**4.99E-08**	**1.21E-08**	**2.24E-08**	**2.14E-08**	**7.14E-08**	**7.06E-08**	**2.67E-05**	**5.34E-07**
**N1651-02**	**1.07E-07**	**1.33E-08**	**4.99E-08**	**1.21E-08**	**2.24E-08**	**1.47E-08**	**7.14E-08**	**7.06E-08**	**2.67E-05**	**5.34E-07**
**N1651-03**	**1.19E-07**	**9.67E-09**	**3.33E-08**	**1.46E-08**	**3.35E-08**	**1.18E-08**	**1.19E-06**	**1.12E-07**	**2.83E-05**	**3.68E-07**
**N1651-04**	**1.19E-07**	**9.67E-09**	**3.33E-08**	**1.46E-08**	**3.35E-08**	**1.18E-08**	**1.19E-06**	**1.12E-07**	**2.83E-05**	**3.68E-07**
**N1651-05**	**1.25E-07**	**5.61E-09**	**3.33E-08**	**9.50E-11**	**4.08E-08**	**1.29E-08**	**3.11E-07**	**1.50E-07**	**2.50E-05**	**3.24E-07**
**N1651-06**	**1.25E-07**	**5.61E-09**	**4.44E-08**	**1.27E-10**	**4.08E-08**	**1.29E-08**	**3.11E-07**	**1.50E-07**	**2.50E-05**	**3.24E-07**
**N1651-07**	**1.40E-07**	**6.43E-09**	**6.36E-08**	**1.82E-09**	**6.37E-08**	**1.51E-08**	**3.70E-07**	**2.02E-07**	**2.76E-05**	**6.34E-07**
**N1651-08**	**1.40E-07**	**6.43E-09**	**6.36E-08**	**1.82E-09**	**6.37E-08**	**1.51E-08**	**3.70E-07**	**2.02E-07**	**2.76E-05**	**6.34E-07**
**N1651-09**	**1.40E-07**	**6.43E-09**	**6.36E-08**	**1.82E-09**	**6.37E-08**	**1.51E-08**	**3.70E-07**	**2.02E-07**	**2.76E-05**	**6.34E-07**
**N1651-10**	**8.97E-08**	**1.29E-08**	**8.65E-08**	**5.68E-10**	**3.70E-08**	**2.76E-08**	**3.70E-07**	**2.02E-07**	**3.06E-05**	**7.05E-07**
**N1651-11**	**8.97E-08**	**1.29E-08**	**8.65E-08**	**5.68E-10**	**3.70E-08**	**2.76E-08**	**3.70E-07**	**2.02E-07**	**3.06E-05**	**7.05E-07**

Table S7
