# Supplementary material for: Elephant bones for the Middle Pleistocene toolmaker
Source: PLoS One. 2021 Aug 26;16(8):e0256090. doi: 10.1371/journal.pone.0256090 (PMC8389514; doi:10.1371/journal.pone.0256090)
Supplement: S4 File — (PDF) [file pone.0256090.s004.pdf]

Supporting Information

Elephant bones for the Middle Pleistocene toolmaker

Paola Villa*,Giovanni Boschian, Luca Pollarolo, Daniela Saccà, Fabrizio Marra, Sebastien Nomade, Alison Pereira.

Correspondence to: villap@colorado.edu

S4 File. Permissions from copyright holders

This PDF file includes:

Permission DEM (Digital Elevation Map)

INGV
terremoti
vulcani
ambiente
ISTITUTO NAZIONALE
DI GEOFISICA E VULCANOLOGIA

Rome, 4/21/2021

to: Paola Villa

Dear Paola Villa,

you have my permission to use and modify if necessary the Digital Elevation Map (DEM) for the region of Rome (WA 6570) for your research and to publish the image under a CC BY 4.0 open access license in all formats (i.e. print and digital).

Dr. Fabio Florindo

Director, Environment Department
Istituto Nazionale di Geofisica e Vulcanologia
Rome - Italy
fabio.florindo@ingv.it

Sezione RM1
Sismologia e Tettonofisica
Via di Vigna Murata, 605
00143 ROMA | Italia
Tel.: +39 06518601
Fax: +39 0651860507
aoo.roma1@pec.ingv.it
www.roma1.ingv.it
