# Supplementary material for: Elephant bones for the Middle Pleistocene toolmaker
Source: PLoS One. 2021 Aug 26;16(8):e0256090. doi: 10.1371/journal.pone.0256090 (PMC8389514; doi:10.1371/journal.pone.0256090)
Supplement: S5 File — (PDF) [file pone.0256090.s005.pdf]

Supporting Information

Elephant bones for the Middle Pleistocene toolmaker

Paola Villa*,Giovanni Boschian, Luca Pollarolo, Daniela Saccà, Fabrizio Marra, Sebastien Nomade, Alison Pereira.

Correspondence to: villap@colorado.edu

S5 File. Behavioral complexity of the Castel di Guido assemblage

This PDF file includes:

Regional Tradition

Humans and elephants co-existence

We consider two general features of the Castel di Guido assemblage, social transmission and the acquisition of large carcasses.

Regional tradition

Recent advances in dating show that four sites in Latium (Castel di Guido, Fontana Ranuccio, Malagrotta and Lademagne) are very close in time. Do these common occurrences of flaked bone artifacts represent a regional tradition reflecting a set of socially transmitted habits between groups in the same region? Several other sites in Latium have yielded bone tools but these are not from controlled excavation or have not undergone complete technological and taphonomic analysis. The term tradition may seem inappropriate as it implies a strong regionalization of cultural features and a stable pattern of intergenerational transmission. The term seems too specific to apply to rather variable and often isolated cases of bone tool-making. Yet a degree of technological continuity between these occurrences can be seen in other aspects of technology such as the selection and use of small flint pebbles and core-like pieces as blanks for retouched small tools. The almost exclusive preference for flint and the preference for thick blanks (including negative blanks) imply a technical repertory that was transmitted and shared by the Acheulian toolmakers in the region.

Humans and elephants co-existence

Remains of elephants and mammoths (both in the Elephantidae family) have been found at a number of archaeological sites of the Lower, Middle and early Late Pleistocene age. We limit our mentions to kill or scavenging sites with single or multiple elephant carcasses in Eurasia, especially sites where elephants are the dominant animal of the faunal assemblage.

Association of elephants and stone artifacts have been found at Gesher Benot Ya'kov in Israel, Notarchirico in southern Italy, Campitello in Tuscany, the sites in Latium listed on Table 1, Torralba, Ambrona, Aridos 1, Aridos 2 and Arrriaga IIa in Spain, Ebbsfleet in England, La Cotte de St. Brelade (Jersey, English Channel Islands) dated to MIS 6, Lehringen in Germany dated to MIS 5e, and Preresa in Spain dated to MIS 5a [40, 41, 78].

With the exception of two sites (La Cotte and Lehringen) none of these sites provide good evidence of hunting; hominids may have butchered remains from natural death. Good evidence of hunting might be the presence of weapons, e.g.the spear made of yew

wood, found among the rib of an adult *Paleoloxodon antiquus*, together with 25 artifacts, at the site of Lehringen in eastern Germany [40, 79]. At La Cotte, two separate accumulations of *Mammuthus* (MNI = 7 and 11) and *Coelodonta antiquitatis* (woolly rhinoceros; MNI = 5 and 3) are found in two different layers. The topographic setting (base of a deep ravine) suggests that the dangerous animals had been driven off the cliff (80; contra 81). These were rapid accumulations with no evidence of subaerial weathering.

Castel di Guido cannot support a hypothesis of hunting. A hypothesis of butchering of elephant remains from natural death at a source of water is more likely since elephants are heavily dependent on water sources. The evidence of butchering is supported by few cutmarks (Fig. S9); there are only three cutmarks identified by microscopic analysis (two on ribs and one on a long bone fragments). Their scarcity is undoubtedly due to the destruction of identifiable cut mark features by postdepositional abrasion [20].
