# Supplementary material for: Elephant bones for the Middle Pleistocene toolmaker
Source: PLoS One. 2021 Aug 26;16(8):e0256090. doi: 10.1371/journal.pone.0256090 (PMC8389514; doi:10.1371/journal.pone.0256090)
Supplement: S6 File — (PDF) [file pone.0256090.s006.pdf]

References (55-81) for Supporting Information
References with numbers 1-54 are in the main text

55. Anzidei AP, Villa P, Cerilli E (1993) La Polledrara di Cecanibbio (Roma). Dati preliminari sull'analisi tafonomica dei reperti faunistici. In: Preistoria e Protostoria in Etruria. Atti del secondo Incontro di Studi, Farnese, pp. 27-35.
56. Gaudzinski S, Turner E, Anzidei AP, Alvarez-Fernandez E, Arroyo-Cabrales J, Cinq-Mars J, et al. (2005) The use of Proboscidean remains in every-day Palaeolithic life. Quaternary International 126–128: 179–194.
57. Haidle MN, Pawlik AF (2010) The earliest settlement of Germany: Is there anything out there? Quaternary International 223-224: 143-153.
58. Brasser, M. (2020). Evaluating the extent of human influence at the Lower Palaeolithic site of Bilzingsleben. In: García-Moreno, A., Hutson, J. M., Smith, G. M., Kindler, L., Turner, E., Villaluenga, A., Gaudzinski-Windheuser, S. (Eds.) Human behavioural adaptations to interglacial lakeshore environments, Heidelberg: Propylaeum, 2020 (RGZM – Tagungen, Vol. 37). https://doi.org/10.11588/propylaeum.647
59. Tromnau G (1983) Ein Mammutknochen-Faustkeil aus Rhede, Kreis Borken (Westfalen). Archäologisches Korrespondenzblatt 13: 287–289.
60. Cahen D, Haesaerts P, Van Neer W, Van Pamel P (1979) Un outil en os du Paléolithique inférieur dans la nappe alluvial de Mesvin. Helinium XIX: 105-127.
61. Aguirre E (2005) Industria osea primitiva de Torralba. Munibe Antropologia-Arkeologia 57: 19-52.
62. Santonja M, Pérez-González A, Panera J, Rubio-Jara S, Sesé C, Soto E, Sánchez-Romero L (2014) Ambrona and Torralba archaeological and paleontological sites, Soria Province. In: Sala Ramos R, Carbonell E, Bermudez de Castro JM, Arsuaga JL, editors. Pleistocene and Holocene hunter-gatherers in Iberia and the Gibraltar strait. The current archaeological record, (Burgos-Fundacion Atapuerca) pp. 517-527.
63. Santonja M, Rubio-Jara S, Panera J, Sanchez-Romero L, Tarriño A, Pérez-González A (2018) Ambrona revisited: The Acheulean lithic industry in the Lower Stratigraphic Complex. Quaternary International 480: 96-117.
64. Dominguez-Rodrigo M (2005) "Artefactos" oseos en Torralba y Ambrona: studio de piezas sobre hueso post-craneal depositadas en el Museo Arquelogicos Nacional. In: Zona Arqueologica. Los Yacimentos paleoliticos de Amvrona y Torralba (Soria) 5: 282-287.
65. Tourloukis V, Thompson N, Panagopoulou E, Giusti D, Konidaris GE, Karkanas P, Harvati K (2018) Lithic artifacts and bone tools from the Lower Palaeolithic site Marathousa 1, Megalopolis, Greece. Preliminary results. Quaternary International 497: 47-64.
66. Zutovski K, Barkai R (2016) The use of elephant bones for making Acheulian handaxes: A fresh look at old bones. Quaternary International 406: 227-238.
67. Pereira A, Monaco L, Marra F, Nomade S, Gaeta M., Leicher N, et al. (2020) Tephrochronology of the central Mediterranean glacial Termination V and MIS 11c (~445-395 ka): new constraints from Vico volcano and Tiber valley, Central Italy. Quaternary Science Reviews 243. https://doi.org/10.1016/j.quascirev.2020.106470
68. Renne PR, Mundil LR, Balco G, Min K, Ludwig KR (2011) Joint determination of 40K decay constants and 40Ar*/40K for the Fish Canyon sanidine standard, and improved accuracy for 40Ar/39Ar geochronology. Response to the comment by W.H. Schwarz et al. Geochimica Cosmochimica Acta 75: 5097-5100.
69. Niespolo EM, Rutte D, Deino A, Renne PR (2017) Intercalibration and age of the Alder Creek sanidine 40Ar/39Ar standard. Quaternary Geochronology 39: 205-213. http://dx.doi.org/10.1016/j.quageo.2016.09.004

70. Lee JY, Marti K, Severinghaus JP, Kawamura K, Hee-−Soo Y, Lee JB, Kim JS (2006) A redetermination of the isotopic abundances of atmospheric Ar. Geochimica et Cosmochimica Acta 70: 4507-4512. doi:10.1016/j.gca.2006.06.1563
71. Marra F, Castellano C, Cucci L, Florindo F, Gaeta M, Jicha B, et al. (2020) Monti Sabatini and Colli Albani: the dormant twin volcanoes at the gates of Rome. Scientific Reports 10:8666. https://doi.org/10.1038/s41598-020-65394-2
72. Marra F, Gaeta M, Jicha BR, Nicosia C, Tolomei C, Ceruleo, et al. (2019) MIS 9 to MIS 5 terraces along the Tyrrhenian Sea coast of Latium (central Italy): assessing interplay between sea-level oscillations and tectonic movements, Geomorphology 346: 106843. DOI:10.1016/j.geomorph.2019.106843
73. Karner DB, Marra F, Renne PR. (2001) The history of the Monti Sabatini and Alban Hills volcanoes: groundwork for assessing volcanic-tectonic hazards for Rome. Journal of Volcanology and Geothermal Research 107: 185-219.
74. Marra F, Sottili G, Gaeta M, Giaccio B, Jicha B, Masotta M, et al. (2014) Major explosive activity in the Sabatini Volcanic District (central Italy) over the 800-390 ka interval: geochronological - geochemical overview and tephrostratigraphic implications. Quaternary Science Reviews 94: 74-101. doi:10.1016/j.quascirev.2014.04.010
75. Petronio C, Di Stefano G, Kotsakis T, Salari L, Marra F, Jicha B (2019) Biochronological framework for the late Galerian and early-middle Aurelian Mammal Ages of peninsular Italy. Geobios 53: 35-50. 10.1016/j.geobios.2019.02.002
76. Cassoli PF, De Giuli C, Radmilli AM, Segre AG (1982) Giacimento del Paleolitico inferiore a Malagrotta (Roma). Atti della XXIII Riunione Scientifica dell'Istituto Italiano di Preistoria e Protostoria, Il paleolitico inferiore in Italia, Firenze 7-9 maggio 1980, Istituto Italiano di Preistoria e Protostoria, Firenze, pp. 531-549. doi.org/10.1016/j.quaint.2019.01.001
77. Marra F, Florindo F (2014) The subsurface geology of Rome: sedimentary processes, sea-level changes and astronomical forcing. Earth-Science Reviews 136: 1-20. DOI:10.1016/j.earscirev.2014.05.001
78. Villa, P (2001). Early Italy and the colonization of Western Europe. Special Issue of Quat Int. 'Out of Africa' edited by O. Bar Yosef and L. Straus; 75 : 113-130.
79. Weber, T., (2000). The Eemian Elephas antiquus finds with artefacts from Lehringen and Gröbern: are they really killing sites? Anthropologie et Préhistoire 111, 177-185.
80. Scott K. 1980. Two hunting episodes of Middle Palaeolithic age at La Cotte de St. Brelade, Jersey (Channel Islands). World Archaeology 12: 137-152.
81. Scott B, Bates M, Bates R, Conneller C, Pope M, Shaw A, Smith G. (2014) A new view from La Cotte de St Brelade, Jersey. Antiquity 88 (339):13-29.
